# CRISPR-UnLOCK: Multipurpose Cas9-Based Strategies for Conversion of Yeast Libraries and Strains

**DOI:** 10.3389/fmicb.2017.01773

**Published:** 2017-09-20

**Authors:** Emily Roggenkamp, Rachael M. Giersch, Emily Wedeman, Muriel Eaton, Emily Turnquist, Madison N. Schrock, Linah Alkotami, Thitikan Jirakittisonthon, Samantha E. Schluter-Pascua, Gareth H. Bayne, Cory Wasko, Megan Halloran, Gregory C. Finnigan

**Affiliations:** ^1^Department of Biochemistry and Molecular Biophysics, Kansas State University Manhattan, KS, United States; ^2^Department of Anatomy and Physiology, College of Veterinary Medicine, Kansas State University Manhattan, KS, United States; ^3^Department of Biology, Kansas State University Manhattan, KS, United States

**Keywords:** CRISPR, Cas9, budding yeast, libraries, marker-less integration, gene drive, gene tagging

## Abstract

*Saccharomyces cerevisiae* continues to serve as a powerful model system for both basic biological research and industrial application. The development of genome-wide collections of individually manipulated strains (libraries) has allowed for high-throughput genetic screens and an emerging global view of this single-celled Eukaryote. The success of strain construction has relied on the innate ability of budding yeast to accept foreign DNA and perform homologous recombination, allowing for efficient plasmid construction (*in vivo*) and integration of desired sequences into the genome. The development of molecular toolkits and “integration cassettes” have provided fungal systems with a collection of strategies for tagging, deleting, or over-expressing target genes; typically, these consist of a C-terminal tag (epitope or fluorescent protein), a universal terminator sequence, and a selectable marker cassette to allow for convenient screening. However, there are logistical and technical obstacles to using these traditional genetic modules for complex strain construction (manipulation of many genomic targets in a single cell) or for the generation of entire genome-wide libraries. The recent introduction of the CRISPR/Cas gene editing technology has provided a powerful methodology for multiplexed editing in many biological systems including yeast. We have developed four distinct uses of the CRISPR biotechnology to generate yeast strains that utilizes the conversion of existing, commonly-used yeast libraries or strains. We present Cas9-based, marker-less methodologies for (i) N-terminal tagging, (ii) C-terminally tagging yeast genes with 18 unique fusions, (iii) conversion of fluorescently-tagged strains into newly engineered (or codon optimized) variants, and finally, (iv) use of a Cas9 “gene drive” system to rapidly achieve a homozygous state for a hypomorphic query allele in a diploid strain. These CRISPR-based methods demonstrate use of targeting universal sequences previously introduced into a genome.

## Introduction

*Saccharomyces cerevisiae* (budding yeast) continues to serve as an excellent model Eukaryote to study many biological phenomena and conserved molecular pathways. Part of the success and profound contributions of this single-celled Ascomycete is the ease by which its genome can be edited and the plethora of molecular tools that have been developed and expanded over the last few decades. Since *S. cerevisiae* can (i) uptake exogenous DNA (plasmids and amplified PCR fragments) very easily, (ii) assemble circular plasmids *in vivo*, and (iii) integrate engineered constructs into its genome with high fidelity, this has led to this organism being the world's most genetically tractable system. Yeast has provided a platform for the development of new technologies, such as the two- and three-hybrid systems (Fields and Song, 1989; Vidal and Fields, 2014; Maruta et al., 2016), synthetic genetic array (SGA) (Tong et al., 2001), and the (ongoing) synthesis/engineering of the first Eukaryotic genome *de novo* (Shen et al., 2017; Xie et al., 2017). Yeast has provided a system to study evolution (Hope et al., 2017), cellular aging (McCormick et al., 2015), biofuel development (Kim et al., 2017), drug production (Galanie et al., 2015), and human genetic diseases (Mayfield et al., 2012), to name only a few.

Part of the success of this model organism includes the development and utility of genome-wide libraries—collections of separate yeast strains each containing a unique modification (an engineered plasmid, an integrated epitope tag, or deletion of a gene, etc.)—that can be used to screen all non-essential (or essential) genes that would be required for different molecular processes. Over the years, the set of available yeast libraries has expanded to include epitope tags (Ross-Macdonald et al., 1999; Ghaemmaghami et al., 2003), over-expression arrays (Sopko et al., 2006; Ho et al., 2009), fluorescent protein fusions (Huh et al., 2003), gene deletions (Giaever et al., 2002), and essential hypomorphic alleles (Breslow et al., 2008; Li et al., 2011). These collections have been useful in uncovering cellular, biochemical, and genetic interactions across numerous fields. The power of yeast genetics, libraries, and robotic automation has recently been demonstrated in the world's largest collection of double deletion mutants (>20 million yeast strains) in a single study (Costanzo et al., 2016). However, construction of such a large collection (~5,000 separate strains) still presents many logistical challenges.

Previous studies have provided many collections of tagging “cassettes” for direct fusion of a gene or gene fragment (usually a fluorescent protein (FP) or biochemical epitope tag) to an endogenous open reading frame (Schneider et al., 1995; Longtine et al., 1998; Knop et al., 1999; De Antoni and Gallwitz, 2000; Janke et al., 2004; Sung et al., 2005; Tagwerker et al., 2006; Moqtaderi and Struhl, 2008). However, these methods all require two common components: (i) individual oligonucleotides to be purchased (or synthesized *de novo*) *per* locus being targeted and (ii) the use of a selectable marker (auxotrophic marker or drug selection marker) to identify and screen for proper isolates (Gardner and Jaspersen, 2014). While this strategy has been applied universally by nearly all yeast laboratories for several decades, the utility of this system can become limited in certain scenarios. First, tagging of large sets (or the entire genome) is often extremely cost-prohibitive (libraries are either purchased or shared at major academic centers). Second, tagging of a new gene using this cassette-based methodology in a strain which already contains one or more other tagged loci can present problems with the efficiency of targeting and integration as well as restrictions placed on the available selection marker(s). In some cases, a “marker-less” system may be more appropriate, but this typically requires two or more additional steps and does not guarantee a completely “scar-less” integration event. Third, the majority of available cassettes focus primarily on biochemical epitopes and/or fluorescent protein fusions. While these are useful for many molecular assays, there are additional fusions of interest that can be used as genetic screening tools, biochemical assays, or subcellular localization signals that have not been included in any previous methodological study.

The repurposing of the *Streptococcus pyogenes* CRISPR/Cas9 gene editing system (Jinek et al., 2012) has provided the entire field of molecular biology with a powerful method to target and manipulate precise DNA sequences in *any* genome. Ironically, this technology has been met with a lukewarm reception by the yeast community, given that powerful molecular toolkits and methods already exist, and decades worth of strains and libraries have already been generated. However, the CRISPR system is still being utilized in some yeast laboratories for unique editing applications such as chromosome splitting (Sasano et al., 2016), transcriptional modulation (Jensen et al., 2017), automated library construction (Si et al., 2017), and metabolic engineering (Ryan and Cate, 2014). Briefly, expression of the type II CRISPR nuclease Cas9, coupled with a single stranded fragment of RNA (single guide), allows for the protein/RNA complex to be recruited to the corresponding DNA sequence within any genome of interest. There, Cas9 induces a double stranded break (DSB) at the matching site that is anchored by a 3 bp protospacer adjacent motif (PAM) sequence. Cells respond by either performing (i) non-homologous end joining (NHEJ) to directly fuse the broken chromosome fragments or (ii) homology directed repair (HDR) using a donor DNA fragment (amplified PCR product) containing homologous sequences flanking either side of the break (Jinek et al., 2013). Cas9-dependent introduction of a DSB allows for deletion, replacement, or modification of existing DNA sequences in all genomes tested thus far, including budding yeast (DiCarlo et al., 2013).

In this study, we describe four independent Cas9-based methodologies for the introduction of both N- and C-terminal tags into budding yeast that combine the use of a universal targeting strategy based on a single sgRNA construct with strains from various yeast library collections. Our method, CRISPR-UnLOCK (Universal yeast Library Optimization and Conversion Kit), provides (i) precise targeting and integration in the absence of any selectable marker, (ii) a collection of 18 C-terminal tags that span a gamut of cellular localization signals, fusions, fluorescent markers, and biochemical epitope tags, (iii) a system nearly void of any “unique” oligonucleotides—targeting multiple loci can be accomplished with the same universal set of DNA primers, (iv) the ability to multiplex to multiple loci simultaneously, and (v) a strategy for “upgrading” of existing fluorescent proteins (FP) with an optimized codon bias or newly engineered/discovered FP. Furthermore, we demonstrate the use of a unique Cas9 arrangement—the “gene drive”—to achieve a homozygous diploid state for a query allele without the need for isolation of haploids (typically by the SGA method or traditional yeast spore isolation). This molecular toolkit provides powerful options to the conversion of existing yeast strains and/or libraries into new sets. This technology can be used for the construction of individual strains, small collections, or possibly, entire libraries. Our system is fully compatible and complementary with traditional cloning methods and screening techniques, such as SGA. There are many cloning scenarios that might benefit from a marker-less integration event. Finally, our CRISPR application should be widely applicable to other model systems in practice for cloning and targeting of “universal” genomic loci with a minimum number of guide RNAs.

## Materials and methods

### Yeast strains and plasmids

*S. cerevisiae* strains constructed and used in this study can be found in Table 1. Standard molecular biology techniques were used to manipulate all DNA and yeast (Sambrook and Russell, 2001). Strains from the TAP tag collection (Ghaemmaghami et al., 2003) were obtained and tested as clonal isolates (on SD-HIS plates) by PCR amplifying the C-terminal portion of the tagged gene of interest, the TAP tag, and into the universal *ADH1* terminator (Bennetzen and Hall, 1982) with a high-fidelity polymerase (KOD Hot Start, EMD Millipore), purified (GeneJet PCR Purification Kit, Thermo Fisher Scientific), and confirmed by Sanger DNA Sequencing (Genscript). Yeast from the haploid genome deletion collection (*MAT***α**) (Giaever et al., 2002) were tested as clonal isolates on rich medium containing G418 (Life Technologies, Inc.), and the proper gene knockout confirmed by diagnostic PCR. For strains containing an integrated cassette at the endogenous *HIS3* locus (e.g., GFY-2613), the following general method was used for (i) creation of the assembled plasmid used as a template and (ii) transformation and integration into the yeast genome. First, *in vivo* plasmid assembly (Finnigan and Thorner, 2015) was used to generate a construct containing ~1,000 bp of *HIS3* 5′ UTR (from starting vector pGF-V769) followed by 449 bp of the *SHS1* 5′ UTR, GFP(S65T) sequence, the *ADH1* terminator, the Kan^R^ drug resistance MX cassette, and ~1,000 bp of *HIS3* 3′ UTR (to generate plasmid pGF-IVL1348) and was verified by DNA sequencing. Second, the entire assembled cassette including 196 bp of HIS3 5′ UTR and 151 bp of 3′ UTR was amplified (digested with *DpnI* overnight to remove the template plasmid) and transformed into WT BY4741 yeast (*his3*Δ*1*) using a modified lithium acetate protocol (Eckert-Boulet et al., 2012). G418-resistant yeast were selected as clonal isolates (and confirmed to also be sensitive on SD-LEU plates) and chromosomal DNA was confirmed using multiple diagnostic PCRs to the integrated cassette and flanking regions at the *HIS3* locus (outside the region used for integration) to generate GFY-2613. For strains harboring a modified gene cassette at a different locus (e.g., *CDC11* for GFY-2624), a similar strategy was used with several changes. The starting strain was BY4742 *cdc11*Δ*::Kan*^*R*^ (GFY-150) and because of the presence of the universal MX(t) sequence present in all of the Kan^R^ deletion cassettes (Goldstein and McCusker, 1999), the integration plasmid did not contain any *CDC11* 3′ UTR yet still utilized the 5′ UTR for homologous recombination. Finally, for the gene drive containing strains (GFY-2440 and GFY-2442), due to the large size of the integrated cassette (>10 Kb), rather than amplifying a single PCR fragment, two partially overlapping PCRs (~5 kb each) were generated and co-transformed into yeast using the entire assembled cassette on a plasmid as the DNA template (pGF-IVL1149). The integration event utilized the ~100 bp overlapping sequence between the two PCRs (within the Cas9 gene) to perform HR, and insert the entire sequence at the correct locus.

**Table 1:** Yeast strains used in this study.

**Strain**	**Genotype**	**References**
BY4741	*MATα his3Δ1 leu2Δ0 met15Δ0 ura3Δ0*	Brachmann et al., 1998
BY4742	*MATα his3Δ1 leu2Δ0 lys2Δ0 ura3Δ0*	Brachmann et al., 1998
GFY-42	BY4741; *CDC10::mCherry::ADH(t)::SpHIS5*	Finnigan et al., 2015b
GFY-330	BY4741; *CDC12::GFP::ADH(t)::Hyg^*R*^ + pCDC12::URA3* (pJT1622)	This study
GFY-1583	BY4741; *KEL1::TAP^a^::ADH(t)::SpHIS5*	TAP Tag Collection
GFY-1589	BY4741; *BUD3::TAP^a^::ADH(t)::SpHIS5*	TAP Tag Collection
GFY-1620	BY4741; *ELM1::TAP^a^::ADH(t)::SpHIS5*	TAP Tag Collection
GFY-2047	BY4741; *CAF120::TAP^a^::ADH(t)::SpHIS5*	TAP Tag Collection
GFY-2056	BY4741; *NBA1::TAP^a^::ADH(t)::SpHIS5*	TAP Tag Collection
GFY-2069	BY4741; *BEM2::TAP^a^::ADH(t)::SpHIS5*	TAP Tag Collection
GFY-2071	BY4741; *MYO4::TAP^a^::ADH(t)::SpHIS5*	TAP Tag Collection
GFY-2092	BY4741; *BUD2::TAP^a^::ADH(t)::SpHIS5*	TAP Tag Collection
GFY-2251^b^	BY4742; *mso1Δ::Kan^*R*^*	Genome Deletion Collection
GFY-2259^b^	BY4742; *bni4Δ::Kan^*R*^*	Genome Deletion Collection
GFY-2256^b^	BY4742; *apl1Δ::Kan^*R*^*	Genome Deletion Collection
GFY-2613^c^	BY4741; *his3Δ::prSHS1::GFP(S65T)::ADH(t)::Kan^*R*^*	This study
GFY-2615^c^^,^ ^d^	BY4741; *his3Δ::prSHS1::eGFP::ADH(t)::Kan^*R*^*	This study
GFY-2617^e^	BY4741; *his3Δ::prCDC12::GFP(S65T)::ADH(t)::Kan^*R*^*	This study
GFY-2621^c^	BY4741; *his3Δ::prSHS1::mCherry::ADH(t)::Kan^*R*^*	This study
GFY-2622^e^	BY4741; *his3Δ::prCDC12::mCherry::ADH(t)::Kan^*R*^*	This study
GFY-2440^f^	BY4741; *cdc11Δ::cdc11(357–415Δ)::mCherry::CDC10 3*′*UTR::prGAL1/10::SpCas9::NLS::SHS1 3*′*UTR::prCCW12::Kan^*R*^ + (pGF-IVL1146;* pRS316; *prCDC11::CDC11(WT))*	This study
GFY-2442^f^	BY4742; *cdc11Δ::cdc11(357–415Δ)::mCherry::CDC10 3*′*UTR::prGAL1/10::SpCas9::NLS::SHS1 3*′*UTR::prCCW12::Kan^*R*^ + (pGF-IVL1146;* pRS316; *prCDC11::CDC11(WT))*	This study
GFY-2625^g^	BY4741; *cdc11Δ::CDC11(WT)::GFP::ADH(t)::SpHIS5* + *(pSB1/JT1520;* pRS316; *prCDC11::CDC11(WT))*	This study
GFY-2624^g^	BY4742; *cdc11Δ::CDC11(WT)::GFP::ADH(t)::SpHIS5* + *(pSB1/JT1520;* pRS316; *prCDC11::CDC11(WT))*	This study

a *The TAP (tandem affinity purification) tag consists of a linker sequence (11 residues), CBP domain (26), linker (9), TEV cleavage site (7), linker (10), first Protein Z domain (58), second Protein Z (58), and final linker (6). The two Protein Z domains are identical in sequence. All TAP-tag strains were tested as single clonal isolates; the genomic loci that were tagged were PCR amplified and confirmed via Sanger sequencing including (roughly) the last 200 bp of the tagged gene. The nine genes chosen occur on nine separate yeast chromosomes*.

b *Strains from the haploid genome deletion collection were confirmed as single clonal isolates for resistance to G418 disulfide and proper knock-out of the intended gene by diagnostic PCRs*.

c *Contains 449 bp of SHS1 5′ UTR. Strain GFY-2613 was constructed by PCR amplifying the prSHS1::GFP(S65T)::ADH(t)::Kan^R^ fragment from pGF-IVL1348 along with 500 bp of flanking homology engineered upstream of prSHS1 and downstream of the Kan^R^ cassette and transforming into WT BY4741 yeast. Strains GFY-2615, GFY-2621, and GFY-2622 were constructed in a similar manner from pGF-IVL1350, pGF-IVL1352, and pGF-IVL1353, respectively*.

d *Enhanced GFP (eGFP) contains S65T, F64L, R88Q, and H239L*.

e *Contains 477 bp of CDC12 5′ UTR*.

f *The following strains were constructed by first adding pGF-IVL1146 to WT BY4741 (GFY-2442) or WT BY4742 (GFY-2440) yeast. This “covering vector” expresses WT CDC11 with 21 codons mutated from their native code to an alternative codon (without changing the final protein sequence). Second, the endogenous CDC11 was deleted by transforming this strain with a PCR fragment of cdc11Δ::Hyg^R^ with flanking 5′ and 3′ UTR (300 bp) amplified from a chromosomal preparation of GFY-155 and selecting for resistance to hygromycin and lethality on media containing 5-FOA (loss of CDC11 renders cells inviable at 30°C). Third, a plasmid was constructed using three rounds of subsequent in vivo ligation and homologous recombination in yeast (Finnigan and Thorner, 2015) to assemble prCDC11::cdc11(357-415Δ)::mCherry::CDC10 3′UTR::prGAL1/10::SpCas9::NLS::SHS1 3′UTR::prCCW12::Kan^R^ on pRS315 (pGF-IVL1149). S. pyogenes Cas9 (yeast codon bias, CAI = 0.92) contains a C-terminal SV40 nuclear localization signal (SRADPKKKRKV) and is under transcriptional control of the GAL1/10 promoter (814 bp). The cdc11(357-415Δ) mutant contains 13 residues with alternative codons near the N-terminus and contains the CDC10 terminator sequence (465 bp). Cas9 contains the SHS1 terminator sequence (486 bp). The Kan^R^ MX cassette has been modified to remove the P(tef) constitutive promoter with the native yeast CCW12 promoter (992 bp) which still allows for selection of resistance to G418 disulfide. This entire linear fragment containing both CDC11 and Cas9 was amplified in 2 separate PCR reactions with overlapping sequence within the central region of Cas9 and co-transformed into the cdc11Δ::Hyg^R^ yeast also expressing the aforementioned covering vector. The CDC11 promoter (330 bp) and MX(terminator) sequence (235 bp) provided homology to the native CDC11 locus to integrate the entire gene drive cassette. The Cdc11 C-Terminal Extension domain (residues 357-415; CTE) is not required for septin filament formation (Versele et al., 2004) yet causes a severe loss of function when paired with shs1Δ or other mutations such as bni5Δ (Finnigan et al., 2015a)*.

g *GFY-150 yeast (BY4742; cdc11Δ::Kan^R^) or GFY-153 (BY4741; cdc11Δ::Kan^R^) were transformed with a PCR fragment containing prCDC11::CDC11(WT)::GFP::ADH(t)::SpHIS5 (amplified from pGF-IVL1354) to create GFY-2624 and GFY-2625*.

Plasmids used in this study can be found in Table 2. *In vivo* plasmid assembly was used for construction of all vectors unless otherwise noted (Finnigan and Thorner, 2015). Briefly, a starting vector containing the promoter of interest was linearized by digestion (overnight) of a downstream restriction cut site (typically *NotI* or *SpeI*). Next, PCR fragments to be assembled were amplified with a high-fidelity polymerase and oligonucleotides with overhanging tails of identical sequence to the adjacent fragment sequence. Construction of the desired plasmid was performed in yeast by co-transformation of a linear vector and the appropriate PCR fragments followed by selection for either (i) re-circularization of the original vector (e.g., on SD-LEU) or (ii) the presence of a drug cassette (e.g., Kan^R^ MX on G418) on one of the included PCRs. Following harvesting from yeast, transformation into competent *E. coli* (TOP10, Life Technologies, Inc.), and plasmid isolation (GeneJet Miniprep Plasmid Isolation Kit, Thermo Fisher Scientific), constructs were screened by diagnostic PCR and confirmed via Sanger DNA Sequencing. For plasmids to be used as donor DNA templates for PCR amplification, an additional cloning into the TOPO II vector (pCR-Blunt II-TOPO, Life Technologies, Inc.) was performed according to the recommended protocol. Genes synthesized *de novo* (Genscript) were obtained in a pUC57 (Amp^R^) vector and were used as DNA templates for amplification of assembled fragments or used as donor DNA for Cas9-based integration. For construction of sgRNA-expressing plasmids, the following strategy was employed (Figure S1). Guide RNA expression was based on the RNA polymerase III *SNR52* promoter and *SUP4* terminator (DiCarlo et al., 2013). A previously designed sgRNA plasmid (Finnigan and Thorner, 2016) served as the template for the common components [*prSNR52*, tracrRNA, *SUP4*(t)] for all designed sgRNAs. Briefly, *in vivo* plasmid assembly (using two unique oligonucleotides that inserted the 20 bp target DNA sequence) was used to create the fully assembled sgRNA (e.g., to target the TAP sequence) in a *CEN*-based plasmid. Second, the sgRNA cassette was amplified and cloned into TOPO II. Third, the cassette was subcloned to a high-copy yeast plasmid (pRS425) using flanking restriction sites. DNA maps for plasmid constructs created in this study are included in Figure S2.

**Table 2:** Plasmids used in this study.

**Plasmid**	**Description**	**References**
pRS315	*CEN, LEU2*	Sikorski and Hieter, 1989
pRS316	*CEN, URA3*	Sikorski and Hieter, 1989
pRS425	*2μ, LEU2*	Christianson et al., 1992
pCR™-Blunt II-TOPO®	TOPO II; *pUC origin, Kanamycin^*R*^, Zeocin^*R*^*	Invitrogen, Life Technologies
pUC57^a^	*pUC origin, Ampicillin^*R*^*	Genscript
pGF-IVL845^b^	pRS315; *TAP(link)::1xFLAG::Linker::GFP(β11)::ADH1(term)::Kan^*R*^*	This study
pGF-IVL890^c^	pRS315; *TAP(link)::1xFLAG::Linker::GFP(β11)::SHS1(term)::Kan^*R*^*	This study
pGF-IVL985^d^	pRS315; *TAP(link)::1xFLAG::Linker::GFP(β11)::SHS1(term)::prCCW12::Kan^*R*^*	This study
pGF-TOPO+IVL1204^e^	TOPO II; *TAP(link)::1xFLAG::Linker::GFP(β11)::SHS1(term)::MX(term)*	This study
pGF-TOPO+IVL1205^f^	TOPO II; *TAP(link)::1xFLAG::Linker::SpeI(site)::6xHIS::SHS1(term)::MX(term)*	This study
pGF-TOPO+IVL1206^g^	TOPO II; *prCDC11::NotI(site)::TAP(link)::mCherry(opt)::SHS1(term)::MX(term)*	This study
pGF-TOPO+IVL1207C^g^^,^^h^	TOPO II; *prCDC11::NotI(site)::TAP(link)::Nanobody(opt)::SHS1(term)::MX(term)*	This study
pGF-TOPO+IVL1208	TOPO II; *prCDC11::NotI(site)::TAP(link)::GST::SHS1(term)::MX(term)*	This study
pGF-TOPO+IVL1209	TOPO II; *rCDC11::NotI(site)::TAP(link)::1xFLAG::Linker::3xHA::SHS1(term)::MX(term)*	This study
pGF-TOPO+IVL1302^i^	TOPO II; *prCDC11::NotI(site)::TAP(link)::NLS::SHS1(term)::MX(term)*	This study
pGF-TOPO+IVL1303^j^	TOPO II; *prCDC11::NotI(site)::TAP(link)::NES::SHS1(term)::MX(term)*	This study
pGF-TOPO+IVL1304	TOPO II; *prCDC11::NotI(site)::TAP(link)::1xMYC::SHS1(term)::MX(term)*	This study
pGF-TOPO+IVL1305	TOPO II; *prCDC11::NotI(site)::TAP(link)::MBP::SHS1(term)::MX(term)*	This study
pGF-TOPO+IVL1306	TOPO II; *prCDC11::NotI(site)::TAP(link)::BirA(R118G)::SHS1(term)::MX(term)*	This study
pGF-TOPO+IVL1307^k^	TOPO II; *prCDC11::NotI(site)::TAP(link)::CAAX::SHS1(term)::MX(term)*	This study
pGF-TOPO+IVL1309	TOPO II; *prCDC11::NotI(site)::TAP(link)::SNAP::SHS1(term)::MX(term)*	This study
pGF-TOPO+IVL1310^l^	TOPO II; *prCDC11::NotI(site)::TAP(link)::SpHIS5::SHS1(term)::MX(term)*	This study
pGF-TOPO+IVL1311^g^^,^^m^	TOPO II; *prCDC11::NotI(site)::TAP(link)::mScarlet(opt)::SHS1(term)::MX(term)*	This study
pGF-TOPO+IVL1379^g^	TOPO II; *prCDC11::NotI(site)::TAP(link)::eGFP(opt)::SHS1(term)::MX(term)*	This study
pGF-TOPO+IVL1380^g^	TOPO II; *prCDC11::NotI(site)::TAP(link)::ymUkG1(opt)::SHS1(term)::MX(term)*	This study
pGF-TOPO+IVL1381^g^^,^^n^	TOPO II; *prCDC11::NotI(site)::TAP(link)::eGFP(opt):Lact-C2::SHS1(term)::MX(term)*	This study
pGF-IVL1251^g^^,^^o^	pRS315; *prCDC11::eGFP(opt)::CDC10(term)::MX(term)*	This study
pGF-IVL1252^g^	pRS315; *prCDC11::eGFP(opt)::ADH1(term)::Hyg^*R*^*	This study
pGF-IVL1253^g^	pRS315; *prCDC11::ymUkG1(opt)::CDC10(term)::MX(term)*	This study
pGF-IVL1254^g^	pRS315; *prCDC11::ymUkG1(opt)::ADH1(term)::Hyg^*R*^*	This study
pGF-IVL1255^g^	pRS315; *prCDC11::mCherry(opt)::SHS1(term)::MX(term)*	This study
pGF-IVL1256^g^	pRS315; *prCDC11::mCherry(opt)::ADH1(term)::Hyg^*R*^*	This study
pGF-pUC57+TAP(30)-STOP-MX(term)^p^	pUC57; *TAP(link)::MX(term)*	This study
pGF-TOPO+IVL1334^q^^,^^r^	TOPO II; *prMX::prSHS1::GFP(β10)::Linker::SacI*	This study
pGF-TOPO+IVL1335^q^^,^^s^	TOPO II; *prMX::prCDC11::GFP(β10)::Linker::SacI*	This study
pGF-425+IVL1274^t^^,^^u^	pRS425; *prSNR52::sgRNA(Kan-v1)::SUP4(term)*	This study
pGF-425+IVL1275^t^^,^^u^	pRS425; *prSNR52::sgRNA(Kan-v2)::SUP4(term)*	This study
pGF-V799^t^^,^^v^	pRS425; *prSNR52::sgRNA(TAP)::SUP4(term)*	This study
pGF-425+IVL1276^t^^,^^u^	pRS425; *prSNR52::sgRNA(GFP)::SUP4(term)*	This study
pGF-425+IVL1277^t^^,^^u^	pRS425; *prSNR52::sgRNA(mCherry)::SUP4(term)*	This study
pGF-V789^w^	pRS316; *prGAL1/10::SpCas9::NLS::CDC10(term)*	This study
pGF-IVL1146^x^	pRS316; *prCDC11::CDC11*	This study
pGF-IVL1419	pRS315; *prCDC11::CDC11::GFP(S65T)::ADH(t)::Kan^*R*^*	This study
pGF-IVL1420	pRS315; *prCDC11::CDC11::eGFP::ADH(t)::Kan^*R*^*	This study
pGF-IVL1421	pRS315; *prCDC11::CDC11::mCherry::ADH(t)::Kan^*R*^*	This study
pGF-IVL1422	pRS315; *prCDC11::CDC11::eGFP(opt)::ADH(t)::Kan^*R*^*	This study
pGF-IVL1423	pRS315; *prCDC11::CDC11::ymUkG1(opt)::ADH(t)::Kan^*R*^*	This study
pGF-IVL1424	pRS315; *prCDC11::CDC11::mCherry(opt)::ADH(t)::Kan^*R*^*	This study

a *The TAP(link)::MX(term) sequence was synthesized de novo by Genscript (Piscataway, NJ) and subcloned into the EcoRV site of pUC57*.

b *The TAP(linker) sequence includes the first 10 amino acids (GRRIPGLINP) of the TAP tag. A flexible Gly-Ser rich linker of 25 amino acids follows the FLAG epitope (GSGAGGSPGGGSGGSGSSASGGSTS). Finally, the GFP(β11) strand (EKRDHMVLLEYVTAAGITDAS) precedes a STOP codon. The Kan^R^ cassette includes the standard P(tef) and T(tef) sequences*.

c *Identical to pGF-IVL845 except the ADH1(term) has been replaced with 485 bp of the SHS1 3′ UTR sequence*.

d *Identical to pGF-IVL890 except the P(tef) promoter sequence from the Kan^R^ cassette has been replaced with prCCW12 (992 bp) from yeast*.

e *The MX(term) or T(tef) from the Kan^R^ cassette (235 bp) was directly fused after the SHS1 3′ UTR sequence. Contains the same marker sequence as pGF-IVL890*.

f *A SpeI restriction site was inserted in-frame (residues TS)*.

g *The sequence (opt) has been optimized for expression in yeast*.

h *The Nanobody domain (117 residues) has been developed against GFP*.

i *The SV40 nuclear localization signal has the sequence SRADPKKKRKV*.

j *The nuclear export signal is LAKILGALDIN*.

k *The CAAX box motif is from yeast Ras2 with the sequence GSGGCCIIS*.

l *The S. pombe HIS5 gene is the phenotypic equivalent of S. cerevisiae HIS3*.

m *The mScarlet-I variant contains the mutation T74I and has a shorter maturation delay*.

n *The Lact-C2 domain (158 residues) of bovine Lactadherin binds phosphatidylserine*.

o *Contains 465 bp of the CDC10 3′ UTR*.

p *Contains a STOP codon immediately following the TAP(link) sequence*.

q *The N-terminal GFP(β10) strand contains the sequence MDLPDDHYLSTQTILSKDLN followed by 38-residue linker (DVGGGGSEGGGSGGPGSGGEGSAGGGSAGGGSKKKKAT). For plasmid assembly, the linker sequence used for tagging ended with “…GGGSKK.” The prMX/P(tef) sequence contains 381 bp from the Kan^R^ cassette*.

r *The SHS1 promoter contains 596 bp of 5′ UTR*.

s *The CDC11 promoter contains 503 bp of 5′ UTR*.

t *The sgRNA-expressing cassette was modeled after the Church Lab's plasmid (DiCarlo et al., 2013) and synthesized de novo (GenScript). The cassette includes 269 bp from SNR52 promoter (Pol III), the 20 bp target sequence (crisprRNA) followed by the 79-base pair sequence for the fused (“single-guide”) tracrRNA and 20 bp poly-T SUP4 terminator sequence. The target sequences were chosen based on minimal homology to the yeast genome using BLAST alignments (see Methods)*.

u *First, the sgRNA 20 base pair target (crisprRNA) sequence was generated de novo using in vivo ligation and homologous recombination in yeast onto a CEN-based yeast vector. Second, the sgRNA cassette was amplified and ligated into a TOPO II cassette (Invitrogen). Third, the cassette was subcloned to the high-copy pRS425 vector using flanking NotI/SpeI sites on the TOPO II vector*.

v *Following sgRNA construction using in vivo ligation, the cassette was subcloned to pRS425 using BamHI/XhoI sites*.

w *S. pyogenes Cas9 was cloned with a C-terminal SV40 NLS tag and was placed under control of the GAL1/10 promoter (814 bp) and the CDC10 terminator (465 bp) using in vivo ligation. Flanking NotI/SpeI sites were used to subclone Cas9 to pRS316*.

x *There is no terminator sequence after the CDC11 STOP codon. Four Putative Cas9 targeting sites (23 bp each) have been mutated to include synonymous substitutions to create a maximum mismatch to escape unintended Cas9 editing yet maintain WT protein sequence. The nucleotide changes include the +1 position of the CDC11 promoter, nucleotides 4–6, 9, 12, 15, 18, 21, 63, 66, 69, 72, 75, 81, 84, 1074, 1077, 1083, 1084, 1086, 1089, 1092, 1095, 1098, 1099, and 1101. A CDC11 gene was synthesized de novo (Genscript, Piscataway, NJ) with the appropriate changes*.

### Culture conditions

Yeast were grown on solid medium or liquid cultures that included rich YPD (2% peptone, 1% yeast extract, 2% dextrose), or synthetic based mixtures (yeast nitrogen base with ammonium sulfate) and the necessary amino acid supplements. Carbon sources included either dextrose (2%), galactose (2%), or a raffinose (2%) and sucrose (0.2%) mixture—filter sterilization was used (rather than autoclaving) on all sugar types.

### CRISPR/Cas9-based editing

Selection of Cas9 genomic targets was performed as follows (Figure S3). For target genes (Kan^R^, GFP, mCherry, and TAP tag), possible PAM sites (5′-NGG-3′) were identified (on either the coding or non-coding strand) and tested for their level of mismatch against the S288C yeast genome. First, a search using the NCBI Basic Local Alignment Search Tool database (BLAST) with the 3′ most 15 bp of each putative site including the 3 bp PAM sequence for the maximum mismatch presented in the yeast genome was performed. Previous work has demonstrated this “seed” region (and PAM) are most significant in genomic target specificity (Jinek et al., 2012; DiCarlo et al., 2013; Jiang et al., 2013). A preference for target sites included (i) sequences whose closest “match” in the yeast genome did not have a complete 3′ GG PAM sequence and/or (ii) contained many mismatches across the 15 bp target site. Second, the entire 23 bp target was also searched against the genome for the total number of mismatches. Third, additional non-yeast sequences (plasmid backbones, *S. pyogenes* Cas9 itself, drug cassettes, etc.) were scanned for a maximum mismatch level. This selection process reduces (or eliminates) the possibility of Cas9 “off-target” effects which can be a major concern for editing if similar site(s) are present within the genome of interest.

The *S. pyogenes* Cas9 was introduced into yeast strains on a *URA3*-based *CEN* plasmid under control of the inducible *GAL1/10* promoter in a first transformation event prior to any editing. This allows for (i) repression of Cas9 transcription when yeast metabolize dextrose, (ii) optional counter-selection on media containing 5-FOA, and (iii) stable propagation of the Cas9-containing plasmid in all yeast cells (2–3 rounds of selection in SD-URA medium) prior to addition of sgRNA-expressing plasmid and donor DNA. Repression of the *GAL1/10* promoter sequence has been previously documented (Flick and Johnston, 1990). Additionally, separation of the sgRNA-expressing plasmid and the Cas9 plasmid (added to yeast in a first transformation event) prevents any possible Cas9 proteins from editing (no guide sequence).

Activation of Cas9 and *in vivo* editing was performed as previously described (Finnigan and Thorner, 2016). Briefly, strains harboring the Cas9 plasmid (pGF-V789) were cultured overnight to saturation in S+Raffinose/Sucrose-URA, back-diluted to an OD_600_ of ~0.30 OD/mL and cultured for 4.5 h in YPGal at 30°C. Cells were harvested and transformed or co-transformed with 1,000 ng of sgRNA plasmid and, when appropriate, 1,000–1,500 ng of donor PCR DNA. For PCR fragments of slightly varied length, the appropriate amount of product was adjusted accordingly. Following a heat shock at 42°C for 45 min, cells were recovered at 30°C overnight in fresh YPGal, and plated onto selection media. For Cas9-based editing (with or without donor DNA), the selection plates used were SD-URA-LEU to select for the Cas9-based plasmid and the sgRNA-containing plasmid and incubated for 3 days at 30°C before imaging. The total number of viable yeast colonies was counted using a sectoring method in a single-blind protocol (researchers counting colonies were not aware of the genotype of each plate). Depending on the density of yeast colonies per plate, either 1/4, 1/8, or 1/16 of the plate was sectored and individual colonies were counted manually and extrapolated for the entire surface of the plate (for smaller sectors, two or more separate regions were tallied and averaged prior to extrapolation).

Confirmation of Cas9 editing (via NHEJ or by HDR) was accomplished by (i) testing colonies from the transformation plates on selection media (e.g., SD-HIS) to assay for absence of the deleted selectable marker and (ii) obtaining clonal isolates with the correct growth phenotypes on SD-URA-LEU plates, and (iii) preparing chromosomal DNA and PCR amplification with a high-fidelity polymerase. Finally, diagnostic PCRs and DNA sequencing confirmed the presence (or absence) of the appropriate gene fragments.

### Cas9 gene drive and containment

Experiments with the Cas9-based gene drive were performed using the following protocol. First, the Cas9-containing gene drive cassette was integrated into a haploid yeast strain. Since the affected allele being tested (*CDC11*) is an essential gene, a *URA3*-based covering vector (pGF-IVL1146) expressing WT *CDC11* was also present in these strains. Second, the gene drive haploid strains were transformed with the sgRNA-expressing plasmid but were cultured and maintained in the presence of dextrose (to continually repress Cas9 transcription). Third, yeast were selected twice on SD-URA-LEU medium before being mated to the sample “target” strains expressing *CDC11::GFP::ADH1(t)::SpHIS5* at the native *CDC11* locus of the opposite mating type. While we have engineered the entire gene drive system (covering plasmid and affected *CDC11* allele) to allow for the introduction of sgRNAs targeting the WT *CDC11* gene coding sequence, we are compelled to demonstrate use of the gene drive using a partially “artificial” target (in this case, GFP fused to the WT *CDC11* gene) for safety and ethical reasons. Fourth, following mating on rich medium, yeast were transferred (replica-plating on sterile velvet cloths) to SD-URA-LEU-HIS plates to select for diploid formation (and to maintain the *URA3*-covering vector). Three consecutive rounds of diploid selection (dextrose) were performed to ensure the absence of any haploids and concurrent inactivation of the Cas9 drive—moreover, even upon rare expression of Cas9, there would be no target DNA sequence to initiate editing. Fifth, yeast were cultured overnight in pre-induction media to saturation as previously described (raffinose/sucrose mixture) selecting for the *URA3*- and *LEU2*-containing plasmids. Sixth, cells were back-diluted and cultured in YPGal for 24 h at 30°C. Seventh, yeast were centrifuged, washed in 1 mL of YPD, and diluted in sterile water to ~250–500 cells per mL, spread onto SD-URA-LEU plates, and incubated for 3 days. Finally, yeast were transferred from the recovery plates to various drop out or drug-containing media to test for the individual genotype(s) of single colonies.

A variety of safeguards were implemented to ensure proper, safe, and contained use of these yeast gene drive strains. Cultures containing the active (or pre-induced) gene drives were immediately heated to 75°C for a minimum of 3 h (and usually overnight) prior to washing with water. The heated cultures were rinsed with distilled water 3–4 times and all the liquid (including rinses) was collected and autoclaved for 45 min at >121°C. All plastic tubes, pipet tips, and any disposable material was autoclaved before disposal and velvet cloths (for replica-plating) were immediately autoclaved without rinsing. Agar plates containing any combination of the Cas9 drive and the sgRNA were only maintained until yeast were transferred to the next step in the protocol; older plates were autoclaved prior to disposal. All diploid strains generated from the gene drive experiments were not preserved or frozen but were immediately autoclaved and destroyed. With the only exception being the liquid YPGal induction, all agar plates used contained dextrose and actively repressed transcription of Cas9. The “target” selected (GFP) is not a native yeast gene, and must *also* be present at the *CDC11* locus for the drive to copy itself within a diploid genome. Moreover, the laboratory strain BY4741/BY4742 (BY4743) has been shown by others to be extremely inefficient at sporulation, even under optimal conditions that induce meiosis and spore formation (Heasley and McMurray, 2016). Finally, the high-copy pRS425 plasmid harbored the sgRNA cassette—without constant selection, this unstable plasmid is rapidly lost from yeast within several days (our unpublished results) and has been previously shown to be a useful safeguard to using gene drives in yeast (DiCarlo et al., 2015).

### Fluorescence microscopy

Yeast were grown to exponential phase (A_600_ of 1.0 OD/mL) in YPD culture at 30°C, harvested, washed with water, and prepared on standard microscope slides with a coverslip. Samples were imaged within 5–10 min of slide preparation on a Leica DMI6500 inverted fluorescence microscope (Leica Microsystems Inc., Buffalo Grove, IL) with a 100x objective lens, fluorescence filters (Semrock, GFP-4050B-LDKM-ZERO and mCherry-C-LDMK-ZERO). A Leica DFC340 FX camera, Leica Microsystems Application Suite AF software, and ImageJ (National Institute of Health) software were used to obtain and process all images. All images were treated identically and rescaled together. The yeast cell periphery was determined using either a DIC image or over-exposing a fluorescence image. Representative cells were chosen for each image. White light (DIC) was used to bring yeast into the plane of focus; equivalent exposure times were used for all images within a set.

## Results

### Limitations of commonly used marker swapping systems

Given the recent expansion and utility of CRISPR/Cas9 gene editing across many model systems, we sought to employ this technology to provide the yeast community with a new multipurpose molecular toolkit for strain construction. Previous work has provided a suite of useful gene-tagging cassettes for *S. cerevisiae* with the majority focused on either (i) biochemical epitope tags (Tagwerker et al., 2006; Moqtaderi and Struhl, 2008; Funakoshi and Hochstrasser, 2009) or (ii) fluorescent protein variants (Sheff and Thorn, 2004; Lee et al., 2013; Malcova et al., 2016). While these methodologies have been useful in both the tagging of individual genes (when the need arises) or, in some cases, the construction of entire yeast library collections, few studies (Sung et al., 2008, 2013) have provided similar cassettes for the use of modifying an *existing* library into a new/novel collection. Moreover, the current strategy for “swapping” of one tag or selectable marker for another is heavily centered around the use of the MX-based drug resistant cassettes developed nearly two decades ago (Longtine et al., 1998; Goldstein and McCusker, 1999). The utility of this cassette-based system lies in the common universal promoter and terminator from *A. gossypii* which allow for a common sequence among multiple drug or auxotrophic markers (Kan^R^, Nat^R^, *S. pombe HIS5*, etc.). For instance, creation of a Hyg^R^-tagged strain from the genome deletion collection (Kan^R^-marked) uses the flanking promoter and terminator sequences as anchoring homology for the marker-swapping event via homologous recombination. However, this alteration strategy presents an unintended barrier in the conversion of previously existing libraries that utilized the MX-based cassette strategy for construction (e.g., *GFP-ADH1-SpHIS5* or *TAP-ADH1-SpHIS5*).

**Figure 1:**
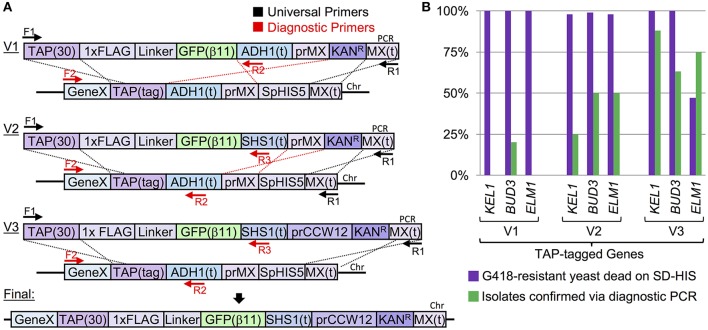
Chromosomal integration of a C-terminally tagged cassette into the TAP-tagged yeast library using homologous recombination. **(A)** Three constructs (V1, V2, and V3) were PCR amplified (from pGF-IVL845, pGF-IVL890, and pGF-IVL985, respectively) and transformed into three yeast strains (GFY-1583, GFY-1589, and GFY-1620) containing either *KEL1, BUD3*, or *ELM1* tagged with the TAP marker (Figure S3). These integration cassettes (Table 2) allow for a C-terminal 1xFLAG-Linker-GFP(β11) tripartite split GFP tag (Finnigan et al., 2016) to be fused to any open reading frame that is part of the TAP collection. Each PCR contains a 30 bp universal segment of the TAP linker sequence as well as the full MX terminator; black dotted lines illustrate the expected homologous sections with the chromosomal DNA. Additional identical sequences [e.g. *ADH1*(t)] also providing homology are illustrated with red dotted lines. Two universal primers (black arrows) amplify the common TAP linker sequence (F1, “TAP Tag clone out F”) and the MX(t) sequence (R1, “MX clone out R2”) (Table S1). Unique diagnostic primers, red arrows. Replacement of the prMX with the pr*CCW12* still allowed for G418 selection. **(B)** Quantification of the PCR integrations from **(A)** using both growth assays and diagnostic PCRs. G418-resistant yeast were tested on SD-HIS medium (*n* = 100 colonies). From SD-HIS sensitive colonies, isolates (V1, *n* = 10; V2/V3, *n* = 8) were selected and assayed by PCR as illustrated in **(A)**. For V1, PCRs [F2, “*KEL1* Internal +2908 F”/“*BUD3* Internal +4381 F”/“*ELM1* Internal +1455 F”; R2, “Internal *ADH1*(t) R”] were performed; for V2/V3, PCRs (F2/R2 and F2/R3, “*SHS1*(t) R”) were assayed (Table S1). At least two isolates for each integration event were confirmed via DNA sequencing.

We sought to illustrate this apparent conundrum and some of the limitations of the MX-based marker swapping methodologies most commonly used within the yeast community (Figure 1A). Given the yeast TAP (Tandem Affinity Purification) tagged yeast collection, we first designed an integrating cassette that would append the C-terminus of any particular gene present in the library with a 1xFLAG epitope, flexible linker, and the short GFP(β11) strand of the tripartite split GFP system (Cabantous et al., 2013; Finnigan et al., 2016; Figure 1A, top) and also included the common *ADH1* terminator and MX-based Kan^R^ cassette. This general methodology has been previously employed (Sung et al., 2008) and takes advantage of a short stretch of bases present at the 5′ end of the TAP sequence. Indeed, our system included exactly 30 base pairs of the TAP construct (10 residues in frame) that would serve as the source of upstream sequence homology. Additionally, the MX terminator sequence would serve as the region of downstream homology for recombination and integration of the entire cassette into the genome in place of the TAP tag and marker. However, our system (V1) provided a significant segment of internal homology [*ADH1*(t)-prMX; 627 bp] that could serve as an alternative HR source for the introduction of the desired selectable marker (Kan^R^) in place of the *SpHIS5* marker. Not surprisingly, while 100% of all G418-resistant colonies from the V1-integration event (Figure 1B) had replaced the *SpHIS5* marker within the genome, <7% of total randomly-selected isolates tested across three separate TAP-tagged strains (*KEL1, BUD3*, and *ELM1*) did not include any of the desired sequence *upstream* of the Kan^R^ cassette supporting the model that the internal homology present within our integration cassette provided a significant source of inappropriate homology that is greatly favored over the 5′ 30 bp desired TAP sequence. To further support this model, and to provide a means to bias the HR-based integration event toward the desired outcome, we designed a second cassette (V2) that replaced the *ADH1* terminator with the *SHS1* 3′ UTR sequence (reducing the internal homology to 389 bp). As expected, the percentage of isolates with the correct C-terminal tag and marker increased to roughly 40% (Figure 1B). Finally, by replacing the MX promoter sequence with the promoter of the constitutive *CCW12* 5′ UTR sequence (V3) where the internal homology was reduced to zero, the percentage of correct isolates was increased to 75%. While our V3 system is one “traditional” cloning solution to the issue of unintended cross-over, it is still limited in utility due to the reliance on the existing marker-based system.

While traditional HR-based strategies (Figure 1) can be employed to circumvent the issue of inappropriate cross-over with the MX-based tagging system, there remain some scenarios where a designed integration cassette system is not compatible with the yeast strain(s) to be manipulated. Examples of this marker “conundrum” include (i) repeated use of many (if not all) of the available drug resistance and nutritional markers within the genome already or (ii) markers present on selectable plasmids, or (iii) markers to be used for future methodologies (such as SGA diploid selection). Therefore, in these cases, a marker-less integration event would greatly aid in strain construction. Moreover, removal of an *existing* marker already present in the genome (e.g., TAP marked with *S. pombe HIS5*) increases the pool of available markers for future selection or construction. For these reasons, we sought to pilot various uses a CRISPR-based methodology given the availability of universal DNA sequences already present in many genome-wide libraries or laboratory collections.

### CRISPR/Cas9-based methodology for C-terminal marker-less gene tagging

**Figure 2:**
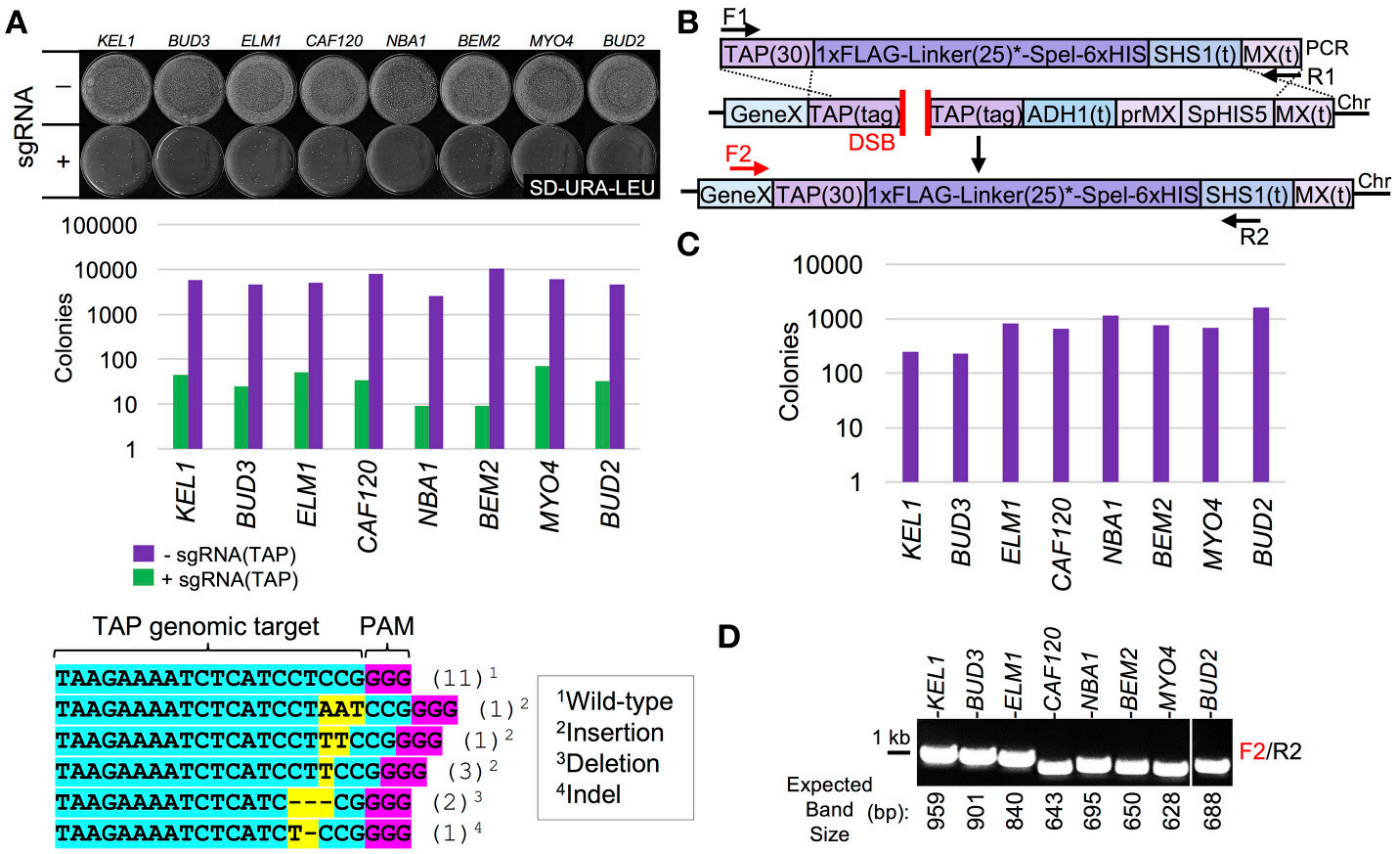
Use of CRISPR/Cas9 editing to C-terminally tag the TAP haploid library. **(A)** Targeting of Cas9 to the TAP tag sequence at various genomic loci induces NHEJ. Eight yeast strains from the TAP collection (GFY-1583, GFY-1589, GFY-1620, GFY-2047, GFY-2056, GFY-2069, GFY-2071, and GFY-2092) were (i) transformed with a Cas9 plasmid (pGF-V789), (ii) induced in galactose for Cas9 expression, (iii) transformed with the sgRNA plasmid (pGF-V799) targeting the TAP sequence (Figure S3) or an empty pRS425 control vector, and (iv) plated to SD-URA-LEU plates (top). The total number of colonies was quantified on a log_10_ scale (middle). Surviving colonies from the *KEL1, BUD3*, and *ELM1* transformation events (+sgRNA) were sequenced at their TAP-tagged loci (bottom). The number of each obtained genotype is illustrated. **(B)** As in Figure 1A, a C-terminal integration cassette containing a FLAG/His epitope tag and a 25-residue flexible linker (asterisk) (see Table 3) was constructed. The TAP(30) sequence contains the first 30 bp of the TAP tag cassette. **(C)** Strains from **(A)** were transformed with the Cas9 vector, the sgRNA(TAP) vector, and equimolar amounts (1,000 ng) of donor PCR DNA (F1, “TAP Tag clone out F”/R1, “MX clone out R2”), plated to SD-URA-LEU, and the total colony count quantified. **(D)** Colonies (*n* = 30–50) from **(C)** were selected, tested on SD-HIS medium, and a representative isolate (*n* = 1) was selected (lacking the *S. pombe HIS5* marker) and assayed by diagnostic PCR. (F2, Gene-specific primers/R2, “*SHS1*(t) R”) (see Table S1). The expected PCR sizes (bp) are shown.

We utilized the CRISPR system to provide a marker-swapping strategy where (i) an efficient integration success rate could be reliably achieved, (ii) a marker-less design could still be selected for with high fidelity, and (iii) the CRISPR components (Cas9 and the sgRNA) could be optionally removed from the designed strain following the editing event. Eight yeast strains from the TAP-tag collection representing genes present on eight separate yeast chromosomes were selected and transformed with a *URA3*-based *CEN*-vector harboring *S. pyogenes* Cas9 under control of the inducible *GAL1/10* promoter (Figure 2). Second, a target sequence within the TAP tag was chosen with a maximum mismatch to the yeast genome (Figure S3) and the appropriate sgRNA cassette was constructed on a high-copy *LEU2*-based vector (DiCarlo et al., 2013). Transformation of the sgRNA plasmid into Cas9+ yeast (Figure 2A) and subsequent selection on SD-URA-LEU medium demonstrated the efficiency of editing *in vivo*. Yeast transformed with an empty pRS425 vector yielded many thousands of colonies following selection; however, only a small number of viable yeast remained after editing by Cas9. It has been previously demonstrated that the DSB introduced by Cas9 is poorly tolerated in yeast (DiCarlo et al., 2013; Finnigan and Thorner, 2016). The remaining surviving colonies that had presumably undergone Cas9-based editing and subsequent NHEJ were selected and tested by DNA sequencing at their TAP-tagged loci. Indeed, while many isolates did not display any detectible alteration from the WT sequence (from either a lack of editing, or, more likely, editing and repair by NHEJ without any remaining “scar”), 8/19 isolates had deletions, insertions, or indels at the +3 position (the site of Cas9 cleavage) upstream of the PAM sequence (Figure 2A, bottom).

Given that editing by Cas9 in yeast is >99% lethal (the few remaining colonies likely to have been repaired via NHEJ or by escaping editing), we sought to combine the selection for cell viability with introduction of a C-terminal tagging cassette (Figure 2B). To further illustrate the utility of Cas9-based integration in yeast, we designed a sample C-terminal epitope tag cassette (1xFLAG-linker-6xHistidine) with the *SHS1* terminator and the MX terminator sequence. Similar to our initial methodology (Figure 1), the 30 bp TAP and MX(t) sequences served as the only regions with homology to the genome; these are also on flanking portions of the intended Cas9-induced DSB and allow for HR across the chromosomal break (Figure 2B). TAP-tagged strains expressing Cas9 were co-transformed with the sgRNA(TAP) plasmid as well as PCR-amplified donor DNA and selected on SD-URA-LEU medium—many hundreds of surviving colonies remained on each plate for each tested strain (Figure 2C). When individual isolates were tested for loss of the *SpHIS5* marker, between 75 and 100% of each randomly-chosen sample removed (and replaced) the endogenous marker (our unpublished data). Further analysis by diagnostic PCR (Figure 2D) confirmed these edited, viable colonies sensitive on SD-HIS plates had, in fact, integrated the intended epitope tag in place of the original TAP tag without any selectable marker present in 8 out of 9 strains tested: integration of the marker at the *EPO1* locus was not successful for the single isolate tested (our unpublished data). Through this study, we have maintained a consistent selection criteria for assaying of potentially edited yeast strains. As mentioned, we first assay for a loss of the current genomic marker (typically *S. pombe HIS5*). From clonal isolates that have lost this selection marker, we have chosen to only interrogate a single sample by diagnostic PCR and DNA sequencing to demonstrate the utility of our system in the absence of all traditional selectable markers and drugs. Subsequent to editing, expression of Cas9 can be repressed by growth on dextrose whereas loss of the plasmid harboring Cas9 can be achieved by selection on 5-FOA. In the absence of continual selection on medium lacking leucine, the high-copy plasmid containing the sgRNA cassette was rapidly lost (our unpublished results). Removal of sgRNAs on 2μ plasmids has also been previously documented (DiCarlo et al., 2015). While we recognize our system includes use of two markers (*URA3* for Cas9 and *LEU2* for the sgRNA), these constructs can be sub-cloned to commonly used vectors (pRS series, etc.), combined onto the same vector, or even integrated into the genome to generate a new parental laboratory strain.

**Figure 3:**
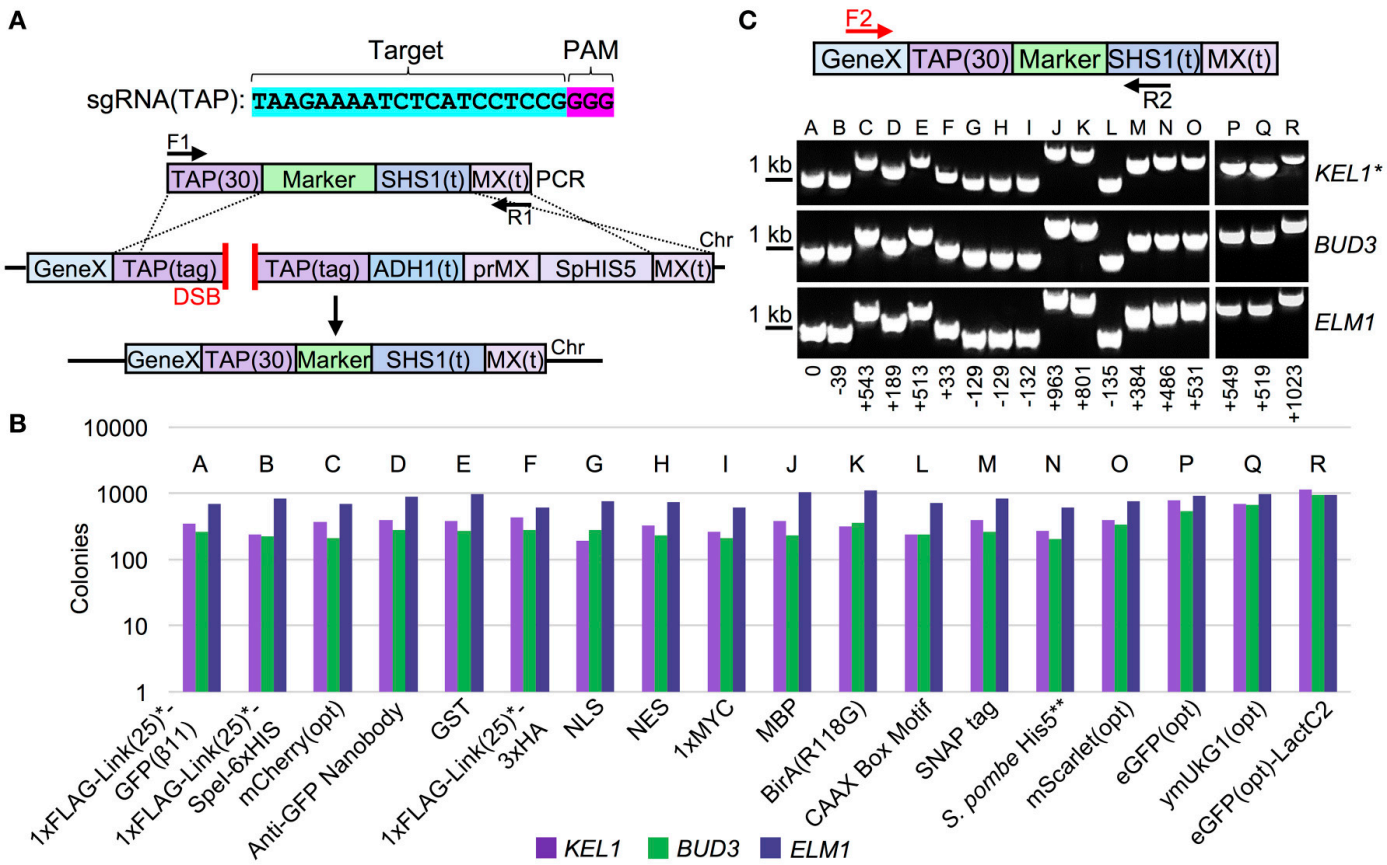
Collection of C-terminal marker-less tags for Cas9-based integration. **(A)** The proposed integration strategy illustrated in Figure 2A was used to construct 18 C-terminal peptide or protein fusions (Table 3). **(B)** TAP-tagged *KEL1, BUD3*, and *ELM1* (GFY-1583, GFY-1589, and GFY-1620) yeast containing Cas9 (pGF-V789) were co-transformed with the sgRNA(TAP) plasmid (pGF-V799) and equimolar amounts of PCR product (F1/R1) amplified from the 18 C-terminal tag constructs (Table 2), selected on SD-URA-LEU plates, and the total colony count quantified for each event. Loss of the native *S. pombe HIS5* marker was also assayed (Figure S4). **(C)** Clonal isolates (*n* = 1) from each integration (lacking the original *HIS5* marker) were assayed by diagnostic PCR (F2, Gene-specific F/R2, “*SHS1*(t) R”). The relative PCR fragment sizes (bp) are illustrated (setting the first band for PCR “A” as 0 bp). The predicted sizes for PCRs are provided (Table S2). Asterisk, the *KEL1* locus was confirmed by DNA sequencing for all 18 integrations.

To expand the utility of our Cas9-based system, we constructed 18 unique C-terminal tagged cassettes that can all be integrated in place of the TAP tag (Figure 3A, Table 3). While previous studies have focused primarily on either biochemical epitope tags or fluorescent proteins, we have provided a far more comprehensive molecular toolkit that should provide a wide range of options for biochemical, cellular, genetic, and microscopy-based assays. These include a variety of tags: (i) codon optimized versions of eGFP, coral ymUkG1, mCherry, and mScarlet, (ii) cellular localization signals such as a NLS, NES, and a CAAX box motif, (iii) a sampling of commonly used biochemical tags such as GST, MBP, (HA)_3_, and MYC, and (iv) unique protein fusions, such as an anti-GFP nanobody and a promiscuous BirA protein. To demonstrate the efficiency of Cas9-based, marker-less integration, three sample TAP-tag containing strains (*KEL1, BUD3*, and *ELM1*) were transformed with all 18 possible donor DNA sequences (Figure 3B). Given that identical conditions, sgRNA target sequences, and amplified cassettes were used, we observed slightly varied efficiencies based on the genetic locus being assayed—targeting and integration was most successful at *ELM1* locus with nearly 90–100% replacement for all donor DNAs tested (Figure S4). Importantly, this comparison—unlike the majority of other Cas9 editing studies—can be directly made across loci since the target sequence (TAP) and the sgRNA are *identical* and only the genomic placement differs between editing events. Only a single isolate that was pre-screened for loss of the native *S. pombe HIS5* marker was tested for each of the 54 integrations. We achieved a 100% success rate when verified by diagnostic PCR and DNA sequencing (Figure 3C, Tables S2, S4). For the *S. pombe HIS5* gene (in-frame) fusion with the target gene of interest, selection on SD-HIS could not be used to determine if the TAP cassette had been replaced since the resulting gene fusion may (or may not) result in a cytosol-presented, functional His5 (budding yeast His3 equivalent) protein (Kel1 and Bud3 differed from Elm1 in this respect). These results highlight the ability of Cas9-based editing to be coupled with swapping of an existing tagged (TAP) library to a variety of useful gene fusions in the absence of any selectable marker with high efficiency.

**Table 3:** Collection of C-terminal protein fusions for marker-less integration.

**Plasmid (pGF)**	**Marker(s)**	**Description**	**Size (amino acids)**	**References**
1204	1xFLAG-Linker (25)-GFP(β11)	Biochemical Tag + Split GFP Tag^a^	54	Hopp et al., 1988; Cabantous et al., 2013
1205	1xFLAG-Linker (25)-SpeI-6xHIS	Biochemical Tags^b^	41	Hochuli et al., 1988
1206	mCherry(opt)	Yeast Codon Optimized Fluorescent Protein^c^	235	Shaner et al., 2004
1207C	anti-GFP Nanobody	Anti-GFP Protein Tether^d^	117	Rothbauer et al., 2006; Kubala et al., 2010
1208	GST	Biochemical Tag^e^	225	Benard and Bokoch, 2002
1209	1xFLAG-Linker(25)-3xHA	Biochemical Tag^f^	65	Wilson et al., 1984; Field et al., 1988
1302	NLS	Cellular Localization^g^	11	Kalderon et al., 1984
1303	NES	Cellular Localization^h^	11	Xu et al., 2012
1304	1xMYC	Biochemical Tag^i^	10	Hilpert et al., 2001; Krauss et al., 2008
1305	MBP	Biochemical Tag^j^	375	Duplay et al., 1984
1306	BirA(R118G)	Proximity-Dependent Protein Biotinylation^k^	321	Choi-Rhee et al., 2004; Cronan, 2005
1307	CAAX Box Motif	Cellular Localization^l^	9	Mitchell et al., 1994
1309	SNAP tag	Fluorescent Protein^m^	182	Juillerat et al., 2003
1310	*S. pombe* His5	Cellular Growth Screening^n^	216	Longtine et al., 1998
1311	mScarlet(opt) WT	Yeast Codon Optimized Fluorescent Protein^o^	231	Bindels et al., 2017
1379	eGFP(opt)	Yeast Codon Optimized Fluorescent Protein^p^	237	Cinelli et al., 2000
1380	ymUkG1(opt)	Yeast Codon Optimized Fluorescent Protein^q^	227	Kaishima et al., 2016
1381	eGFP(opt)-LactC2	Yeast Codon Optimized Fluorescent Protein + Membrane tethering^r^	395	Andersen et al., 2000; Shao et al., 2008

a *FLAG Epitope, Flexible linker (GSGAGGSPGGGSGGSGSSASGGSTS), GFP(β-11) strand (EKRDHMVLLEYVTAAGITDAS)*.

b *FLAG Epitope, Flexible linker (GSGAGGSPGGGSGGSGSSASGGSTS), SpeI restriction site (residues TS), and 6x Histidine tag*.

c *mCherry(opt); yeast optimization begins at residue 21; CAI (codon adaptation index) value = 0.92*.

d Anti-GFP Nanobody; yeast optimized (CAI value = 0.84)

e Glutathione S-transferase (GST); sequence begins with “SPILGYW…” and ends with “…DLVPRGS.”

f *FLAG Epitope, Flexible linker (GSGAGGSPGGGSGGSGSSASGGSTS), and 3xHA (Human influenza hemagglutinin) tag (YPYDVPDYAGYPYDVPDYAGSYPYDVPDYACG)*.

g *Nuclear Localization Signal (SRADPKKKRKV) SV40 Large T-antigen*.

h *Nuclear Export Signal (LAKILGALDIN)*.

i *MYC Epitope (EQKLISEEDL)*.

j *Maltose Binding Protein (MBP); sequence begins with “KIEEGKL…” and ends with “…NSSSARL”. There is a XhoI (residues LE) restriction site preceding the stop codon*.

k *BirA(R118G); 35 kD DNA-binding biotin protein ligase in Escherichia coli*.

l *CAAX Box Motif (GSGGCCIIS) from yeast Ras2*.

m *SNAP Tag; 20 kDa mutant of the DNA repair protein O6-alkylguanine-DNA alkyltransferase*.

n *S. pombe HIS5 gene product; Imidazoleglycerol phosphate dehydratase HisB*.

o *mScarlet-WT(opt) (CAI value = 0.93)*.

p *eGFP(opt); yeast codon optimization begins after residue 9 (CAI = 0.92). Contains S65T, F64L, R88Q, and H239L*.

q *ymUkG1(opt) yeast codon bias (CAI = 0.92)*.

r *eGFP(opt)-Lact-C2; Bovine Lactadherin C2 Domain (158 residues) binds phosphatidylserine on the inner leaflet of the plasma membrane*.

### Strategy for marker-less N-terminal gene tagging using universal promoters

While previous studies have focused primarily on C-terminal tagging cassettes (Longtine et al., 1998; Janke et al., 2004; Lee et al., 2013) few groups have developed methodologies for appending genes at their N-termini (Gauss et al., 2005; Booher and Kaiser, 2008). Two technical obstacles are responsible for this bias toward C-terminal tags (and libraries). First, the requirement of a selectable marker to follow the integration event is easily added within the continuous sequence that can include the (C-terminal) tag of interest, a universal terminator element, and a self-contained drug-resistance or auxotrophic marker cassette (as described in Figure 1). This becomes more challenging when attempting to introduce an N-terminal tag *and* selectable marker. Attempts have been made to include the marker cassette upstream of the endogenous promoter; however, this requires re-engineering of the 5′ UTR and either replacement or cloning of each individual promoter to drive expression of the tagged gene(s). Second, even if a universal promoter (for general, low, or over-expression) is chosen, each tagged gene of interest must still have a unique set of oligonucleotides to deliver the integration cassette to the desired locus. While this issue can be circumvented when using an existing C-terminal tagged yeast library to generate a new tagged collection (illustrated in Figures 2, 3), no such N-terminally tagged set currently exists.

**Figure 4:**
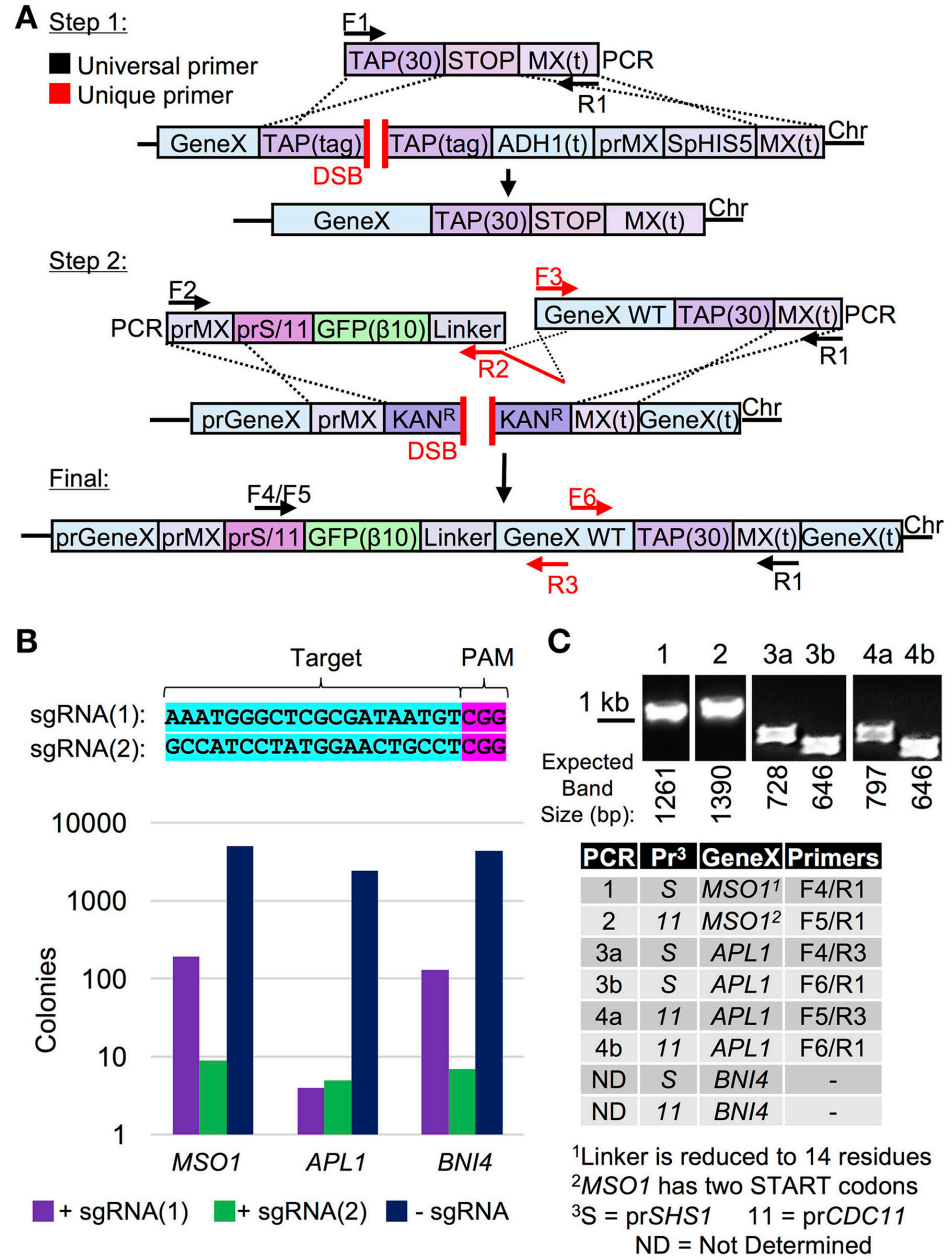
Cas9-based editing and yeast library conversion for a universal, N-terminal tagging strategy. **(A)** Step 1: the TAP collection was edited using Cas9 (pGF-V789), the sgRNA(TAP) (pGF-V799), and a donor PCR (F1/R1) [amplified from pGF-pUC57+TAP(30)-STOP-MX(t)] to remove the entire TAP cassette. Step 2: a N-terminal donor DNA cassette (left) was constructed including a tripartite split GFP β10 tag, pr*SHS1* or pr*CDC11* sequence, and a variable flexible linker (pGF-TOPO+IVL1334 or pGF-TOPO+IVL1335). Both (i) the N-terminal tag (F2, “prMX clone out F”/R2, “GFP(β10)-Link-*BNI4* R” as an example) and (ii) the entire ORF fused to the TAP(30)-MX(t) (F3, “*BNI4* clone out F”/R1) from chromosomal DNA (from strains obtained in Step 1) were PCR amplified. In some cases, large genes were amplified using overlapping PCR fragments. Finally, targeting of Cas9 to the Kan^R^ sequence (genome deletion collection) introduces a DSB; introduction of two (or more) amplified PCR fragments allow for assembly of the N-terminally tagged gene and repair across the break with no selection marker. **(B)** Two sgRNAs were created (Figure S3) to target Cas9 to the Kan^R^ gene (top). Yeast deleted for *MSO1, APL1*, or *BNI4* (GFY-2251, GFY-2259, and GFY-2256) and harboring the Cas9 vector (pGF-V789) were transformed with either of the two guide RNAs (pGF-425+IVL1274 or pGF-425+IVL1275) or an empty vector, selected on SD-URA-LEU, and the number of colonies was quantified (bottom). **(C)** The N-terminal tagging strategy **(A)** was performed for *MSO1, APL1*, and *BNI4* with the described PCR fragments and the Kan^R^ sgRNA(2) plasmid. Colonies obtained on SD-URA-LEU plates were tested for G418 resistance (*n* = 30–50) and between 95 and 100% of all colonies had lost a functioning Kan^R^ cassette. Clonal isolates (*n* = 1) sensitive to G418 were assayed by diagnostic PCRs (primer combinations as shown, Table S1) and DNA sequencing of the manipulated locus. The expected PCR fragment sizes are illustrated. Oligonucleotides used included those within the promoter (*SHS1*/*CDC11*), the gene of interest (F or R), and the MX(t). Additional loci tested can be found in Table S4.

Therefore, we have developed a general strategy using Cas9 that could be used to generate an N-terminal tagged allele that is (i) marker-less, (ii) under control of any chosen (one or more) sets of promoters, (iii) requires a minimum number of unique and/or extended (60 bp) oligonucleotides for cloning each separate gene, and (iv) builds upon two existing yeast library collections (Figure 4). To begin, a set of genes were chosen that exist in both the TAP tag and genome deletion libraries (one current limitation of this strategy as missing genes or essential genes would not be usable). Next, yeast were transformed with plasmid-borne Cas9 followed by co-transformation of the TAP-targeting sgRNA and a donor DNA fragment that removed the entire C-terminal tag and *HIS5* marker (Figure 4A). Second, chromosomal genomic DNA was used as the template to amplify the entire gene including the MX terminator sequence. A universal construct was also built that contained the MX promoter sequence, one of two chosen promoters, the GFP(β10) tag of the tripartite split GFP system (Cabantous et al., 2013; Finnigan et al., 2016) and a repetitive Ser-Gly-based flexible linker. This construct was used to generate an amplified fragment containing a single (unique) oligonucleotide tail that extended toward the N-terminus of the gene to be tagged (Figure 4A). This method required use of the genome deletion collection (each gene replaced with the MX Kan^R^ cassette) to provide a DSB over which the multiple PCR fragments would reassemble the N-terminally tagged gene. We tested two different sgRNAs to target the Kan^R^ open reading frame (Figure S3) and examined their ability to target three different loci (*MSO1, APL1*, and *BNI4*) (Figure 4B). The sgRNA(Kan-2) target sequence resulted in more efficient editing and was subsequently used for our N-terminal tagging protocol. The genome deletion strains harboring Cas9 were co-transformed with (i) the sgRNA(Kan-2) plasmid, (ii) the amplified donor PCR containing the N-terminal tag and one of two common promoters, and (iii) the entire amplified gene of interest from the converted TAP-MX(t) strains (from Step 1). Yeast were plated and selected on SD-URA-LEU, tested for the loss of the Kan^R^ cassette, and the entire ensemble was confirmed via diagnostic PCR and DNA sequencing (Figure 3C). Our initial analysis (*n* = 1 isolate for the *MSO1* and *APL1* strains) demonstrated this protocol does allow for the universal addition of tags at the N-terminus. While nearly 100% of surviving colonies had lost G418 resistance (our unpublished results), we observed several alterations in our DNA sequencing including a shortening of one of the repetitive Ser-Gly linkers and the addition of a second initiator Met for our *MSO1* assemblies. We expanded our initial set to include N-terminal tagging at 37 additional loci (Table S4). Across all 40 genomic targets, or screening methodology resulted in a 75% success rate (30/40) when assaying only a single isolate per event. One of the technical challenges we encountered was the use of a long flexible Ser-Gly rich linker sequence which contains repetitive DNA sequences that could provide a source of inappropriate cross over. While this methodology does not solve all existing challenges to the genome-wide construction of N-terminal tagged libraries, it does present a useful strategy for generating chromosomally integrated, expression-modulated, and marker-less sets with few required “unique” oligonucleotides–one of the major construction hurdles for generating large sets of strains.

### Using Cas9 to replace existing fluorescent markers

**Figure 5:**
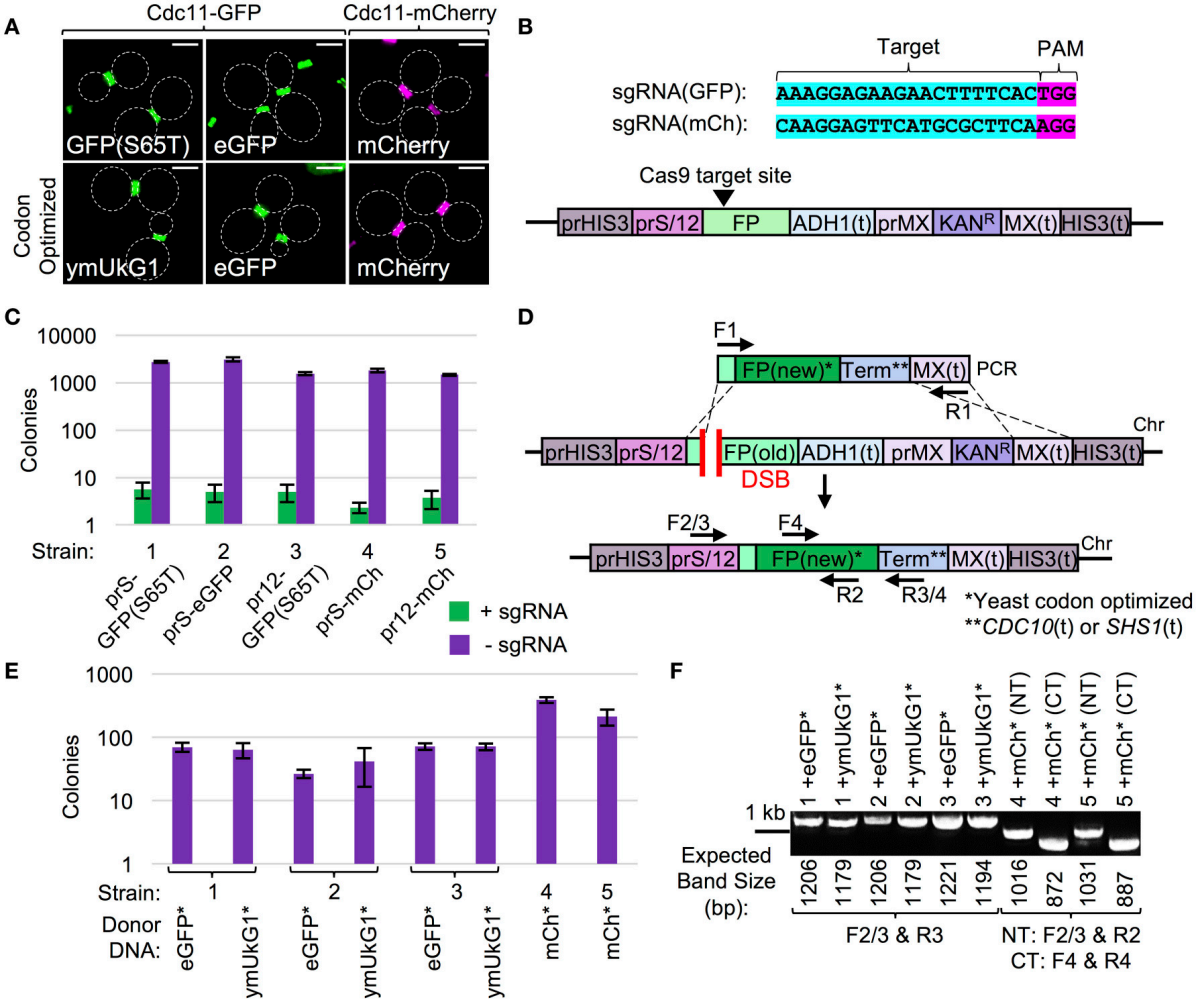
Cas9-based strategy for upgrading fluorescent markers. **(A)** Yeast (GFY-42 or GFY-330) were transformed with vectors (pGF-IVL1419 to pGF-IVL1424) expressing a fusion of the *CDC11* septin to one of six GFP or mCherry variants and imaged by fluorescence microscopy. White dotted lines, cell periphery. Scale bar, 3 μm. **(B)** Three FP genes (GFP(S65T), eGFP, or mCherry) were integrated at the *HIS3* locus under one of two promoters with a drug-resistance marker (bottom). Two sgRNA cassettes were built (top) to target Cas9 to a target sequence in GFP or mCherry (Figure S3). **(C)** Yeast strains GFY-2613, GFY-2615, GFY-2617, GFU-2621, and GFY-2622 (labeled 1–5) containing the Cas9 vector (pGF-V789) were transformed with either an empty (pRS425), sgRNA(GFP) (pGF-425+IVL1276), or sgRNA(mCherry) (pGF-425+IVL1277) plasmid, plated on SD-URA-LEU, and the number of colonies was quantified in triplicate. Error, SD. **(D)** A marker-less integration strategy to replace one FP with a different fluorescent variant and/or a codon optimized version of the same FP gene. Donor DNA included codon optimized eGFP (from pGF-IVL1251), coral ymUkG1 (pGF-IVL1253), or mCherry (pGF-IVL1255). A unique terminator sequence for each donor construct allowed HR to only occur within (i) common 30 bp upstream FP coding sequences and (ii) the MX(t). **(E)** Donor PCRs were amplified from **(D)** using universal primers (F1, “GFP clone out F”/“mCherry clone out F” and R1, “MX clone out R2”) and digested with *DpnI*. Equimolar amounts were co-transformed into yeast strains 1–5 **(C)** with the appropriate sgRNA vector, plated to SD-URA-LEU, and the colony count was quantified in triplicate. Error, SD. **(F)** Randomly selected isolates were tested for survival on G418 and yeast lacking the Kan^R^ cassette (*n* = 2) were assayed by both diagnostic PCR and DNA sequencing. For strains (1–3), PCRs (F2/3 and R3) utilized DNA primers to the promoter and newly introduced terminator sequences. For strains (4–5), two diagnostic PCRs (F2/3 and R2, F4, and R4) were performed to confirm proper integration (see Table S1). The expected fragment size (bp) is illustrated.

Given the wide-spread use of fluorescent protein (FP) tags in many areas of molecular and cellular biology coupled with the discovery and development of new variants, it is surprising that there is no current “upgrade” methodology described that allows for efficient switching between FPs. This is a major issue for both individual gene collections which have utilized one or more FPs (either plasmid- or chromosomally-based) or entire yeast libraries (e.g., GFP-tagged collection). Significant study has been invested in the engineering of new fluorescent variants to (i) include a wider visual spectrum including the near infrared, (ii) have modified properties such as maturation time, photostability, brightness, etc., or (iii) be utilized in bimolecular fluorescence complementation type assays (BiFC), such as FRET or split FP systems (Nagai et al., 2002; Rizzo et al., 2004; Shaner et al., 2004; Pedelacq et al., 2006; Filonov et al., 2011; Cabantous et al., 2013; Miller et al., 2015; Bindels et al., 2017). The development of technical hardware (e.g., microscopes and digital cameras), computer software, and the variations of usable proteins themselves has provided many new options, but no method exists to rapidly and efficiently convert one tagged collection into another (new) set. The traditional means of cloning a new FP set into a collection (or library) of yeast strains (or plasmids) still requires a significant number of unique oligonucleotides, molecular cloning, and faces the same technical issues for targeting previously C-terminally tagged strains (Figure 1). Moreover, while some of the newest FP variations require specialized microscopy components (allowing for maximal use of the excitation and/or emission spectra of the particular FPs) that might not be available for all users, a simple solution already exists for the conversion of older GFP/FP variants into more stable/readily expressed variants—alteration of codon bias (Kaishima et al., 2016). The initial discovery and implementation of FPs as well as the sharing of cloned materials across laboratories has often resulted in an apparent paradox—presence of the “upgraded” FP variant might suffer from a poor codon bias for particular organisms as the FP may have been evolved/developed for expression in a different model system. Indeed, previous work tested a set of GFP variants in a controlled setting (in budding yeast) varying only the codon bias—this resulted in dramatically improved overall expression and fluorescence intensities of the optimized FPs (Kaishima et al., 2016). Thus, we developed a variation of our Cas9-based methodology (Figures 2, 3) for upgrading of an existing FP-tagged gene (GFP or mCherry) to a newer version or the same version with an altered codon bias (Figure 5).

We examined six FP fusions to the yeast Cdc11 septin protein *in vivo* at the division site (bud neck) by fluorescent microscopy including GFP(S65T), eGFP, mCherry, codon-optimized versions of eGFP and mCherry, and the coral ymUkG1 FP variant (Figure 5A). We designed a series of tester strains with either GFP(S65T), eGFP, or mCherry placed at the same genomic position (*HIS3*), all containing the common MX Kan^R^ cassette to match many FP-tagged plasmids, integrated strains, and genome-wide collections (Figure 5B). Next, we selected two sgRNA sequences to target either GFP or mCherry at positions very close to the N-terminus of each FP, but only matching a sequence unique to the given fluorescent gene and not any cloned linker region (Figure S3). We tested these sgRNAs for their ability to recruit Cas9 to the intended site and induce a DSB by transforming our set of engineered yeast strains with plasmid-borne Cas9 followed by the sgRNA plasmid. As expected, we found that DSB formation resulted in >99% of yeast being inviable (Figure 5C). Importantly, expression of the incorrectly matching guide RNA to the FP strain (e.g., sgRNA(GFP) in the mCherry-yeast strain) did not cause any editing, demonstrating the chosen sgRNA target sequences are unique and specific to each FP gene (our unpublished results). Next, we developed a small set of donor DNA cassettes that would allow for efficient, marker-less replacement of the endogenous FP with the upgraded version(s) (Figure 5D, Table 2). These included the coral ymUkG1 (Tsutsui et al., 2008), eGFP (Cormack et al., 1996), and mCherry (Shaner et al., 2004)—all synthesized *de novo* with an optimized yeast codon bias. Expression of Cas9, coupled with co-transformation of the appropriate sgRNA and amplified donor DNA allowed for efficient generation of hundreds of colonies per integration event (Figure 5E). Following a pre-selection assay to confirm loss of the genomic marker (Kan^R^), proper editing was confirmed by both diagnostic PCR (Figure 5F) and DNA sequencing (*n* = 2 for each editing event by DNA sequencing). Our Cas9-based FP-swapping strategy is applicable to plasmid-driven or endogenously-tagged genes as well as entire libraries and should provide a useful means to merge many existing strains and collections with the rapidly evolving field of fluorescence protein biology and its many applications.

### Application of a Cas9-based gene drive as an alternative to synthetic genetic array (SGA)

**Figure 6:**
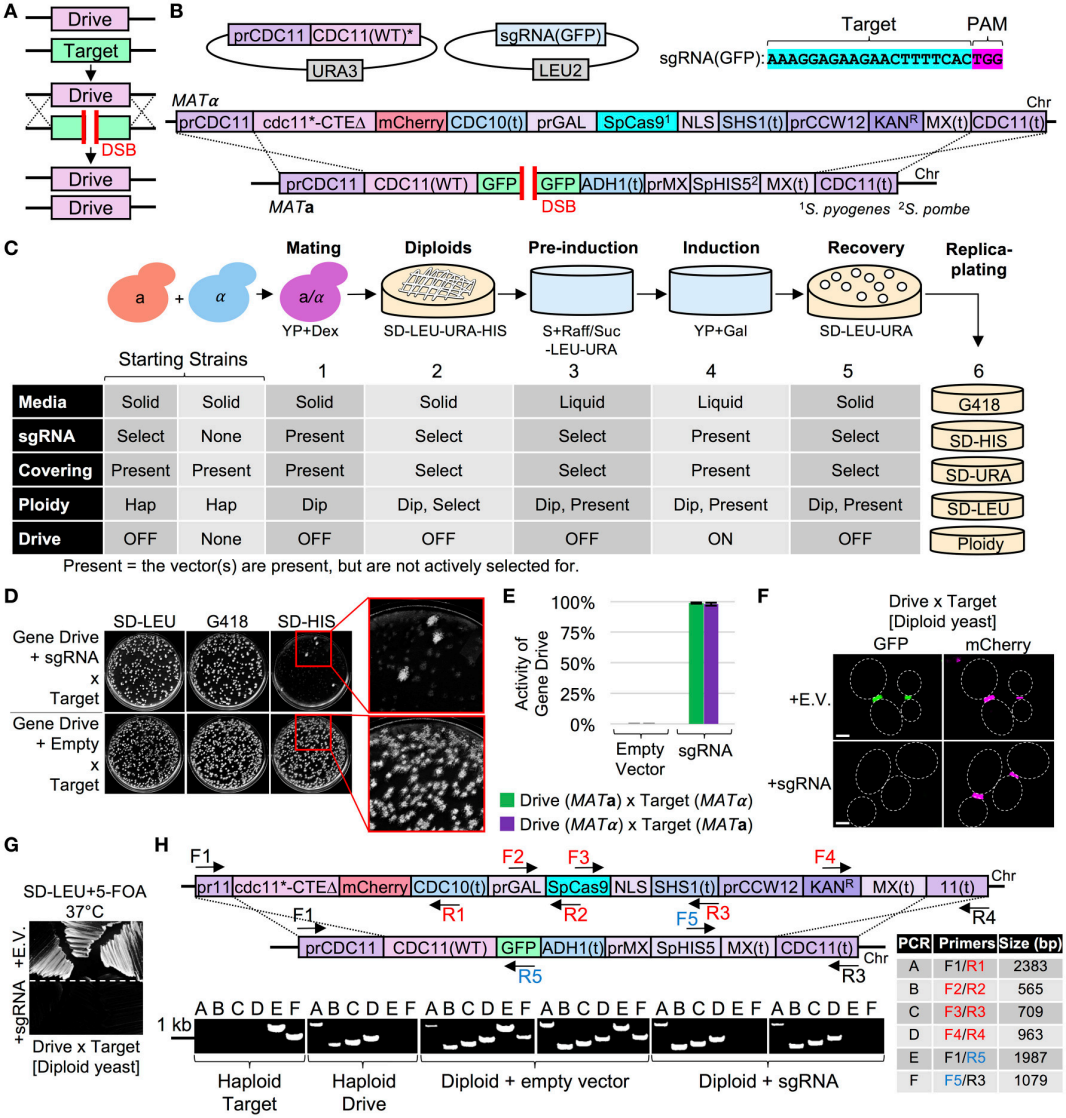
Use of a Cas9-based gene drive to deliver a recessive allele to a yeast library of the opposite mating type and convert to a homozygous diploid condition. **(A)** General strategy for a nuclease-based gene drive. The “drive” consists of Cas9 placed at (or replacing) an endogenous gene. When paired in a diploid cell, Cas9 is expressed and targeted by the sgRNA to the homologous WT gene creating a DSB. Alignment of the entire homologous chromosome serves as the source of donor DNA to copy the gene drive. **(B)** Design of a gene drive for a recessive allele of an essential gene (*CDC11*). A *URA3*-based covering vector (pGF-IVL1146) expressing a WT copy of *CDC11* is present in the starting gene drive strain. Cas9 is under control of the *GAL1/10* promoter and harbors a Kan^R^ marker; the entire array is integrated at the *CDC11* locus. A “target” strain (*CDC11*-GFP(S65T) fusion and the *SpHIS5* cassette) was generated. The sgRNA(GFP) plasmid (pGF-425+IVL1276) was transformed into the haploid gene drive strain (GFY-2442 or GFY-2440). **(C)** The status of each component (plasmids, ploidy, markers, drive activity, etc.) is listed for each step. For a detailed description of drive activation, see section Materials and Methods. **(D)** Assaying for marker status on G418 and SD-HIS media of active and non-active (empty vector) gene drive diploids. **(E)** Quantification of colonies from multiple diploid crosses (GFY-2624 × GFY-2440 and GFY-2625 × GFY-2442) in triplicate. The percentage of surviving colonies (*n* = 250–500) is illustrated as “drive activity.” Error, SD. **(F)** Diploids strains **(D)** were imaged by fluorescence microscopy. Dotted white lines, cell periphery. Scale bar, 3 μm. **(G)** Diploids **(D)** were selected on SD-LEU+5-FOA medium for 2 days at 37°C. **(H)** Diagnostic PCRs on diploids strains **(D)** (*n* = 2) and haploids strain controls (GFY-2624, “target” and GFY-2440, “drive”). Oligonucleotides (table) unique to the drive (red), target (blue), or both, (black) are shown (also see Table S1). Expected PCR sizes (bp) are illustrated.

Apart from our designed Cas9-based editing methods for tag replacement, we explored a powerful arrangement of the nuclease that could be applied to yeast library construction known as a “gene drive.” Briefly, this organization of Cas9 requires the nuclease gene to be present at a locus of interest (either replacing and deleting an endogenous gene, or positioned proximal to the native/modified gene) and a sgRNA that is designed to target the WT copy of the gene on the opposite chromosome in a diploid cell (Figure 6A). The presence and expression of both Cas9 and the sgRNA induces a DSB on the WT copy of the target gene; the source of donor DNA is the entire homologous chromosome containing Cas9 itself (and, possibly, the sgRNA-expressing cassette). The cell repairs the DSB by copying over the engineered locus and propagating the Cas9 gene within a diploid (Figure 6A). This technique is of particular interest to the fields of insect biology, pest control, and the prevention and eradication of insect-borne pathogens, such as malaria. Recent work in flies and mosquitos has demonstrated that the Cas9 gene drive has the potential to be used as a powerful biological agent for population control (Gantz et al., 2015; Hammond et al., 2016). However, no current studies have explored the use of a gene drive within basic research except a single publication piloting the use of a Cas9 drive in budding yeast but did not include any demonstration for strain generation (DiCarlo et al., 2015).

We envisioned that a gene drive might be repurposed in *S. cerevisiae* for the goal of strain construction. To date, two common methodologies are used in yeast for genome manipulation. The first, and by far, the most widely utilized, is simply transformation of amplified PCR donor DNA, coupled with selectable markers to insert, modify, and/or alter endogenous sequences based on homologous recombination. This process occurs in yeast even in the absence of a DSB (although the repair of DSBs on either plasmids or the genome is increased by many orders of magnitude compared to intact native sequence). Second, following construction of libraries or modified strains, combinations of alleles can be coupled together by different methods including (i) mating strains of the opposite type followed by diploid selection and sporulation or (ii) successive rounds of traditional transformation events. In response to the creation of many yeast libraries and the development of robotic and semi-autonomous methodologies for manipulating yeast strains, the “synthetic genetic array” (SGA) protocol was developed (Tong et al., 2001). Briefly, this method combines a query strain (harboring a desired allele or deletion of one gene) and combines it with an entire yeast library of choice (GFP, deletion collection, etc.) into a haploid strain in a high-throughput fashion. However, the “full” SGA protocol has some restrictions (both technical and genetic). First, a unique genetic background must be utilized to allow for subsequent haploid selection steps. Second, the entire protocol requires between 3 and 4 weeks for the procedure from start to finish, excluding the selection of clonal isolates. Third, the process requires additional exogenous components for ploidy selection and the use of selection markers. However, we envisioned that for some scenarios, combination of a query gene of interest and the modified library would *not* need to be assayed within a haploid cell, and, instead, a diploid state would be sufficient (e.g., only the first two steps of an SGA-type protocol). Examples would include over-expression arrays or FP-tagged libraries (GFP); in both cases, obtaining the haploid state may not be required nor cause a significant difference in the final assay. It is very likely that the query allele (commonly a hypomorph) of interest *must* be expressed as the only copy within a diploid; therefore, achieving the homozygous state may be essential. Of note, even recent genetic screens (Berry et al., 2016) run into this same issue—one of the two components of their GFP/anti-GFP nanobody tethering system is plasmid-expressed and thus has a WT endogenous copy still present within the genome (that may alter or confound the interpretation of results). The diploid selection method, as described by a SGA-type protocol, is unable to provide two copies of any query allele.

Therefore, we have provided a detailed account of the design of a common gene drive installation protocol and tested its effectiveness at generating the homozygous diploid state. To date, this “Super-Mendelian” arrangement is the only methodology that would be able to rapidly convert a heterozygote into a homozygous condition within an individual diploid genome. Furthermore, we have chosen an essential gene to illustrate the breadth of alleles that are possible within this same Cas9 arrangement. We generated a “cargo-based” gene drive and integrated the entire construct at the native *CDC11* locus (Figure 6B). The design of our constructed gene drive plasmid (Table 2) also allows for introduction of any other yeast gene in place of our chosen *CDC11* allele for immediate application. This strain harbored (i) a *URA3*-based covering vector to protect the strain from the loss-of-function *cdc11* allele we chose to include as the “cargo” and (ii) the sgRNA targeting the GFP sequence we previously designed (Figure 5). We have also included the (optional) selectable Kan^R^ marker to assay the presence of the drive. For biosecurity reasons, we have chosen to pilot application of this gene drive in a strain harboring *CDC11-GFP* at the native *CDC11* locus in strains of the opposite mating type that were also tagged with the *S. pombe HIS5* marker (for clarity in assaying “success” of the active drive). The procedure would be identical in all ways when/if the gene drive was targeted to the native *CDC11* gene (rather than the exogenous GFP sequence). We have illustrated the other modifications needed to target the native WT gene including alteration of the codon sequence of the putative Cas9 target site(s) within (i) the covering vector and (ii) the *cdc11* “cargo” allele (Figure 6B, Tables 1, 2).

Following design of the gene drive (created in both *MAT **a*** and *MAT***α** yeast), we have developed a protocol to allow for rapid activation of the drive followed by isolation of clonal diploid yeast colonies in only a few days (Figure 6C, Figures S5, S6). Activation of Cas9 (and the gene drive) resulted in >99% of all sampled cells (nearly 500 cells per plate) having lost the endogenous *CDC11-GFP-HIS5* target gene followed by full replacement of the gene drive itself (Figures 6D,E). Individual clonal isolates from both the active drive-containing strain and a control strain were tested for their ploidy status (haploid or diploid) and all sampled yeast were properly in the diploid state (Table S3). Compared to the starting haploid strains (Figure S6), diploid yeast harboring the empty vector and no sgRNA cassette expressed both the GFP and mCherry-tagged Cdc11 proteins (Figure 6F, top) whereas cells following activation of the gene drive had lost all Cdc11-GFP signal (Figure 6F, bottom). Moreover, loss of the *CDC11*-expressing *URA3*-marked covering vectors by growth on 5-FOA (Figure 6G) demonstrated successful action of the gene drive. Expression of the *cdc11-CTE*Δ*-mCherry* allele in a haploid cell as the only copy of *CDC11* renders yeast temperature sensitive at 37°C (Finnigan et al., 2015a). Finally, diploid genomes were isolated and examined by extensive diagnostic PCR illustrating the loss of target components and the copying of the entire drive to the homologous chromosome (*n* = 2 isolates for each diploid drive) (Figure 6H). This methodology could be coupled with several yeast library variants harboring (i) over-expression vectors, (ii) the GFP library, or (iii) other yeast collections that have been generated within either mating type. These results demonstrate the utility and success of a Cas9 gene drive for obtaining a homozygous diploid status for a desired allele—this strategy could be utilized by many model systems outside of yeast for bypassing time-consuming strain/organism construction steps (multiple generations, selection steps, etc.) by traditional methodologies.

## Discussion

### The marker-less option

The yeast research community has pioneered the use of genetic screens (forward and reverse) including the development of high-throughput automated SGA and high-content screening (HCS) technology. *S. cerevisiae* has been utilized to generate numerous types of genome-wide libraries and explore a range of biological questions including genetic interactions (Costanzo et al., 2010, 2016), protein-protein interactions (Tarassov et al., 2008; Sung et al., 2013), and transcriptional modulation (Mnaimneh et al., 2004; Rajkumar and Maerkl, 2012). However, the introduction of CRISPR/Cas9 in budding yeast has been met with a lukewarm reception. One possible reason is that the primary nuclease-dependent function of Cas9 is to generate targeted DSBs and allow for recombination into genomes of interest—something that budding yeast are capable of in the *absence* of any DSB. Indeed, we demonstrate in Figure 1 that traditional cloning in yeast can provide solutions to some of the issues surrounding strain construction. However, recent work has demonstrated that yeast can still benefit from use of CRISPR-based editing and, as we have shown, can be coupled with the genome-wide library infrastructure that has been available for nearly two decades to greatly aid in strain construction. Moreover, the use of Cas9 can provide a powerful selection tool—cell viability, that can be used for screening *sans* any selectable marker cassette.

It is surprising given the enormous amount of genome-wide (or sub-genome) yeast collections that exist and continue being generated that few groups have focused on library *conversion* rather than *de novo* development. There are still serious technical and costly challenges for building an entire tagged library (or large set of yeast strains in the many hundreds or thousands) including the cost of oligonucleotides (minimum of two extended DNA primers for each locus being targeted), high-throughput manipulation of many yeast strains (although this is an optional addition), and verification of the final manipulated genomes. Automation and the development of hardware that can aid in bulk transformations or mating events have addressed the logistical challenge of handling thousands of strains simultaneously (Tong et al., 2001; Liu et al., 2017). The major benefit of utilizing an existing library is that the placement of common sequences (e.g., C-terminal tag, terminator, selection marker cassette, etc.) has already been invested and verified. Thus, “universal” oligonucleotides can be used to amplify and target every modified locus within the collection. However, additional alterations must be considered when preparing the donor DNA cassette that would replace the existing tag/marker including the presence of common sequences that are often utilized as part of the MX drug/auxotrophic marker. Neglecting these sequences may, as we have demonstrated in this study, result in false positives during the marker “swap” integration event. Furthermore, the few published strategies to date for library conversion require the use of a (different) selectable marker to be inserted in place of the existing one or prevent unintended “swapping” between markers (Sung et al., 2008, 2013; Wosika et al., 2016).

The ability to have a “marker-less” genetic alteration is often desirable, but can also present technical challenges. Prior to the introduction of CRISPR/Cas9, there have been other systems used to remove a selectable marker (often in at least two or more consecutive steps) using integration vectors (Sikorski and Hieter, 1989), the Cre-Lox system (Germino et al., 2006), or other methods, such as the classic “loop in-loop out” (Landgraf et al., 2016; Wang et al., 2017). This is often extremely useful in strains where the presence of multiple plasmids and multiple loci have all been (each) marked with the entire ensemble of selectable markers in yeast (or if a particular genetic background does not include the full suite of auxotrophic knockouts). Our methodology provides a number of advances over current cloning systems. First, our developed system utilizes (i) the developed yeast libraries currently available and (ii) CRISPR/Cas9 editing to provide a single-step, seamless integration *sans* any selectable marker. The selection generated by introduction of the DSB (viability) is sufficient to obtain correct isolates from a very small sampling pool—further optimization and development could utilize our strategy for the development of entire libraries. Second, our methodology *could* be performed in conjunction with the traditional selectable markers should the need arise (following the genetic locus after sporulation, etc.). Third, our system allows for rapid removal of the CRISPR components—the *URA3*-based Cas9 can be counter-selected on 5-FOA, and the sgRNA is rapidly lost on a high-copy plasmid. While our experiments utilized the *URA3* and *LEU2* markers for harboring Cas9 and the guide RNA, other options exist should either of these genes be required in the selection process (e.g., cloning Cas9 to a different *CEN*-based vector or direct integration in the parental strain). Fourth, our strategy could be employed in strains that already contain numerous other genetic alterations (tags, FPs, selectable markers) with virtually no unintended HR events (which would not be the case for the majority of the other C-terminal tagging cassette strategies that currently exist which rely on selection cassettes). Fifth, one of the major advantages of using CRISPR/Cas9 is the ability to multiplex—editing numerous genes simultaneously with exacting precision. None of the previous tagging strategies allow for tagging of more than a single gene per transformation event. CRISPR/Cas9 has been shown to allow for targeting of many loci in yeast with great accuracy and precision in a single step (Ryan and Cate, 2014; Bao et al., 2015; Horwitz et al., 2015; Finnigan and Thorner, 2016). Likewise, our system is fully capable of using two or more sgRNAs to target more than one loci at a time for editing, tagging, or marker-removal. Finally, in scenarios requiring two or more of the same marker (e.g., Kan^R^) to be used to mark a large number of deletions or loci, growth selection would no longer serve as a convenient means to assess the presence (or absence) of a particular modification and would require genomic interrogation of some sort. The ability to have a suite of selectable markers to tag different loci is a convenient and powerful tool in yeast and other model systems. However, the ability to manipulate a genome in the absence of any marker (which can then be used for other loci, or plasmid selection) provides more utility in strain construction and can allow for the generation of more complicated strains as the need arises.

### An expanded molecular toolkit

Previous studies describing molecular cassettes for gene tagging narrow their focus to (i) either the N- or C-termini of a gene, (ii) the inclusion one or more options for growth selection, and (iii) provide a common theme for the type of gene fusion (e.g., biochemical epitopes, FPs, etc.). Here, we provide a diverse sampling of commonly used tags but also include cellular localization signals (CAAX box motif, NLS, NES, Lact-C2 domain) and fusions that could be used for cellular assays and future genetic screens (mutant BirA, SNAP tag, anti-GFP nanobody domain, tripartite split GFP system, and the *S. pombe HIS5* gene) (Table 3). This collection should provide the yeast community with an expanded set of options for construction of both individual strains as well as entire genome-wide libraries. For instance, the utility of the GFP/anti-GFP nanobody pairing has been previously demonstrated (Berry et al., 2016) yet no such nanobody-tagged collection exists (the authors utilized the GFP library and a plasmid-expressed nanobody-fusion query set). Our toolkit is by no means saturated and future iterations might also include additional localization signal motifs, epitope combinations, and the ever-expanding collection of genetically-encoded fluorescent proteins.

One unique feature of our design is the ability to subtly alter, or completely “upgrade” an existing C-terminal fluorescent marker (either within a single strain or within, say, the GFP-tagged collection) with a codon optimized version of either the same FP, or a new one. While new FP are being discovered and engineered that allow for new capabilities such as four-color imaging (Filonov et al., 2011; Lee et al., 2013), increased brightness (Bindels et al., 2017), BiFC (Cabantous et al., 2013), and other controlled functions such as degradation (Houser et al., 2012), there is a paucity of molecular tools to allow for rapid inclusion of these FPs into existing systems. For older libraries, such as the yeast GFP collection (Huh et al., 2003), or for cloned plasmids or yeast strains from just a few years ago, a modified FP fusion might be required for better detection, multi-color imaging, stability, or other engineered properties. Therefore, methods need to be developed to bridge the expanded FP toolkit with strains (or plasmids) that *already* contain a cloned FP (or tag). The idea of providing the yeast community with new FPs with expanded markers is not a new one. Indeed, numerous studies have already focused on newly engineered green and red (and other) FP variants for traditional HR-based integration and cloning into the yeast genome (Sheff and Thorn, 2004; Vorvis et al., 2008; Lee et al., 2013; Slubowski et al., 2015; Malcova et al., 2016). However, all of these previous studies do not address the major issue at hand—the enormous investment of having to generate *de novo* the desired gene fusions, collections, or library which comes with the same technical and logistical restrictions (oligonucleotide investment, presence of a selectable marker, and promiscuous integration into strains already containing (other) cassettes). Finally, the sharing of new FP materials across disciplines and model systems has led to situations where a newly developed fluorescent protein might be expressed with the wrong codon bias in a given species. Even though the cost and utility of gene synthesis is growing more manageable and mainstream, many researchers choose not to re-synthesize *de novo* the entire exogenous FP tag for optimal expression in their given species (even though this should now become standard practice).

Therefore, our methodology has demonstrated both concepts—codon optimization using the same FP, or insertion of a newer variant in place of an older one (ymUkG1 in place of GFP). Our Cas9-based editing—unlike other “standard” cassette-based strategies—can be applied to editing of the genome or editing of existing plasmid-borne constructs. Cas9 paired with the appropriate guide RNA would target, create a DSB within the (existing) FP tag, and, given the presence of the MX-based cassette on the plasmid (which, again is most common-place for construction using existing tagging strategies), would allow for repair and re-circularization of the plasmid with no added selectable marker. The ability to edit both the genome and plasmids is unique to our Cas9-based editing and would not be possible with any of dozens of other tagging methodologies that are purely intended for targeting of the genome. While we chose to limit our FP-swap within the same color spectrum (green to green, red to red), future iterations might consider altering any FP to any other FP to provide maximum choice in experimental design and application.

### Novel, simplified application of CRISPR/Cas9 editing in yeast

To date, the majority of CRISPR-based applications in yeast and other systems have accepted the “one genomic target, one sgRNA” strategy as dogma. However, several studies have begun to expand beyond the idea of using multiple sgRNAs to target multiple locations. This concept has been useful in targeting a nuclease dead (dCas9) variant to naturally-occurring repeated sequences (such as delta elements or telomeres) in living cells to recruit sufficient dCas9-FP fusions to be able to visualize nuclear structures in real time (Ma et al., 2015; Dreissig et al., 2017). Similarly, targeting of constructed gene fusions to commonly repeated sequences has been done to deliver entire libraries of gene fusions (to the same genomic position) in a high-throughput automated system (Si et al., 2017). However, the idea of using a *minimal* number of guide RNA constructs to target *many* loci has not been widely used (or used at all). Here, we have developed four critical sgRNA constructs to target universally-used sequences that are found in most engineered yeast strains from all laboratories, and several of the most utilized yeast libraries: GFP, mCherry, TAP, and the Kan^R^ deletion cassette. Using only these four sgRNA plasmids, one could target an extreme number of loci across library collections, SGA haploid sets (numbering in the millions), and, most importantly, individual laboratory strain collections.

We recognize that our C-terminal tagging strategy likely has the most utility for application within the yeast community; in contrast, our N-terminal tagging scheme still requires some additional gene-specific components. However, our pilot methodology has demonstrated an attempt to minimize gene-specific reagents (oligonucleotides) while still utilizing existing yeast strains. Three current limitations of our N-terminal system include (i) the required use of a “common” promoter to drive expression, (ii) the presence of the modified gene in both the TAP and deletion libraries (our current methodology must exclude all essential genes), and (iii) gene-specific reagents. However, our primary goal for this study was to demonstrate that use of Cas9 paired with a “universal” guide RNA could allow for a variety of cloning techniques aside from merely the traditional C-terminally tagging scheme. By coupling a pre-selection step (e.g., loss of the *S. pombe HIS5* marker) to assess *loss* of the endogenous marker and sampling a single isolate, we have demonstrated a high success rate (100%) for our FP swapping or C-terminal tagging strategies. However, given testing at 40 separate genomic loci, our N-terminal tagging method still achieved a modest 75% success rate. Based on these results, we propose that application of our CRISPR-UnLOCK strategy include the pre-selection step on a small number (3–4 colonies) to ensure targeting of the correct locus. From this subset, we envision that verification of engineered strains also only require 1 or 2 clonal isolates for further testing. Clearly, the construction of genome-wide sets of strains is an ambitious endeavor and many yeast libraries have encountered difficulty in targeting particular loci and as a result, do not contain the full complement of non-essential genes. We envision the greatest utility of our method to the construction of individual yeast strains (rather than full libraries) with an emphasis on scenarios requiring marker-less tagging or complicated strains where traditional methods have too many technical restrictions.

One of the widely recognized limiting steps in performing CRISPR-based editing is the construction of the particular sgRNA-expressing cassette since the same Cas9 nuclease is commonly used, and, in many cases (mostly outside of yeast), no donor DNA is required. Therefore, we have approached the issue by targeting Cas9 to multiple *universal* sequences that populate most yeast strains today—this sets our CRISPR methodology apart from all other studies using this editing technology that are limited to the “one sgRNA, one target” mechanism. For those few studies that utilize natively repeating sequences (such as delta elements) within the genome, they are not able to tag or target endogenous genes at other loci. Thus, our study provides the first widely applicable, practical use of Cas9-editing to manipulate *existing* yeast strains (individual collections or libraries). This combines the power of CRISPR with the most commonly used markers and tags already present in nearly every lab strain published in the last two decades. Our CRISPR system does not replace traditional HR-mediated targeting and integration, but provides a diverse set of options for future strain development and greatly complements existing methodologies including SGA. Previously, we demonstrated the use of artificial 23 bp DNA sequences, installed within the yeast genome, to multiplex and recruit Cas9 using only a single sgRNA construct (Finnigan and Thorner, 2016). These non-traditional Cas9 targeting events to universal (or artificial) DNA sequences could easily be adopted and applied by other fields outside of budding yeast. We will continue to expand the utility of this toolkit by including other commonly used sequences (from auxotrophic markers, selection cassettes, and other widely used tags) to provide more strain development choices to the yeast community.

### CRISPR-based gene drives can be used in strain generation

We have included the use of a gene drive to illustrate a non-traditional application of the CRISPR editing technology that can also be combined with yeast library sets. While most high-throughput applications rely on the SGA protocol to combine alleles of interest and, ultimately, achieve a final haploid state (removing both WT copies of the two genes), some assays can simplify this process by utilizing a diploid yeast strain (e.g., halting the SGA protocol after diploid generation). For over-expression libraries (plasmid-borne), the contribution of the native WT gene is often inconsequential. Moreover, for use of the GFP-tagged collection to visualize localization of a gene product, a second unlabeled WT copy of the protein may reduce the overall fluorescence intensity (since only half of the protein would be fused to GFP), but would not alter the overall localization pattern. In these cases, the major technical hurdle remaining is achieving a homozygous diploid state for the desired *query* allele—the contribution of a second WT copy could drastically alter or mask the phenotype(s) of the final strain since most queries would likely be hypomorphs. Therefore, we piloted the use of a gene drive to carry out this specific function. Indeed, our described use of a gene drive is the first to achieve a homozygous state in a diploid for strain generation. The major difference between traditional transformation-based manipulation of each strain (individually) and an “automatic” active gene drive would be that no further yeast transformations would be required—all components for copying the gene drive and the proximal “cargo” allele of choice would be inherent to the initial starting strain. Furthermore, we have demonstrated the system is extremely robust and efficient with >99% of diploid cells being converted to the homozygous condition in 24 h following active expression of Cas9 and verified by four independent assays. By comparison, the (full) SGA protocol requires many weeks of sporulation and subsequent selection steps to achieve the desired haploid state. We envision that application of this gene drive strategy within a diploid strain could be very easily and rapidly performed with a library of choice (construction of the query strain would be the limiting step).

We have taken great care to keep our Cas9 gene drive system self-contained and controlled as we recognize that even in budding yeast, this particular CRISPR arrangement has the potential for unintended release or action. We have detailed numerous safeguards that have been imposed on our experimental design and execution (see Materials and Methods) with the most significant being use of an “artificial” target strain containing GFP at the *CDC11* locus (rather than an unmodified WT allele). We are confident that as additional arrangements and safeguards (DiCarlo et al., 2015) are investigated and explored, the use of gene drives within the laboratory setting (and elsewhere) might be seriously considered for strain generation, library construction, or ecological control in a manner that is ethically and logistically safe and regulated.

**Figure 7:**
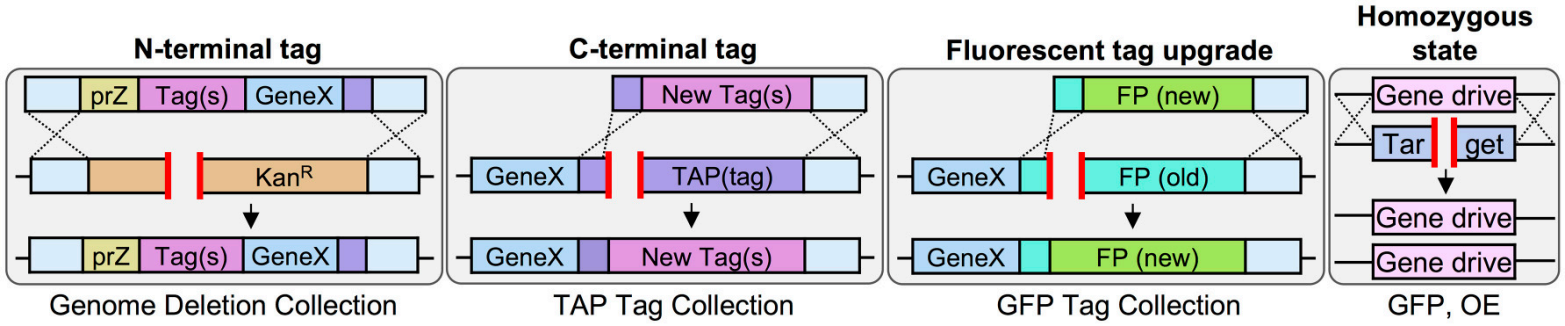
Summary of four CRISPR/Cas9-dependent methodologies for gene editing in yeast. The use of Cas9 editing (DSBs illustrated) for introduction of new markers into various yeast libraries sets. N-terminal tagging utilizes the TAP tag and deletion collections; C-terminal tagging utilizes the TAP collection, and the GFP library can be targeted to upgrade FPs. A Cas9 gene drive arrangement allows for rapid generation of a homozygous diploid state for a query allele including essential genes.

These CRISPR-based methodologies for construction of *S. cerevisiae* strains (Figure 7) could be applicable to other model systems and hopefully aid in the development of new techniques. Targeting of Cas9 to universal sites within a previously generated library or organism for tagging, upgrading or replacement of existing FPs, use of marker-less genomic integration, and the efficiency of a “gene drive” arrangement to achieve a homozygous diploid state for a query allele are all strategies that could be applied to many other biological systems.

## Author contributions

ER, RG, EW, ME, ET, MS, LA, TJ, SS, GB, CW, MH, and GF performed experiments and data analysis. ER, RG, EW, and ME generated figures, graphs, tables, provided references, and aided in writing and editing of the manuscript. GF performed data analysis and wrote the manuscript.

### Conflict of interest statement

The authors declare that the research was conducted in the absence of any commercial or financial relationships that could be construed as a potential conflict of interest.

## Abbreviations

5-FOA: 5-Fluoroorotic Acid
NLS: nuclear localization signal
NES: nuclear export signal
GFP: green fluorescent protein
eGFP: enhanced GFP
mCherry: monomeric red fluorescence protein variant
pr: promoter
t: terminator
bp: base pairs
sgRNA: single guide RNA
HR: homologous recombination
DSB: double-stranded break
HDR: homology-directed repair
NHEJ: non-homologous end joining
prMX/MX(t): promoter and terminator sequences for the *Ashbya gossypii* TEF1α (translation elongation factor 1α) used in the MX-based yeast cassettes
oligo: DNA oligonucleotide primer
CRISPR: clustered regularly interspaced short palindromic repeats
PAM: protospacer adjacent motif
TAP-tag: tandem affinity purification epitope tag
KAN/KAN^R^: Kanamycin resistance gene conferring yeast resistance to G418 disulfate
DIC: differential interference contrast light microscopy
Chr: chromosome
indel: insertion/deletion
FP: fluorescent protein
SGA: synthetic genetic array—use of this term specifically refers to the entire procedure (mating, diploid selection, sporulation and selection of desired haploid isolates)
HCS: high-content screening
OE: over-expression
BiFC: Bimoleclar fluorescence complementation.
