# Supplementary material for: CRISPR-UnLOCK: Multipurpose Cas9-Based Strategies for Conversion of Yeast Libraries and Strains
Source: Front Microbiol. 2017 Sep 20;8:1773. doi: 10.3389/fmicb.2017.01773 (PMC5611381; doi:10.3389/fmicb.2017.01773)
Supplement: Supplementary file 1 [file DataSheet1.DOCX]

**SUPPORTING INFORMATION**

For

**CRISPR-UnLOCKs: Multipurpose Cas9-Based Strategies for Conversion of Yeast Libraries and Strains**

Emily Roggenkamp,^1†^ Rachael M. Giersch,^1†^ Emily Wedeman,^1†^ Muriel Eaton,^1,2†^ Emily Turnqust,^1^ Madison N. Schrock,^1^ Linah Alkotami^1^, Thitikan Jirakittisonthon^3^, Samantha E. Schluter-Pascua^4^, Gareth H. Bayne^1^, Cory Wasko^1^, Megan Halloran,^1^ and Gregory C. Finnigan^1*^

^1^Department of Biochemistry and Molecular Biophysics, Kansas State University,

Manhattan, KS 66506 USA

^2^Current Address: Department of Medicinal Chemistry & Molecular Pharmacology, Purdue University, 575 Stadium Mall Dr., West Lafayette, IN 47907 USA

^3^Department of Anatomy and Physiology, College of Veterinary Medicine, Kansas State University, Manhattan, KS 66506 USA

^4^Department of Biology, Kansas State University, Manhattan, KS 66506 USA

^†^Authors contributed equally

^*^Correspondence to: Gregory C. Finnigan, Phone: (785) 532-6939; FAX; (785) 532-7278; E-mail: gfinnigan@ksu.edu

**Supplemental Figure 1:** sgRNA plasmid construction in yeast using *in vivo* ligation and homologous recombination.

The yeast 2µ-vector expressing the sgRNA was constructed using *in vivo* ligation (IVL) and homologous recombination (Finnigan and Thorner 2015). First, the plasmid pGF-V209 (pRS315; *LEU2*) was gapped by digesting with a downstream *SpeI* restriction site and repaired by IVL using a series of amplified PCR fragments. (i) the *SNR52* promoter (Pol III) amplified using extended oligonucleotides to link to the *prCDC12* on the gapped vector (template pGF-V796), and the chosen 20 base pair target sequence along with additional bases that extend into the tracrRNA gene sequence. (ii) the tracrRNA sequence along with the *SUP4* terminator sequence linked to the MX promoter from the standard drug resistance cassette assembly (Goldstein and McCusker 1999) was amplified with the 20 base pair target sequence along with several bases of upstream prSNR52 extending off the 5’ end (from template pGF-V814). (iii) The Hyg^R^ cassette (Goldstein and McCusker 1999) was amplified with an extended oligonucleotide tail to link to the downstream vector sequence on pRS315. Yeast were selected on YPD+Hygromycin plates, the assembled plasmids were isolated, transformed into *E. coli*, and confirmed by diagnostic PCR and Sanger DNA sequencing. CEN-based vectors were used for IVL rather than 2µ vectors. Second, following confirmation of the assembled sequence (top), the sgRNA cassette (without the *prCDC12* and Hyg^R^ sequences) was amplified by PCR including unique *BamHI* and *XhoI* restriction sites flanking the cassette and cloned into the TOPO II vector (Invitrogen). Third, the sgRNA sequence was subcloned to the high-copy yeast plasmid (pRS425; *LEU2*) by either the flanking *BamHI/XhoI* sites or the *NotI/SpeI* sites (present in the TOPO II vector). An intermediary step (subcloning to the bacterial TOPO II vector) was performed because the drug resistance markers for both pRS315 and pRS425 are Amp^R^ whereas the TOPO II plasmid contains Kan^R^ (greatly increasing the efficiency of obtaining proper isolates).

**Supplemental Figure 2:** Plasmid maps for sgRNA cassettes, *S. pyogenes* Cas9-expressing plasmid, TAP-STOP-MX(term) cassette, N-terminal GFP(β10) tagging cassettes, and 18 C-terminal tagging cassettes.

**pRS425 + prSNR52::sgRNA(TAP)::SUP4(term)**

pGF-V799

catgattacgccaagcgcgcaattaaccctcactaaagggaacaaaagctggagctccaccgcggtggcggccgctctagaactagt**GGATCC**tcactaa**agggaacaaaagctggagct**tctttgaaaagataatgtatgattatgctttcactcatatttatacagaaacttgatgttttctttcgagtatatacaaggtgattacatgtacgtttgaagtacaactctagattttgtagtgccctcttgggctagcggtaaaggtgcgcattttttcacaccctacaatgttctgttcaaaagattttggtcaaacgctgtagaagtgaaagttggtgcgcatgtttcggcgttcgaaacttctccgcagtgaaagataaatgatc**TAAGAAAATCTCATCCTCCG**GTTTTAGAGCTAGAAATAGCAAGTTAAAATAAGGCTAGTCCGTTATCAACTTGAAAAAGTGGCACCGAGTCGGTGGTGC**TTTTTTTGTTTTTTATGTCTtcgagtcatgtaattagtta**tgtcacgc**CTCGAG**ggggggcccggtacccaattcgccctatagtgagtcgtattacgcgcgctcactggccgtcgttttacaacgtcgtgactgggaaaaccctggcgttacccaacttaatcgccttgc

**pRS425 + prSNR52::sgRNA(Kan v1.0)::SUP4(term)**

pGF-425+1274

ggctttacactttatgcttccggctcctatgttgtgtggaattgtgagcggataacaatttcacacaggaaacagctatgaccatgattacgccaagcgcgcaattaaccctcactaaagggaacaaaagctggagctccaccgcggtg**GCGGCCGC**CAGTGTGATGGATATCTGCAGAATTCGCCCTT**GGATCC**tcactaa**agggaacaaaagctggagct**tctttgaaaagataatgtatgattatgctttcactcatatttatacagaaacttgatgttttctttcgagtatatacaaggtgattacatgtacgtttgaagtacaactctagattttgtagtgccctcttgggctagcggtaaaggtgcgcattttttcacaccctacaatgttctgttcaaaagattttggtcaaacgctgtagaagtgaaagttggtgcgcatgtttcggcgttcgaaacttctccgcagtgaaagataaatgatc**AAATGGGCTCGCGATAATGT**GTTTTAGAGCTAGAAATAGCAAGTTAAAATAAGGCTAGTCCGTTATCAACTTGAAAAAGTGGCACCGAGTCGGTGGTGC**TTTTTTTGTTTTTTATGTCTtcgagtcatgtaattagtta**tgtcacgc**CTCGAG**AAGGGCGAATTCCAGCACACTGGCGGCCGTT**ACTAGT**ggatcccccgggctgcaggaattcgatatcaagcttatcgataccgtcgacctcgagggg

**pRS425 + prSNR52::sgRNA(Kan v2.0)::SUP4(term)**

pGF-425+1275

gctttacactttatgcttccggctcctatgttgtgtggaattgtgagcggataacaatttcacacaggaaacagctatgaccatgattacgccaagcgcgcaattaaccctcactaaagggaacaaaagctggagctccaccgcggtg**GCGGCCGC**CAGTGTGATGGATATCTGCAGAATTCGCCCTT**GGATCC**tcactaa**agggaacaaaagctggagct**tctttgaaaagataatgtatgattatgctttcactcatatttatacagaaacttgatgttttctttcgagtatatacaaggtgattacatgtacgtttgaagtacaactctagattttgtagtgccctcttgggctagcggtaaaggtgcgcattttttcacaccctacaatgttctgttcaaaagattttggtcaaacgctgtagaagtgaaagttggtgcgcatgtttcggcgttcgaaacttctccgcagtgaaagataaatgatc**GCCATCCTATGGAACTGCCT**GTTTTAGAGCTAGAAATAGCAAGTTAAAATAAGGCTAGTCCGTTATCAACTTGAAAAAGTGGCACCGAGTCGGTGGTGC**TTTTTTTGTTTTTTATGTCTtcgagtcatgtaattagtta**tgtcacgc**CTCGAG**AAGGGCGAATTCCAGCACACTGGCGGCCGTT**ACTAGT**ggatcccccgggctgcaggaattcgatatcaagcttatcgataccgtcgacctcgaggg

**pRS425 + prSNR52::sgRNA(GFP)::SUP4(term)**

pGF-425+1276

ttacactttatgcttccggctcctatgttgtgtggaattgtgagcggataacaatttcacacaggaaacagctatgaccatgattacgccaagcgcgcaattaaccctcactaaagggaacaaaagctggagctccaccgcggtg**GCGGCCGC**CAGTGTGATGGATATCTGCAGAATTCGCCCTTGGATCCtcactaa**agggaacaaaagctggagct**tctttgaaaagataatgtatgattatgctttcactcatatttatacagaaacttgatgttttctttcgagtatatacaaggtgattacatgtacgtttgaagtacaactctagattttgtagtgccctcttgggctagcggtaaaggtgcgcattttttcacaccctacaatgttctgttcaaaagattttggtcaaacgctgtagaagtgaaagttggtgcgcatgtttcggcgttcgaaacttctccgcagtgaaagataaatgatc**aaaggagaagaacttttcac**GTTTTAGAGCTAGAAATAGCAAGTTAAAATAAGGCTAGTCCGTTATCAACTTGAAAAAGTGGCACCGAGTCGGTGGTGC**TTTTTTTGTTTTTTATGTCTtcgagtcatgtaattagtta**tgtcacgcCTCGAGAAGGGCGAATTCCAGCACACTGGCGGCCGTT**ACTAGT**ggatcccccgggctgcaggaattcgatatcaagcttatcgataccgtcgacctcgagggggggc

**pRS425 + prSNR52::sgRNA(mCherry)::SUP4(term)**

pGF-425+1277

ccccaggctttacactttatgcttccggctcctatgttgtgtggaattgtgagcggataacaatttcacacaggaaacagctatgaccatgattacgccaagcgcgcaattaaccctcactaaagggaacaaaagctggagctccaccgcggtg**GCGGCCGC**CAGTGTGATGGATATCTGCAGAATTCGCCCTTGGATCCtcactaa**agggaacaaaagctggagct**tctttgaaaagataatgtatgattatgctttcactcatatttatacagaaacttgatgttttctttcgagtatatacaaggtgattacatgtacgtttgaagtacaactctagattttgtagtgccctcttgggctagcggtaaaggtgcgcattttttcacaccctacaatgttctgttcaaaagattttggtcaaacgctgtagaagtgaaagttggtgcgcatgtttcggcgttcgaaacttctccgcagtgaaagataaatgatc**caaggagttcatgcgcttca**GTTTTAGAGCTAGAAATAGCAAGTTAAAATAAGGCTAGTCCGTTATCAACTTGAAAAAGTGGCACCGAGTCGGTGGTGC**TTTTTTTGTTTTTTATGTCTtcgagtcatgtaattagtta**tgtcacgcCTCGAGAAGGGCGAATTCCAGCACACTGGCGGCCGTT**ACTAGT**ggatcccccgggctgcaggaattcgatatcaagcttatcgataccgtcgacctcgagggggggcccggtacccaattcgccctatagtgagtcgtattacgcgcgctcactggccgtcgttttacaacgtcgtgactgggaa

**pRS316 + prGAL1/10::SpCas9-NLS::CDC10-3’UTR**

pGF-V789

gtgcgggcctcttcgctattacgccagctggcgaaggggggatgtgctgcaaggcgattaagttgggtaacgccagggttttcccagtcacgacgttgtaaaacgacggccagtgaattgtaatacgactcactatagggcgaattggagctccaccgcggtg**gcggccgc**CAGTGTGATGGATATCTGCAGAATTCAGGCCTGAATTCGCCCTT**GACAGGTTATCAGCAACAACACAGTCATATCCATTCTCAATTAGCTCTACCACAGTGTGTGAACCAATGTATCCAGCACCACCTGTAACCAAAACAaTTTTAGAAGTACTTTCACTTTGTAACTGAGCTGTCATTTATATTGAATTTTCAAAAATTCTTACTTTTTTTTTGGATGGACGCAAAGAAGTTTAATAATCATATTACATGGCATTACCACCATATACATATCCATATACATATCCATATCTAATCTTACTTATATGTTGTGGAAATGTAAAGAGCCCCATTATCTTAGCCTAAAAAAACCTTCTCTTTGGAACTTTCAGTAATACGCTTAACTGCTCATTGCTATATTGAAGTACGGATTAGAAGCCGCCGAGCGGGTGACAGCCCTCCGAAGGAAGACTCTCCTCCGTGCGTCCTCGTCTTCACCGGTCGCGTTCCTGAAACGCAGATGTGCCTCGCGCCGCACTGCTCCGAACAATAAAGATTCTACAATACTAGCTTTTATGGTTATGAAGAGGAAAAATTGGCAGTAACCTGGCCCCACAAACCTTCAAATGAACGAATCAAATTAACAACCATAGGATGATAATGCGATTAGTTTTTTAGCCTTATTTCTGGGGTAATTAATCAGCGAAGCGATGATTTTTGATCTATTAACAGATATATAAATGCAAAAACTGCATAACCACTTTAACTAATACTTTCAACATTTTCGGTTTGTATTACTTCTTATTCAAATGTAATAAAAGTATCAACAAAAAATTGTTAATATACCTCTATACTTTAACGTCAAGGAGAAAAAACTATAATGGACAAGAAGTACTCCATTGGGCTCGATATCGGCACAAACAGCGTCGGtTGGGCCGTCATTACGGACGAGTACAAGGTGCCGAGCAAAAAATTCAAAGTTCTGGGCAATACCGATCGCCACAGCATAAAGAAGAACCTCATTGGCGCCCTCCTGTTCGACTCCGGGGAGACGGCCGAAGCCACGCGGCTCAAAAGAACAGCACGGCGCAGATATACCCGCAGAAAGAATCGGATCTGCTACCTGCAGGAGATCTTTAGTAATGAGATGGCTAAGGTGGATGACTCTTTCTTCCATAGGCTGGAGGAGTCCTTTTTGGTGGAGGAGGATAAAAAGCACGAGCGCCACCCAATCTTTGGCAATATCGTGGACGAGGTGGCGTACCATGAAAAGTACCCAACCATATATCATCTGAGGAAGAAGCTTGTAGACAGTACTGATAAGGCTGACTTGCGGTTGATCTATCTCGCGCTGGCGCATATGATCAAATTTCGGGGACACTTCCTCATCGAGGGGGACCTGAACCCAGACAACAGCGATGTCGACAAACTCTTTATCCAACTGGTTCAGACTTACAATCAGCTTTTCGAAGAGAACCCGATCAACGCATCCGGAGTTGACGCCAAAGCAATCCTGAGCGCTAGGCTGTCCAAATCCCGGCGGCTCGAAAACCTCATCGCACAGCTCCCTGGGGAGAAGAAGAACGGCCTGTTTGGTAATCTTATCGCCCTGTCACTCGGGCTGACCCCCAACTTTAAATCTAACTTCGACCTGGCCGAAGATGCCAAGCTTCAACTGAGCAAAGACACCTACGATGATGATCTCGACAATCTGCTGGCCCAGATCGGCGACCAGTACGCAGACCTTTTTTTGGCGGCAAAGAACCTGTCAGACGCCATTCTGCTGAGTGATATTCTGCGAGTGAACACGGAGATCACCAAAGCTCCGCTGAGCGCTAGTATGATCAAGCGCTATGATGAGCACCACCAAGACTTGACTTTGCTGAAGGCCCTTGTCAGACAGCAACTGCCTGAGAAGTACAAGGAAATTTTCTTCGATCAGTCTAAAAATGGCTACGCCGGATACATTGACGGCGGAGCAAGCCAGGAGGAATTTTACAAATTTATTAAGCCCATCTTGGAAAAAATGGACGGCACCGAGGAGCTGCTGGTAAAGCTTAACAGAGAAGATCTGTTGCGCAAACAGCGCACTTTCGACAATGGAAGCATCCCCCACCAGATTCACCTGGGCGAACTGCACGCTATCCTCAGGCGGCAAGAGGATTTCTACCCCTTTTTGAAAGATAACAGGGAAAAGATTGAGAAAATCCTCACATTTCGGATACCCTACTATGTAGGCCCCCTCGCCCGGGGAAATTCCAGATTCGCGTGGATGACTCGCAAATCAGAAGAGACCATCACTCCCTGGAACTTCGAGGAAGTCGTGGATAAGGGGGCCTCTGCCCAGTCCTTCATCGAAAGGATGACTAACTTTGATAAAAATCTGCCTAACGAAAAGGTGCTTCCTAAACACTCTCTGCTGTACGAGTACTTCACAGTTTATAACGAGCTCACCAAGGTCAAATACGTCACAGAAGGGATGAGAAAGCCAGCATTCCTGTCTGGAGAGCAGAAGAAAGCTATCGTGGACCTCCTCTTCAAGACGAACCGGAAAGTTACCGTGAAACAGCTCAAAGAAGACTATTTCAAAAAGATTGAATGTTTCGACTCTGTTGAAATCAGCGGAGTGGAGGATCGCTTCAACGCATCCCTGGGAACGTATCACGATCTCCTGAAAATCATTAAAGACAAGGACTTCCTGGACAATGAGGAGAACGAGGACATTCTTGAGGACATTGTCCTCACCCTTACGTTGTTTGAAGATAGGGAGATGATTGAAGAACGCTTGAAAACTTACGCTCATCTCTTCGACGACAAAGTCATGAAACAGCTCAAGAGGCGCCGATATACAGGATGGGGGCGGCTGTCAAGAAAACTGATCAATGGGATCCGAGACAAGCAGAGTGGAAAGACAATCCTGGATTTTCTTAAGTCCGATGGATTTGCCAACCGGAACTTCATGCAGTTGATCCATGATGACTCTCTCACCTTTAAGGAGGACATCCAGAAAGCACAAGTTTCTGGCCAGGGGGACAGTCTTCACGAGCACATCGCTAATCTTGCAGGTAGCCCAGCTATCAAAAAGGGAATACTGCAGACCGTTAAGGTCGTGGATGAACTCGTCAAAGTAATGGGAAGGCATAAGCCCGAGAATATCGTTATCGAGATGGCCCGAGAGAACCAAACTACCCAGAAGGGACAGAAGAACAGTAGGGAAAGGATGAAGAGGATTGAAGAGGGTATAAAAGAACTGGGGTCCCAAATCCTTAAGGAACACCCAGTTGAAAACACCCAGCTTCAGAATGAGAAGCTCTACCTGTACTACCTGCAGAACGGCAGGGACATGTACGTGGATCAGGAACTGGACATCAATCGGCTCTCCGACTACGACGTGGATCATATCGTGCCCCAGTCTTTTCTCAAAGATGATTCTATTGATAATAAAGTGTTGACAAGATCCGATAAAAATAGAGGGAAGAGTGATAACGTCCCCTCAGAAGAAGTTGTCAAGAAAATGAAAAATTATTGGCGGCAGCTGCTGAACGCCAAACTGATCACACAACGGAAGTTCGATAATCTGACTAAGGCTGAACGAGGTGGCCTGTCTGAGTTGGATAAAGCCGGCTTCATCAAAAGGCAGCTTGTTGAGACACGCCAGATCACCAAGCACGTGGCCCAAATTCTCGATTCACGCATGAACACCAAGTACGATGAAAATGACAAACTGATTCGAGAGGTGAAAGTTATTACTCTGAAGTCTAAGCTGGTCTCAGATTTCAGAAAGGACTTTCAGTTTTATAAGGTGAGAGAGATCAACAATTACCACCATGCGCATGATGCCTACCTGAATGCAGTGGTAGGCACTGCACTTATCAAAAAATATCCCAAGCTTGAATCTGAATTTGTTTACGGAGACTATAAAGTGTACGATGTTAGGAAAATGATCGCAAAGTCTGAGCAGGAAATAGGCAAGGCCACCGCTAAGTACTTCTTTTACAGCAATATTATGAATTTTTTCAAGACCGAGATTACACTGGCCAATGGAGAGATTCGGAAGCGACCACTTATCGAAACAAACGGAGAAACAGGAGAAATCGTGTGGGACAAGGGTAGGGATTTCGCGACAGTCCGGAAGGTCCTGTCCATGCCGCAGGTGAACATCGTTAAAAAGACCGAAGTACAGACCGGAGGCTTCTCCAAGGAAAGTATCCTCCCGAAAAGGAACAGCGACAAGCTGATCGCACGCAAAAAAGATTGGGACCCCAAGAAATACGGCGGATTCGATTCTCCTACAGTCGCTTACAGTGTACTGGTTGTGGCCAAAGTGGAGAAAGGGAAGTCTAAAAAACTCAAAAGCGTCAAGGAACTGCTGGGCATCACAATCATGGAGCGATCAAGCTTCGAAAAAAACCCCATCGACTTTCTCGAGGCGAAAGGATATAAAGAGGTCAAAAAAGACCTCATCATTAAGCTTCCCAAGTACTCTCTCTTTGAGCTTGAAAACGGCCGGAAACGAATGCTCGCTAGTGCGGGCGAGCTGCAGAAAGGTAACGAGCTGGCACTGCCCTCTAAATACGTTAATTTCTTGTATCTGGCCAGCCACTATGAAAAGCTCAAAGGGTCTCCCGAAGATAATGAGCAGAAGCAGCTGTTCGTGGAACAACACAAACACTACCTTGATGAGATCATCGAGCAAATAAGCGAATTCTCCAAAAGAGTGATCCTCGCCGACGCTAACCTCGATAAGGTGCTTTCTGCTTACAATAAGCACAGGGATAAGCCCATCAGGGAGCAGGCAGAAAACATTATCCACTTGTTTACTCTGACCAACTTGGGCGCGCCTGCAGCCTTCAAGTACTTCGACACCACCATAGACAGAAAGCGGTACACCTCTACAAAGGAGGTCCTGGACGCCACACTGATTCATCAGTCAATTACGGGGCTCTATGAAACAAGAATCGACCTCTCTCAGCTCGGTGGAGACAGCAGGGCTGACCCCAAGAAGAAGAGGAAGGTGTGA**ATCTCATAAGAATGGTGGTGATTATATATCTTATGTTATTAAGAATTCTCAAATTATTCTATATGAAAACACCGTAACTTGCTTCTCTCCTTGGTTTTACATAATGACATAATGCGATCGAAAACTAGAGGTACAGGTATTGCTGGATTGGCGAGAGTTTTTACCTTCTTTTCTGGCGTACAGCTATCACCTTCTCGTTTGGTAAAATGAAAGAACATTTTGTTGTCTTAGCCAAATATTTAATCTATGAAGAAAACGGAGTTTACCGGTAATTCTAAATAAAAGTTTGGTTAGGGATTGTGCCTCATAGAGAAGCAATTGGTACTCATCTTATTAAAGTATTACTATAAACATTAGAAAGAGTCCCTGAGCGTTGCTAATGGGAAGCTATTCGCGCTTTTAGTAAATTATTAAAATACGCCAAAAATAAATGTAATCCGGATATACCTCTTCTTTTAACCTTCC**ACTAGT**ggatcccccgggctgcaggaattcgatatcaagcttatcgataccgtcgacctcgagggggggcccggtacccagcttttgttccctttagtgagggttaattccgagcttggcgtaatcatggtcatagctgtttcctgtgtg

**TAP(30)-STOP-MX(term):**

pGF-pUC57+TAP(30)-STOP-MX(term)

**GGTCGACGGATCCCCGGGTTAATTAATCCATAA**TCAGTACTGACAATAAAAAGATTCTTGTTTTCAAGAACTTGTCATTTGTATAGTTTTTTTATATTGTAGTTGTTCTATTTTAATCAAATGTTAGCGTGATTTATATTTTTTTTCGCCTCGACATCATCTGCCCAGATGCGAAGTTAAGTGCGCAGAAAGTAATATCATGCGTCAATCGTATGTGAATGCTGGTCGCTATACTGCTGTCGATTCGATACTAACGCCGCCATCCAGT

**prMX-prSHS1-GFP(**β**10)-Linker:**

pGF-TOPO+IVL1334

GTTTAGCTTGCCTCGTCCCCGCCGGGTCACCCGGCCAGCGACATGGAGGCCCAGAATACCCTCCTTGACAGTCTTGACGTGCGCAGCTCAGGGGCATGATGTGACTGTCGCCCGTACATTTAGCCCATACATCCCCATGTATAATCATTTGCATCCATACATTTTGATGGCCGCACGGCGCGAAGCAAAAATTACGGCTCCTCGCTGCAGACCTGCGAGCAGGGAAACGCTCCCCTCACAGACGCGTTGAATTGTCCCCACGCCGCGCCCCTGTAGAGAAATATAAAAGGTTAGGATTTGCCACTGAGGTTCTTCTTTCATATACTTCCTTTTAAAATCTTGCTAGGATACAGTTCTCACATCACATCCGAACATAAACAAGGTTATCGTATTTCACTTTTTGTGGTAAACTCAGGTCAATATTATGCGACTTGAACCATTCAGTCAAAGGTTCGTTACCACCTTTTTCCATACGAAGAAGTTCCTCCGGTTTAAACTGATCCATAGTGATAGATCTTACAAAAGATATATGCACACCAAGCCCTCTATGGATACCGGCACATTCAAGGCAAATGAAAGCTCCAAACTTAGGCGTGGCCCATTGTGGATTTGGCGCACCACAATCCATACATTTCTTATTTGCACCAATCTTTTGCAATTGCAAAAGACGCCTGCGGGTATCTGGGTCCACTTTCCAATCTGACATGCTCTATAATCCGCGATAAAATTGCTCAATTGGCACCATTTAAACTCAGAATCACGTCCATATTTCTGCTTTCATTCTTGATATAGTTGTGCAATTTGGTTCTTGACAAAAACTGGCGTTGCTTGCGGGTAACCGCGCGATTTTTAAAGTGCCAAACTGCGAAAAGAATATAACAAGCTTTCGAGCAAGATCAATGTACCCAGCAAGTGAAATAATAAAACAAGAGCCCCAAAGATCTGCTTATAATTGCTAGAAAAATATATTATTAATC**ATGGACTTGCCAGACGATCATTACTTATCAACCCAAACTATCTTATCAAAGGACTTAAACGATGTAGGTGGTGGTGGTTCAGAAGGTGGTGGTTCTGGTGGTCCAGGTTCAGGTGGTGAAGGTTCCGCTGGTGGTGGTTCCGCAGGTGGTGGTTCC**AAAAAGAAGAAAGCAACT**GAGCTC**

**MXpr-prCDC11-GFP(**β**10)-Linker:**

pGF-TOPO+IVL1335

GTTTAGCTTGCCTCGTCCCCGCCGGGTCACCCGGCCAGCGACATGGAGGCCCAGAATACCCTCCTTGACAGTCTTGACGTGCGCAGCTCAGGGGCATGATGTGACTGTCGCCCGTACATTTAGCCCATACATCCCCATGTATAATCATTTGCATCCATACATTTTGATGGCCGCACGGCGCGAAGCAAAAATTACGGCTCCTCGCTGCAGACCTGCGAGCAGGGAAACGCTCCCCTCACAGACGCGTTGAATTGTCCCCACGCCGCGCCCCTGTAGAGAAATATAAAAGGTTAGGATTTGCCACTGAGGTTCTTCTTTCATATACTTCCTTTTAAAATCTTGCTAGGATACAGTTCTCACATCACATCCGAACATAAACAACCATGTATTTACTGACTTTGCAAAAGTCCAAATATCTGTAAATTAACATGCTATTATAAATATATATATATATATATATTTGAATTTTCTATTTTTTCATCTACAAAAGCAGGTTATAGCTCCGTTAAACCCTTTGCTCATTGAATTGTCCTTCACGGTTTTTTGGTGGAAAACACAACATGGAACATAACATTTAAACATCGTTCTCAATCCATTTCATCGCAGTAACATATTTGGTCGGGCACATACGGACACGTATATGTTATACAGATAAATATAGCTTAAATATGGCTATATTTACACAACACAATGCTCGTGGGTCGTTACCCGACCATGCAGGATCGCCAGTATTCTTTATTTTTCCGCCTTCTAACATAAAGAAAAATAAACAAAAAAGTATTTGATCGAAAAGTAAAATAGGTAGACACCACGTATTGGCGACCCGATCTGGAGCCGTTTAGAAAGTCAATCATCACAAGGCCTAAAGTTGCTAACCACCAGCC**ATGGACTTGCCAGACGATCATTACTTATCAACCCAAACTATCTTATCAAAGGACTTAAACGATGTAGGTGGTGGTGGTTCAGAAGGTGGTGGTTCTGGTGGTCCAGGTTCAGGTGGTGAAGGTTCCGCTGGTGGTGGTTCCGCAGGTGGTGGTTCC**AAAAAGAAGAAAGCAACT**GAGCTC**

**TAP(link)-1xFLAG-Linker-GFP(β11)-SHS1(term)-MX(term):**

pGF-TOPO+IVL1204

**GGTCGACGGATCCCCGGGTTAATTAATCCAGATTACAAAGATGATGACGACAAAGGTTCTGGTGCTGGTGGTAGTCCTGGTGGTGGTTCTGGTGGTTCTGGTTCTTCAGCTTCTGGTGGTTCTACTTCTGAAAAGAGAGATCACATGGTTTTGTTAGAATACGTTACTGCCGCTGGTATCACCGACGCCTCCTGA**GTTGTATCTGTACAAAATCCAAAGCTGAGCAAATAAATAAATAAATAAATGTATAAGTTACCGAACGGGGGTATTTTTACTTTTGATCAAAAATTTATGTACCAACTACAAAGTTTCCTCAGCACAGCCTTCAAGAAGGGAACACACATACAAACAGTGTCAAATAATTGTAGGGATAAATTTAAATATGGCATAAACTAAATAAGTAGAGCATGAAAAAACTGCAAAATCCAAAAAGTAAAAACGAAGGTCAGAAAGTAAAGCAAAAGAAAATTAATAAAGCAATACTAAATCTATCATGATTTCCCGTAACTTCCATTAAAGCTGTAACCAGATTTACTCCTACTGTTTGAGCCTCTAACGCCTAATGGATTTTTAGAGAAGCTCAACCTGATACCTCCTTTGTTGTTGAGGGAAGGGCGGGGGTGAGGTAGTTGACTACCATATAATTCTGCCAATGCTCTAGTGGCAAAGCTAACATCCTCATCAGTACTGACAATAAAAAGATTCTTGTTTTCAAGAACTTGTCATTTGTATAGTTTTTTTATATTGTAGTTGTTCTATTTTAATCAAATGTTAGCGTGATTTATATTTTTTTTCGCCTCGACATCATCTGCCCAGATGCGAAGTTAAGTGCGCAGAAAGTAATATCATGCGTCAATCGTATGTGAATGCTGGTCGCTATACTGCTGTCGATTCGATACTAACGCCGCCATCCAGT

**TAP(link)-1xFLAG-Linker-SpeI-6xHIS-STOP-SHS1(term)-MX(term):**

pGF-TOPO+IVL1205

**GGTCGACGGATCCCCGGGTTAATTAATCCAGATTACAAAGATGATGACGACAAAGGTTCTGGTGCTGGTGGTAGTCCTGGTGGTGGTTCTGGTGGTTCTGGTTCTTCAGCTTCTGGTGGTTCTACTTCTACTAGTCATCACCACCATCATCATTGA**GTTGTATCTGTACAAAATCCAAAGCTGAGCAAATAAATAAATAAATAAATGTATAAGTTACCGAACGGGGGTATTTTTACTTTTGATCAAAAATTTATGTACCAACTACAAAGTTTCCTCAGCACAGCCTTCAAGAAGGGAACACACATACAAACAGTGTCAAATAATTGTAGGGATAAATTTAAATATGGCATAAACTAAATAAGTAGAGCATGAAAAAACTGCAAAATCCAAAAAGTAAAAACGAAGGTCAGAAAGTAAAGCAAAAGAAAATTAATAAAGCAATACTAAATCTATCATGATTTCCCGTAACTTCCATTAAAGCTGTAACCAGATTTACTCCTACTGTTTGAGCCTCTAACGCCTAATGGATTTTTAGAGAAGCTCAACCTGATACCTCCTTTGTTGTTGAGGGAAGGGCGGGGGTGAGGTAGTTGACTACCATATAATTCTGCCAATGCTCTAGTGGCAAAGCTAACATCCTCATCAGTACTGACAATAAAAAGATTCTTGTTTTCAAGAACTTGTCATTTGTATAGTTTTTTTATATTGTAGTTGTTCTATTTTAATCAAATGTTAGCGTGATTTATATTTTTTTTCGCCTCGACATCATCTGCCCAGATGCGAAGTTAAGTGCGCAGAAAGTAATATCATGCGTCAATCGTATGTGAATGCTGGTCGCTATACTGCTGTCGATTCGATACTAACGCCGCCATCCAGT

**NotI-TAP(link)-mCherry(opt)-STOP-SHS1(term)-MX(term):**

pGF-TOPO+IVL1206

**GCGGCCGCGGTCGACGGATCCCCGGGTTAATTAATCCA**gtgagcaagggcgaggaggataacatggccatcat**TaaggaAttTatgAgAttTaaAg**TTCACATGGAAGGTTCAGTTAACGGTCATGAATTTGAAATCGAAGGTGAAGGTGAAGGTAGACCATACGAAGGTACTCAAACAGCTAAATTGAAGGTTACTAAAGGTGGTCCATTGCCATTTGCATGGGATATTTTGTCTCCACAATTCATGTACGGTTCAAAGGCTTATGTTAAACATCCAGCAGATATTCCAGATTACTTGAAATTATCTTTTCCAGAAGGTTTTAAATGGGAAAGAGTTATGAACTTCGAAGATGGTGGTGTTGTTACTGTTACACAAGATTCTTCATTGCAAGATGGCGAGTTTATATATAAGGTTAAATTGAGAGGTACTAATTTTCCATCAGATGGTCCAGTTATGCAAAAGAAAACTATGGGTTGGGAAGCATCTTCTGAAAGAATGTACCCAGAAGATGGTGCATTGAAGGGTGAAATTAAACAAAGATTGAAATTGAAGGATGGTGGTCATTACGATGCTGAAGTTAAGACTACATACAAGGCTAAGAAACCAGTTCAATTGCCAGGTGCTTACAACGTTAACATCAAATTGGATATTACTTCTCATAATGAAGATTATACAATTGTTGAACAATACGAAAGAGCAGAAGGTAGACATTCAACAGGTGGTATGGATGAATTATACAAA**TGA**GTTGTATCTGTACAAAATCCAAAGCTGAGCAAATAAATAAATAAATAAATGTATAAGTTACCGAACGGGGGTATTTTTACTTTTGATCAAAAATTTATGTACCAACTACAAAGTTTCCTCAGCACAGCCTTCAAGAAGGGAACACACATACAAACAGTGTCAAATAATTGTAGGGATAAATTTAAATATGGCATAAACTAAATAAGTAGAGCATGAAAAAACTGCAAAATCCAAAAAGTAAAAACGAAGGTCAGAAAGTAAAGCAAAAGAAAATTAATAAAGCAATACTAAATCTATCATGATTTCCCGTAACTTCCATTAAAGCTGTAACCAGATTTACTCCTACTGTTTGAGCCTCTAACGCCTAATGGATTTTTAGAGAAGCTCAACCTGATACCTCCTTTGTTGTTGAGGGAAGGGCGGGGGTGAGGTAGTTGACTACCATATAATTCTGCCAATGCTCTAGTGGCAAAGCTAACATCCTCATCAGTACTGACAATAAAAAGATTCTTGTTTTCAAGAACTTGTCATTTGTATAGTTTTTTTATATTGTAGTTGTTCTATTTTAATCAAATGTTAGCGTGATTTATATTTTTTTTCGCCTCGACATCATCTGCCCAGATGCGAAGTTAAGTGCGCAGAAAGTAATATCATGCGTCAATCGTATGTGAATGCTGGTCGCTATACTGCTGTCGATTCGATACTAACGCCGCCATCCAGT

**NotI-TAP(link)-[anti-GFP nanobody(opt)]-STOP-SHS1(term)-MX(term):**

pGF-TOPO+IVL1207C

**GCGGCCGCGGTCGACGGATCCCCGGGTTAATTAATCCAATGCAAGTCCAATTAGTAGAATCCGGTGGTGCCTTAGTCCAACCTGGTGGTAGTTTGAGATTATCCTGTGCCGCCTCTGGTTTCCCAGTAAATAGATATTCCATGAGATGGTACAGACAAGCACCTGGTAAAGAAAGAGAATGGGTCGCTGGCATGTCTTCAGCAGGTGACAGATCCAGTTATGAAGACTCTGTTAAGGGTAGATTCACTATTTCAAGAGATGACGCCAGAAACACAGTTTACTTGCAAATGAACTCATTAAAACCTGAAGATACCGCTGTATATTACTGTAACGTCAATGTCGGTTTCGAATACTGGGGTCAAGGTACTCAAGTTACTGTTTCCTCAAAATGA**GTTGTATCTGTACAAAATCCAAAGCTGAGCAAATAAATAAATAAATAAATGTATAAGTTACCGAACGGGGGTATTTTTACTTTTGATCAAAAATTTATGTACCAACTACAAAGTTTCCTCAGCACAGCCTTCAAGAAGGGAACACACATACAAACAGTGTCAAATAATTGTAGGGATAAATTTAAATATGGCATAAACTAAATAAGTAGAGCATGAAAAAACTGCAAAATCCAAAAAGTAAAAACGAAGGTCAGAAAGTAAAGCAAAAGAAAATTAATAAAGCAATACTAAATCTATCATGATTTCCCGTAACTTCCATTAAAGCTGTAACCAGATTTACTCCTACTGTTTGAGCCTCTAACGCCTAATGGATTTTTAGAGAAGCTCAACCTGATACCTCCTTTGTTGTTGAGGGAAGGGCGGGGGTGAGGTAGTTGACTACCATATAATTCTGCCAATGCTCTAGTGGCAAAGCTAACATCCTCATCAGTACTGACAATAAAAAGATTCTTGTTTTCAAGAACTTGTCATTTGTATAGTTTTTTTATATTGTAGTTGTTCTATTTTAATCAAATGTTAGCGTGATTTATATTTTTTTTCGCCTCGACATCATCTGCCCAGATGCGAAGTTAAGTGCGCAGAAAGTAATATCATGCGTCAATCGTATGTGAATGCTGGTCGCTATACTGCTGTCGATTCGATACTAACGCCGCCATCCAGT

**NotI-TAP(link)-GST-STOP-SHS1(term)-MX(term):**

pGF-TOPO+IVL1208

**GCGGCCGCGGTCGACGGATCCCCGGGTTAATTAATCCATCCCCTATACTAGGTTATTGGAAAATTAAGGGCCTTGTGCAACCCACTCGACTTCTTTTGGAATATCTTGAAGAAAAATATGAAGAGCATTTGTATGAGCGCGATGAAGGTGATAAATGGCGAAACAAAAAGTTTGAATTGGGTTTGGAGTTTCCCAATCTTCCTTATTATATTGATGGTGATGTTAAATTAACACAGTCTATGGCCATCATACGTTATATAGCTGACAAGCACAACATGTTGGGTGGTTGTCCAAAAGAGCGTGCAGAGATTTCAATGCTTGAAGGAGCGGTTTTGGATATTAGATACGGTGTTTCGAGAATTGCATATAGTAAAGACTTTGAAACTCTCAAAGTTGATTTTCTTAGCAAGCTACCTGAAATGCTGAAAATGTTCGAAGATCGTTTATGTCATAAAACATATTTAAATGGTGATCATGTAACCCATCCTGACTTCATGTTGTATGACGCTCTTGATGTTGTTTTATACATGGACCCAATGTGCCTGGATGCGTTCCCAAAATTAGTTTGTTTTAAAAAACGTATTGAAGCTATCCCACAAATTGATAAGTACTTGAAATCCAGCAAGTATATAGCATGGCCTTTGCAGGGCTGGCAAGCCACGTTTGGTGGTGGCGACCATCCTCCAAAATCGGATCTGGTTCCGCGTGGATCCTGA**GTTGTATCTGTACAAAATCCAAAGCTGAGCAAATAAATAAATAAATAAATGTATAAGTTACCGAACGGGGGTATTTTTACTTTTGATCAAAAATTTATGTACCAACTACAAAGTTTCCTCAGCACAGCCTTCAAGAAGGGAACACACATACAAACAGTGTCAAATAATTGTAGGGATAAATTTAAATATGGCATAAACTAAATAAGTAGAGCATGAAAAAACTGCAAAATCCAAAAAGTAAAAACGAAGGTCAGAAAGTAAAGCAAAAGAAAATTAATAAAGCAATACTAAATCTATCATGATTTCCCGTAACTTCCATTAAAGCTGTAACCAGATTTACTCCTACTGTTTGAGCCTCTAACGCCTAATGGATTTTTAGAGAAGCTCAACCTGATACCTCCTTTGTTGTTGAGGGAAGGGCGGGGGTGAGGTAGTTGACTACCATATAATTCTGCCAATGCTCTAGTGGCAAAGCTAACATCCTCATCAGTACTGACAATAAAAAGATTCTTGTTTTCAAGAACTTGTCATTTGTATAGTTTTTTTATATTGTAGTTGTTCTATTTTAATCAAATGTTAGCGTGATTTATATTTTTTTTCGCCTCGACATCATCTGCCCAGATGCGAAGTTAAGTGCGCAGAAAGTAATATCATGCGTCAATCGTATGTGAATGCTGGTCGCTATACTGCTGTCGATTCGATACTAACGCCGCCATCCAGT

**NotI-TAP(link)-1xFLAG-Linker-3xHA-STOP-SHS1(term)-MX(term):**

pGF-TOPO+IVL1209

**GCGGCCGCGGTCGACGGATCCCCGGGTTAATTAATCCAGATTACAAAGATGATGACGACAAAGGTTCTGGTGCTGGTGGTAGTCCTGGTGGTGGTTCTGGTGGTTCTGGTTCTTCAGCTTCTGGTGGTTCTACTTCTTACCCATACGATGTTCCTGACTATGCGGGCTATCCCTATGACGTCCCGGACTATGCAGGATCCTATCCATATGACGTTCCAGATTACGCTTGCGGCTGA**GTTGTATCTGTACAAAATCCAAAGCTGAGCAAATAAATAAATAAATAAATGTATAAGTTACCGAACGGGGGTATTTTTACTTTTGATCAAAAATTTATGTACCAACTACAAAGTTTCCTCAGCACAGCCTTCAAGAAGGGAACACACATACAAACAGTGTCAAATAATTGTAGGGATAAATTTAAATATGGCATAAACTAAATAAGTAGAGCATGAAAAAACTGCAAAATCCAAAAAGTAAAAACGAAGGTCAGAAAGTAAAGCAAAAGAAAATTAATAAAGCAATACTAAATCTATCATGATTTCCCGTAACTTCCATTAAAGCTGTAACCAGATTTACTCCTACTGTTTGAGCCTCTAACGCCTAATGGATTTTTAGAGAAGCTCAACCTGATACCTCCTTTGTTGTTGAGGGAAGGGCGGGGGTGAGGTAGTTGACTACCATATAATTCTGCCAATGCTCTAGTGGCAAAGCTAACATCCTCATCAGTACTGACAATAAAAAGATTCTTGTTTTCAAGAACTTGTCATTTGTATAGTTTTTTTATATTGTAGTTGTTCTATTTTAATCAAATGTTAGCGTGATTTATATTTTTTTTCGCCTCGACATCATCTGCCCAGATGCGAAGTTAAGTGCGCAGAAAGTAATATCATGCGTCAATCGTATGTGAATGCTGGTCGCTATACTGCTGTCGATTCGATACTAACGCCGCCATCCAGT

**NotI-TAP(link)-NLS-STOP-SHS1(term)-MX(term):**

pGF-TOPO+IVL1302

**GCGGCCGCGGTCGACGGATCCCCGGGTTAATTAATCCATCTAGGGCAGACCCAAAGAAAAAGAGGAAAGTATGA**GTTGTATCTGTACAAAATCCAAAGCTGAGCAAATAAATAAATAAATAAATGTATAAGTTACCGAACGGGGGTATTTTTACTTTTGATCAAAAATTTATGTACCAACTACAAAGTTTCCTCAGCACAGCCTTCAAGAAGGGAACACACATACAAACAGTGTCAAATAATTGTAGGGATAAATTTAAATATGGCATAAACTAAATAAGTAGAGCATGAAAAAACTGCAAAATCCAAAAAGTAAAAACGAAGGTCAGAAAGTAAAGCAAAAGAAAATTAATAAAGCAATACTAAATCTATCATGATTTCCCGTAACTTCCATTAAAGCTGTAACCAGATTTACTCCTACTGTTTGAGCCTCTAACGCCTAATGGATTTTTAGAGAAGCTCAACCTGATACCTCCTTTGTTGTTGAGGGAAGGGCGGGGGTGAGGTAGTTGACTACCATATAATTCTGCCAATGCTCTAGTGGCAAAGCTAACATCCTCATCAGTACTGACAATAAAAAGATTCTTGTTTTCAAGAACTTGTCATTTGTATAGTTTTTTTATATTGTAGTTGTTCTATTTTAATCAAATGTTAGCGTGATTTATATTTTTTTTCGCCTCGACATCATCTGCCCAGATGCGAAGTTAAGTGCGCAGAAAGTAATATCATGCGTCAATCGTATGTGAATGCTGGTCGCTATACTGCTGTCGATTCGATACTAACGCCGCCATCCAGT

**NotI-TAP(link)-NES-STOP-SHS1(term)-MX(term):**

pGF-TOPO+IVL1303

**GCGGCCGCGGTCGACGGATCCCCGGGTTAATTAATCCATTGGCTAAAATTTTGGGTGCTTTGGATATTAATTGA**GTTGTATCTGTACAAAATCCAAAGCTGAGCAAATAAATAAATAAATAAATGTATAAGTTACCGAACGGGGGTATTTTTACTTTTGATCAAAAATTTATGTACCAACTACAAAGTTTCCTCAGCACAGCCTTCAAGAAGGGAACACACATACAAACAGTGTCAAATAATTGTAGGGATAAATTTAAATATGGCATAAACTAAATAAGTAGAGCATGAAAAAACTGCAAAATCCAAAAAGTAAAAACGAAGGTCAGAAAGTAAAGCAAAAGAAAATTAATAAAGCAATACTAAATCTATCATGATTTCCCGTAACTTCCATTAAAGCTGTAACCAGATTTACTCCTACTGTTTGAGCCTCTAACGCCTAATGGATTTTTAGAGAAGCTCAACCTGATACCTCCTTTGTTGTTGAGGGAAGGGCGGGGGTGAGGTAGTTGACTACCATATAATTCTGCCAATGCTCTAGTGGCAAAGCTAACATCCTCATCAGTACTGACAATAAAAAGATTCTTGTTTTCAAGAACTTGTCATTTGTATAGTTTTTTTATATTGTAGTTGTTCTATTTTAATCAAATGTTAGCGTGATTTATATTTTTTTTCGCCTCGACATCATCTGCCCAGATGCGAAGTTAAGTGCGCAGAAAGTAATATCATGCGTCAATCGTATGTGAATGCTGGTCGCTATACTGCTGTCGATTCGATACTAACGCCGCCATCCAGT

**NotI-TAP(link)-1xMYC-STOP-SHS1(term)-MX(term):**

pGF-TOPO+IVL1304

**GCGGCCGCGGTCGACGGATCCCCGGGTTAATTAATCCAGAACAAAAATTAATTTCTGAAGAAGATTTGTGA**GTTGTATCTGTACAAAATCCAAAGCTGAGCAAATAAATAAATAAATAAATGTATAAGTTACCGAACGGGGGTATTTTTACTTTTGATCAAAAATTTATGTACCAACTACAAAGTTTCCTCAGCACAGCCTTCAAGAAGGGAACACACATACAAACAGTGTCAAATAATTGTAGGGATAAATTTAAATATGGCATAAACTAAATAAGTAGAGCATGAAAAAACTGCAAAATCCAAAAAGTAAAAACGAAGGTCAGAAAGTAAAGCAAAAGAAAATTAATAAAGCAATACTAAATCTATCATGATTTCCCGTAACTTCCATTAAAGCTGTAACCAGATTTACTCCTACTGTTTGAGCCTCTAACGCCTAATGGATTTTTAGAGAAGCTCAACCTGATACCTCCTTTGTTGTTGAGGGAAGGGCGGGGGTGAGGTAGTTGACTACCATATAATTCTGCCAATGCTCTAGTGGCAAAGCTAACATCCTCATCAGTACTGACAATAAAAAGATTCTTGTTTTCAAGAACTTGTCATTTGTATAGTTTTTTTATATTGTAGTTGTTCTATTTTAATCAAATGTTAGCGTGATTTATATTTTTTTTCGCCTCGACATCATCTGCCCAGATGCGAAGTTAAGTGCGCAGAAAGTAATATCATGCGTCAATCGTATGTGAATGCTGGTCGCTATACTGCTGTCGATTCGATACTAACGCCGCCATCCAGT

**NotI-TAP(link)-MBP-STOP-SHS1(term)-MX(term):**

pGF-TOPO+IVL1305

**GCGGCCGCGGTCGACGGATCCCCGGGTTAATTAATCCAAAAATCGAAGAAGGTAAACTGGTAATCTGGATTAACGGCGATAAAGGCTATAACGGTCTCGCTGAAGTCGGTAAGAAATTCGAGAAAGATACCGGAATTAAAGTCACCGTTGAGCATCCGGATAAACTGGAAGAGAAATTCCCACAGGTTGCGGCAACTGGCGATGGCCCTGACATTATCTTCTGGGCACACGACCGCTTTGGTGGCTACGCTCAATCTGGCCTGTTGGCTGAAATCACCCCGGACAAAGCGTTCCAGGACAAGCTGTATCCGTTTACCTGGGATGCCGTACGTTACAACGGCAAGCTGATTGCTTACCCGATCGCTGTTGAAGCGTTATCGCTGATTTATAACAAAGATCTGCTGCCGAACCCGCCAAAAACCTGGGAAGAGATCCCGGCGCTGGATAAAGAACTGAAAGCGAAAGGTAAGAGCGCGCTGATGTTCAACCTGCAAGAACCGTACTTCACCTGGCCGCTGATTGCTGCTGACGGGGGTTATGCGTTCAAGTATGAAAACGGCAAGTACGACATTAAAGACGTGGGCGTGGATAACGCTGGCGCGAAAGCGGGTCTGACCTTCCTGGTTGACCTGATTAAAAACAAACACATGAATGCAGACACCGATTACTCCATCGCAGAAGCTGCCTTTAATAAAGGCGAAACAGCGATGACCATCAACGGCCCGTGGGCATGGTCCAACATCGACACCAGCAAAGTGAATTATGGTGTAACGGTACTGCCGACCTTCAAGGGTCAACCATCCAAACCGTTCGTTGGCGTGCTGAGCGCAGGTATTAACGCCGCCAGTCCGAACAAAGAGCTGGCAAAAGAGTTCCTCGAAAACTATCTGCTGACTGATGAAGGTCTGGAAGCGGTTAATAAAGACAAACCGCTGGGTGCCGTAGCGCTGAAGTCTTACGAGGAAGAGTTGGCGAAAGATCCACGTATTGCCGCCACCATGGAAAACGCCCAGAAAGGTGAAATCATGCCGAACATCCCGCAGATGTCCGCTTTCTGGTATGCCGTGCGTACTGCGGTGATCAACGCCGCCAGCGGTCGTCAGACTGTCGATGAAGCCCTGAAAGACGCGCAGACTAATTCGAGCTCGGCGCGCCTGctcgagTGA**GTTGTATCTGTACAAAATCCAAAGCTGAGCAAATAAATAAATAAATAAATGTATAAGTTACCGAACGGGGGTATTTTTACTTTTGATCAAAAATTTATGTACCAACTACAAAGTTTCCTCAGCACAGCCTTCAAGAAGGGAACACACATACAAACAGTGTCAAATAATTGTAGGGATAAATTTAAATATGGCATAAACTAAATAAGTAGAGCATGAAAAAACTGCAAAATCCAAAAAGTAAAAACGAAGGTCAGAAAGTAAAGCAAAAGAAAATTAATAAAGCAATACTAAATCTATCATGATTTCCCGTAACTTCCATTAAAGCTGTAACCAGATTTACTCCTACTGTTTGAGCCTCTAACGCCTAATGGATTTTTAGAGAAGCTCAACCTGATACCTCCTTTGTTGTTGAGGGAAGGGCGGGGGTGAGGTAGTTGACTACCATATAATTCTGCCAATGCTCTAGTGGCAAAGCTAACATCCTCATCAGTACTGACAATAAAAAGATTCTTGTTTTCAAGAACTTGTCATTTGTATAGTTTTTTTATATTGTAGTTGTTCTATTTTAATCAAATGTTAGCGTGATTTATATTTTTTTTCGCCTCGACATCATCTGCCCAGATGCGAAGTTAAGTGCGCAGAAAGTAATATCATGCGTCAATCGTATGTGAATGCTGGTCGCTATACTGCTGTCGATTCGATACTAACGCCGCCATCCAGT

**NotI-TAP(link)-BirA(R118G)-STOP-SHS1(term)-MX(term):**

pGF-TOPO+IVL1306

**GCGGCCGCGGTCGACGGATCCCCGGGTTAATTAATCCAATGAAGGATAACACCGTGCCACTGAAATTGATTGCCCTGTTAGCGAACGGTGAATTTCACTCTGGCGAGCAGTTGGGTGAAACGCTGGGAATGAGCCGGGCGGCTATTAATAAACACATTCAGACACTGCGTGACTGGGGCGTTGATGTCTTTACCGTTCCGGGTAAAGGATACAGCCTGCCTGAGCCTATCCAGTTACTTAATGCTAAACAGATATTGGGTCAGCTGGATGGCGGTAGTGTAGCCGTGCTGCCAGTGATTGACTCCACGAATCAGTACCTTCTTGATCGTATCGGAGAGCTTAAATCGGGCGATGCTTGCATTGCAGAATACCAGCAGGCTGGCCGTGGTGGCCGGGGTCGGAAATGGTTTTCGCCTTTTGGCGCAAACTTATATTTGTCGATGTTCTGGCGTCTGGAACAAGGCCCGGCGGCGGCGATTGGTTTAAGTCTGGTTATCGGTATCGTGATGGCGGAAGTATTACGCAAGCTGGGTGCAGATAAAGTTCGTGTTAAATGGCCTAATGACCTCTATCTGCAGGATCGCAAGCTGGCAGGCATTCTGGTGGAGCTGACTGGCAAAACTGGCGATGCGGCGCAAATAGTCATTGGAGCCGGGATCAACATGGCAATGCGCCGTGTTGAAGAGAGTGTCGTTAATCAGGGGTGGATCACGCTGCAGGAAGCGGGGATCAATCTCGATCGTAATACGTTGGCGGCCATGCTAATACGTGAATTACGTGCTGCGTTGGAACTCTTCGAACAAGAAGGATTGGCACCTTATCTGTCGCGCTGGGAAAAGCTGGATAATTTTATTAATCGCCCAGTGAAACTTATCATTGGTGATAAAGAAATATTTGGCATTTCACGCGGAATAGACAAACAGGGGGCTTTATTACTTGAGCAGGATGGAATAATAAAACCCTGGATGGGCGGTGAAATATCCCTGCGTAGTGCAGAAAAATGA**GTTGTATCTGTACAAAATCCAAAGCTGAGCAAATAAATAAATAAATAAATGTATAAGTTACCGAACGGGGGTATTTTTACTTTTGATCAAAAATTTATGTACCAACTACAAAGTTTCCTCAGCACAGCCTTCAAGAAGGGAACACACATACAAACAGTGTCAAATAATTGTAGGGATAAATTTAAATATGGCATAAACTAAATAAGTAGAGCATGAAAAAACTGCAAAATCCAAAAAGTAAAAACGAAGGTCAGAAAGTAAAGCAAAAGAAAATTAATAAAGCAATACTAAATCTATCATGATTTCCCGTAACTTCCATTAAAGCTGTAACCAGATTTACTCCTACTGTTTGAGCCTCTAACGCCTAATGGATTTTTAGAGAAGCTCAACCTGATACCTCCTTTGTTGTTGAGGGAAGGGCGGGGGTGAGGTAGTTGACTACCATATAATTCTGCCAATGCTCTAGTGGCAAAGCTAACATCCTCATCAGTACTGACAATAAAAAGATTCTTGTTTTCAAGAACTTGTCATTTGTATAGTTTTTTTATATTGTAGTTGTTCTATTTTAATCAAATGTTAGCGTGATTTATATTTTTTTTCGCCTCGACATCATCTGCCCAGATGCGAAGTTAAGTGCGCAGAAAGTAATATCATGCGTCAATCGTATGTGAATGCTGGTCGCTATACTGCTGTCGATTCGATACTAACGCCGCCATCCAGT

**NotI-TAP(link)-CAAX-STOP-SHS1(term)-MX(term):**

pGF-TOPO+IVL1307

**GCGGCCGCGGTCGACGGATCCCCGGGTTAATTAATCCA**GGTTCTGGTGGATGTTGTATTATTTCT**TGA**GTTGTATCTGTACAAAATCCAAAGCTGAGCAAATAAATAAATAAATAAATGTATAAGTTACCGAACGGGGGTATTTTTACTTTTGATCAAAAATTTATGTACCAACTACAAAGTTTCCTCAGCACAGCCTTCAAGAAGGGAACACACATACAAACAGTGTCAAATAATTGTAGGGATAAATTTAAATATGGCATAAACTAAATAAGTAGAGCATGAAAAAACTGCAAAATCCAAAAAGTAAAAACGAAGGTCAGAAAGTAAAGCAAAAGAAAATTAATAAAGCAATACTAAATCTATCATGATTTCCCGTAACTTCCATTAAAGCTGTAACCAGATTTACTCCTACTGTTTGAGCCTCTAACGCCTAATGGATTTTTAGAGAAGCTCAACCTGATACCTCCTTTGTTGTTGAGGGAAGGGCGGGGGTGAGGTAGTTGACTACCATATAATTCTGCCAATGCTCTAGTGGCAAAGCTAACATCCTCATCAGTACTGACAATAAAAAGATTCTTGTTTTCAAGAACTTGTCATTTGTATAGTTTTTTTATATTGTAGTTGTTCTATTTTAATCAAATGTTAGCGTGATTTATATTTTTTTTCGCCTCGACATCATCTGCCCAGATGCGAAGTTAAGTGCGCAGAAAGTAATATCATGCGTCAATCGTATGTGAATGCTGGTCGCTATACTGCTGTCGATTCGATACTAACGCCGCCATCCAGT

**NotI-TAP(link)-SNAP-STOP-SHS1(term)-MX(term):**

pGF-TOPO+IVL1309

**GCGGCCGCGGTCGACGGATCCCCGGGTTAATTAATCCAATGGATAAGGATTGCGAAATGAAGAGAACTACATTAGATTCCCCATTGGGTAAATTAGAATTGTCCGGTTGCGAACAAGGTTTGCATAGAATAATATTTTTGGGTAAAGGTACTTCTGCTGCTGATGCTGTAGAAGTCCCAGCACCTGCCGCTGTTTTGGGTGGTCCAGAACCTTTAATGCAAGCAACTGCCTGGTTAAATGCCTATTTTCATCAACCAGAAGCTATAGAAGAATTTCCAGTTCCTGCTTTGCATCACCCTGTATTTCAACAAGAATCCTTCACAAGACAAGTATTGTGGAAATTGTTGAAGGTTGTAAAGTTCGGTGAAGTTATTTCTTACTCACATTTGGCAGCCTTAGCAGGTAATCCAGCTGCAACTGCCGCTGTCAAAACAGCCTTGAGTGGTAACCCAGTTCCTATCTTAATTCCTTGTCACAGAGTCGTTCAGGGTGACTTGGATGTTGGTGGTTACGAAGGTGGTTTAGCAGTAAAGGAATGGTTATTGGCTCACGAAGGTCATAGATTGGGTAAACCAGGTTTGGGTTGA**GTTGTATCTGTACAAAATCCAAAGCTGAGCAAATAAATAAATAAATAAATGTATAAGTTACCGAACGGGGGTATTTTTACTTTTGATCAAAAATTTATGTACCAACTACAAAGTTTCCTCAGCACAGCCTTCAAGAAGGGAACACACATACAAACAGTGTCAAATAATTGTAGGGATAAATTTAAATATGGCATAAACTAAATAAGTAGAGCATGAAAAAACTGCAAAATCCAAAAAGTAAAAACGAAGGTCAGAAAGTAAAGCAAAAGAAAATTAATAAAGCAATACTAAATCTATCATGATTTCCCGTAACTTCCATTAAAGCTGTAACCAGATTTACTCCTACTGTTTGAGCCTCTAACGCCTAATGGATTTTTAGAGAAGCTCAACCTGATACCTCCTTTGTTGTTGAGGGAAGGGCGGGGGTGAGGTAGTTGACTACCATATAATTCTGCCAATGCTCTAGTGGCAAAGCTAACATCCTCATCAGTACTGACAATAAAAAGATTCTTGTTTTCAAGAACTTGTCATTTGTATAGTTTTTTTATATTGTAGTTGTTCTATTTTAATCAAATGTTAGCGTGATTTATATTTTTTTTCGCCTCGACATCATCTGCCCAGATGCGAAGTTAAGTGCGCAGAAAGTAATATCATGCGTCAATCGTATGTGAATGCTGGTCGCTATACTGCTGTCGATTCGATACTAACGCCGCCATCCAGT

**NotI-TAP(link)-SpHIS5-STOP-SHS1(term)-MX(term):**

pGF-TOPO+IVL1310

**GCGGCCGCGGTCGACGGATCCCCGGGTTAATTAATCCAATG**aggagggcttttgtagaaagaaatacgaacgaaacgaaaatcagcgttgccatcgctttggacaaagctcccttacctgaagagtcgaattttattgatgaacttataacttccaagcatAcaaaccaaaagggagaacaagtaatccaagtagacacgggaattggattcttggatcacatgtatcatgcactggctaaacatgcaggctggagcttacgactttactcaagaggtgatttaatcatcgatgatcatcacactgcagaagatactgctattgcacttggtattgcattcaagcaggctatgAgtaactttgccggcgttaaaagatttggacatgcttattgtccacttgacgaagctctttctagaagcgtagttgacttgtcgggacggccctatgctgttatcgatttgggattaaagcgtgaaaaggttggggaattgtcctgtgaaatgatccctcacttactatattccttttcggtagcagctggaattactttgcatgttacctgcttatatggtagtaatgaccatcatcgtgctgaaagcgcttttaaatctctggctgttgccatgcgcgcggctactagtcttactggaagttctgaagtcccaagcacgaagggagtgttg**TGA**GTTGTATCTGTACAAAATCCAAAGCTGAGCAAATAAATAAATAAATAAATGTATAAGTTACCGAACGGGGGTATTTTTACTTTTGATCAAAAATTTATGTACCAACTACAAAGTTTCCTCAGCACAGCCTTCAAGAAGGGAACACACATACAAACAGTGTCAAATAATTGTAGGGATAAATTTAAATATGGCATAAACTAAATAAGTAGAGCATGAAAAAACTGCAAAATCCAAAAAGTAAAAACGAAGGTCAGAAAGTAAAGCAAAAGAAAATTAATAAAGCAATACTAAATCTATCATGATTTCCCGTAACTTCCATTAAAGCTGTAACCAGATTTACTCCTACTGTTTGAGCCTCTAACGCCTAATGGATTTTTAGAGAAGCTCAACCTGATACCTCCTTTGTTGTTGAGGGAAGGGCGGGGGTGAGGTAGTTGACTACCATATAATTCTGCCAATGCTCTAGTGGCAAAGCTAACATCCTCATCAGTACTGACAATAAAAAGATTCTTGTTTTCAAGAACTTGTCATTTGTATAGTTTTTTTATATTGTAGTTGTTCTATTTTAATCAAATGTTAGCGTGATTTATATTTTTTTTCGCCTCGACATCATCTGCCCAGATGCGAAGTTAAGTGCGCAGAAAGTAATATCATGCGTCAATCGTATGTGAATGCTGGTCGCTATACTGCTGTCGATTCGATACTAACGCCGCCATCCAGT

**NotI-TAP(link)-mScarlet(opt)-STOP-SHS1(term)-MX(term):**

pGF-TOPO+IVL1311

**GCGGCCGCGGTCGACGGATCCCCGGGTTAATTAATCCAGTTTCTAAGGGTGAAGCTGTTATTAAAGAGTTTATGAGATTCAAAGTTCACATGGAAGGTTCAATGAACGGTCATGAATTTGAAATCGAAGGTGAAGGTGAAGGTAGACCATACGAAGGTACTCAAACAGCAAAATTGAAGGTTACTAAAGGTGGTCCATTGCCATTTTCTTGGGATATCTTGTCTCCACAATTCATGTACGGTTCTAGAGCTTTTACAAAACATCCAGCAGATATCCCAGATTACTACAAGCAATCATTTCCAGAAGGTTTTAAATGGGAAAGAGTTATGAACTTCGAAGATGGTGGTGCTGTTACTGTTACACAAGATACTTCTTTGGAAGATGGTACATTGATCTATAAGGTTAAATTGAGAGGTACTAATTTTCCACCAGATGGTCCAGTTATGCAAAAGAAAACTATGGGTTGGGAAGCATCTACAGAAAGATTGTACCCAGAAGATGGTGTTTTGAAGGGTGACATCAAGATGGCATTGAGATTGAAGGATGGTGGTAGATATTTGGCTGATTTCAAGACTACATACAAGGCTAAGAAACCAGTTCAAATGCCAGGTGCATACAACGTTGATAGAAAATTGGATATCACTTCTCATAACGAAGATTACACAGTTGTTGAACAATACGAAAGATCTGAAGGTAGACATTCAACAGGTGGTATGGATGAATTGTACAAATGA**GTTGTATCTGTACAAAATCCAAAGCTGAGCAAATAAATAAATAAATAAATGTATAAGTTACCGAACGGGGGTATTTTTACTTTTGATCAAAAATTTATGTACCAACTACAAAGTTTCCTCAGCACAGCCTTCAAGAAGGGAACACACATACAAACAGTGTCAAATAATTGTAGGGATAAATTTAAATATGGCATAAACTAAATAAGTAGAGCATGAAAAAACTGCAAAATCCAAAAAGTAAAAACGAAGGTCAGAAAGTAAAGCAAAAGAAAATTAATAAAGCAATACTAAATCTATCATGATTTCCCGTAACTTCCATTAAAGCTGTAACCAGATTTACTCCTACTGTTTGAGCCTCTAACGCCTAATGGATTTTTAGAGAAGCTCAACCTGATACCTCCTTTGTTGTTGAGGGAAGGGCGGGGGTGAGGTAGTTGACTACCATATAATTCTGCCAATGCTCTAGTGGCAAAGCTAACATCCTCATCAGTACTGACAATAAAAAGATTCTTGTTTTCAAGAACTTGTCATTTGTATAGTTTTTTTATATTGTAGTTGTTCTATTTTAATCAAATGTTAGCGTGATTTATATTTTTTTTCGCCTCGACATCATCTGCCCAGATGCGAAGTTAAGTGCGCAGAAAGTAATATCATGCGTCAATCGTATGTGAATGCTGGTCGCTATACTGCTGTCGATTCGATACTAACGCCGCCATCCAGT

**NotI-TAP(link)-eGFP(opt)-STOP-SHS1(term)-MX(term):**

pGF-TOPO+IVL1379

**GCGGCCGCGGTCGACGGATCCCCGGGTTAATTAATCCA**agt**aaGggTgaGgaGTtGttTactgg**TGTTGTTCCAATTTTGGTTGAATTAGATGGTGACGTTAATGGTCATAAATTTTCTGTTTCAGGTGAAGGTGAAGGTGACGCTACTTATGGTAAATTGACATTGAAGTTTATTTGTACTACTGGTAAATTGCCAGTTCCTTGGCCAACTTTGGTTACTACATTGACTTATGGTGTTCAATGTTTTTCAAGATACCCAGATCACATGAAGCAACATGATTTCTTTAAGTCTGCTATGCCAGAAGGTTACGTTCAAGAAAGAACAATTTTCTTTAAAGATGATGGTAACTACAAGACTAGAGCAGAGGTTAAGTTCGAAGGTGACACATTGGTTAACAGAATTGAATTAAAAGGTATTGATTTTAAAGAAGATGGTAATATTTTGGGTCATAAATTAGAATACAACTACAACTCACATAATGTTTATATTATGGCTGATAAACAAAAGAATGGTATTAAAGTTAACTTCAAGATCAGACATAACATCGAAGATGGTTCTGTTCAATTGGCAGATCATTACCAACAAAACACTCCAATTGGTGACGGTCCAGTTTTGTTGCCAGATAACCATTACTTGTCTACACAATCAGCATTGTCTAAAGATCCAAACGAAAAGAGAGATCACATGGTTTTGTTAGAATTTGTTACTGCTGCAGGTATTACATTGGGTATGGATGAATTATACAAA**TGA**GTTGTATCTGTACAAAATCCAAAGCTGAGCAAATAAATAAATAAATAAATGTATAAGTTACCGAACGGGGGTATTTTTACTTTTGATCAAAAATTTATGTACCAACTACAAAGTTTCCTCAGCACAGCCTTCAAGAAGGGAACACACATACAAACAGTGTCAAATAATTGTAGGGATAAATTTAAATATGGCATAAACTAAATAAGTAGAGCATGAAAAAACTGCAAAATCCAAAAAGTAAAAACGAAGGTCAGAAAGTAAAGCAAAAGAAAATTAATAAAGCAATACTAAATCTATCATGATTTCCCGTAACTTCCATTAAAGCTGTAACCAGATTTACTCCTACTGTTTGAGCCTCTAACGCCTAATGGATTTTTAGAGAAGCTCAACCTGATACCTCCTTTGTTGTTGAGGGAAGGGCGGGGGTGAGGTAGTTGACTACCATATAATTCTGCCAATGCTCTAGTGGCAAAGCTAACATCCTCATCAGTACTGACAATAAAAAGATTCTTGTTTTCAAGAACTTGTCATTTGTATAGTTTTTTTATATTGTAGTTGTTCTATTTTAATCAAATGTTAGCGTGATTTATATTTTTTTTCGCCTCGACATCATCTGCCCAGATGCGAAGTTAAGTGCGCAGAAAGTAATATCATGCGTCAATCGTATGTGAATGCTGGTCGCTATACTGCTGTCGATTCGATACTAACGCCGCCATCCAGT

**NotI-TAP(link)-ymUkG1(opt)-STOP-SHS1(term)-MX(term):**

pGF-TOPO+IVL1380

**GCGGCCGCGGTCGACGGATCCCCGGGTTAATTAATCCAATG**GTTTCTGTTATTAAAGAAGAAATGAAAATTAAATTGCACATGGAGGGTAACGTTAACGGTCATGCTTTCGTTATCGAAGGTGACGGTAAAGGTAAACCATACGATGGTACTCAAACATTGAATTTGACTGTTAAGGAAGGTGCTCCATTACCATTTTCTTATGATATTTTGACTAACGCATTCCAATACGGTAACAGAGCTTTTACAAAATATCCAGCAGATATTCCAGATTACTTCAAGCAAACTTTCCCAGAAGGTTACTCTTGGGAAAGAACAATGTCATACGAAGATAACGCTATTTGTAACGTTAGATCTGAAATTTCAATGGAAGGTGACTGTTTCATCTATAAGATCAGATTCGATGGTAAAAATTTTCCACCAAATGGTCCAGTTATGCAAAAGAAAACTTTGAAGTGGGAACCATCTACAGAAATGATGTATGTTAGAGATGGTTTCTTGATGGGTGACGTTAATATGGCATTGTTGTTGGAAGGTGGTGGTCATCATAGATGTGATTTCAAGACATCATACAAGGCTAAGAAAGTTGTTCAATTGCCAGATGCACATAAGATCGATCATAGAATCGAAATCTTGTCTCATGATAGAGATTATTCAAAAGTTAAATTGTACGAAAATGCTGTTGCAAGAAATTCTTTATTGCCATCTCAAGCATCAAAA**TGA**GTTGTATCTGTACAAAATCCAAAGCTGAGCAAATAAATAAATAAATAAATGTATAAGTTACCGAACGGGGGTATTTTTACTTTTGATCAAAAATTTATGTACCAACTACAAAGTTTCCTCAGCACAGCCTTCAAGAAGGGAACACACATACAAACAGTGTCAAATAATTGTAGGGATAAATTTAAATATGGCATAAACTAAATAAGTAGAGCATGAAAAAACTGCAAAATCCAAAAAGTAAAAACGAAGGTCAGAAAGTAAAGCAAAAGAAAATTAATAAAGCAATACTAAATCTATCATGATTTCCCGTAACTTCCATTAAAGCTGTAACCAGATTTACTCCTACTGTTTGAGCCTCTAACGCCTAATGGATTTTTAGAGAAGCTCAACCTGATACCTCCTTTGTTGTTGAGGGAAGGGCGGGGGTGAGGTAGTTGACTACCATATAATTCTGCCAATGCTCTAGTGGCAAAGCTAACATCCTCATCAGTACTGACAATAAAAAGATTCTTGTTTTCAAGAACTTGTCATTTGTATAGTTTTTTTATATTGTAGTTGTTCTATTTTAATCAAATGTTAGCGTGATTTATATTTTTTTTCGCCTCGACATCATCTGCCCAGATGCGAAGTTAAGTGCGCAGAAAGTAATATCATGCGTCAATCGTATGTGAATGCTGGTCGCTATACTGCTGTCGATTCGATACTAACGCCGCCATCCAGT

**NotI-TAP(link)-eGFP(opt)-Lact-C2-STOP-SHS1(term)-MX(term):**

pGF-TOPO+IVL1381

**GCGGCCGCGGTCGACGGATCCCCGGGTTAATTAATCCA**agt**aaGggTgaGgaGTtGttTactgg**TGTTGTTCCAATTTTGGTTGAATTAGATGGTGACGTTAATGGTCATAAATTTTCTGTTTCAGGTGAAGGTGAAGGTGACGCTACTTATGGTAAATTGACATTGAAGTTTATTTGTACTACTGGTAAATTGCCAGTTCCTTGGCCAACTTTGGTTACTACATTGACTTATGGTGTTCAATGTTTTTCAAGATACCCAGATCACATGAAGCAACATGATTTCTTTAAGTCTGCTATGCCAGAAGGTTACGTTCAAGAAAGAACAATTTTCTTTAAAGATGATGGTAACTACAAGACTAGAGCAGAGGTTAAGTTCGAAGGTGACACATTGGTTAACAGAATTGAATTAAAAGGTATTGATTTTAAAGAAGATGGTAATATTTTGGGTCATAAATTAGAATACAACTACAACTCACATAATGTTTATATTATGGCTGATAAACAAAAGAATGGTATTAAAGTTAACTTCAAGATCAGACATAACATCGAAGATGGTTCTGTTCAATTGGCAGATCATTACCAACAAAACACTCCAATTGGTGACGGTCCAGTTTTGTTGCCAGATAACCATTACTTGTCTACACAATCAGCATTGTCTAAAGATCCAAACGAAAAGAGAGATCACATGGTTTTGTTAGAATTTGTTACTGCTGCAGGTATTACATTGGGTATGGATGAATTATACAAATGCACTGAACCCCTAGGCCTGAAGGATAATACCATCCCCAACAAGCAGATCACAGCCTCCAGCTACTACAAAACCTGGGGCCTGAGTGCCTTTAGCTGGTTTCCCTACTACGCACGACTGGATAATCAGGGCAAGTTCAACGCCTGGACCGCCCAGACCAACAGTGCCTCTGAGTGGCTGCAGATTGACCTGGGCTCCCAGAAGCGAGTCACGGGCATCATCACCCAGGGTGCCCGAGACTTTGGCCACATTCAATATGTGGCTGCCTACAGGGTGGCCTATGGTGATGATGGTGTGACCTGGACTGAGTACAAGGACCCGGGGGCCTCAGAAAGCAAGATTTTCCCTGGTAACATGGACAATAATTCCCACAAGAAGAACATATTTGAGACGCCGTTCCAGGCTCGCTTCGTGCGGATCCAGCCCGTGGCCTGGCACAACCGTATCACCCTGCGAGTGGAGCTGCTGGGCTGT**TGA**GTTGTATCTGTACAAAATCCAAAGCTGAGCAAATAAATAAATAAATAAATGTATAAGTTACCGAACGGGGGTATTTTTACTTTTGATCAAAAATTTATGTACCAACTACAAAGTTTCCTCAGCACAGCCTTCAAGAAGGGAACACACATACAAACAGTGTCAAATAATTGTAGGGATAAATTTAAATATGGCATAAACTAAATAAGTAGAGCATGAAAAAACTGCAAAATCCAAAAAGTAAAAACGAAGGTCAGAAAGTAAAGCAAAAGAAAATTAATAAAGCAATACTAAATCTATCATGATTTCCCGTAACTTCCATTAAAGCTGTAACCAGATTTACTCCTACTGTTTGAGCCTCTAACGCCTAATGGATTTTTAGAGAAGCTCAACCTGATACCTCCTTTGTTGTTGAGGGAAGGGCGGGGGTGAGGTAGTTGACTACCATATAATTCTGCCAATGCTCTAGTGGCAAAGCTAACATCCTCATCAGTACTGACAATAAAAAGATTCTTGTTTTCAAGAACTTGTCATTTGTATAGTTTTTTTATATTGTAGTTGTTCTATTTTAATCAAATGTTAGCGTGATTTATATTTTTTTTCGCCTCGACATCATCTGCCCAGATGCGAAGTTAAGTGCGCAGAAAGTAATATCATGCGTCAATCGTATGTGAATGCTGGTCGCTATACTGCTGTCGATTCGATACTAACGCCGCCATCCAGT

**Supplemental Figure 3:** sgRNA target selection (TAP, Kan^R^, GFP, and mCherry).

**a) TAP-TAG sgRNA:**

GGTCGACGGATCCCCGGGTTAATTAATCCATGGAAGAGAAGATGGAAAAAGAATTTCATAGCCGTCTCAGCAGCCAACCGCTT**TAAGAAAATCTCATCCTCCGGGG**CACTTGATTATGATATTCCAACTACTGCTAGCGAGAATTTGTATTTTCAGGGAGAATTCGGCCTTGCGCAACACGATGAAGCCGTGGACAACAAATTCAACAAAGAACAACAAAACGCGTTCTATGAGATCTTACATTTACCTAACTTAAACGAAGAACAACGAAACGCCTTCATCCAAAGTTTAAAAGATGACCCAAGCCAAAGCGCTAACCTTTTAGCAGAAGCTAAAAAGCTAAATGATGCTCAGGCGCCGAAAGTAGACAACAAATTCAACAAAGAACAACAAAACGCGTTCTATGAGATCTTACATTTACCTAACTTAAACGAAGAACAACGAAACGCCTTCATCCAAAGTTTAAAAGATGACCCAAGCCAAAGCGCTAACCTTTTAGCAGAAGCTAAAAAGCTAAATGATGCTCAGGCGCCGAAAGTAGACGCGAATCATCAG**TGA**

**sgRNA+PAM nucleotide BLAST versus *Saccharomyces cerevisiae* S288C (taxid:559292)**

23 bp query = 15/17 closest match

15 bp (seed) query = 12/13 closest match

**b) MX-Kan^R^-MX sgRNA (v1.0 and v2.0):**

GTTTAGCTTGCCTCGTCCCCGCCGGGTCACCCGGCCAGCGACATGGAGGCCCAGAATACCCTCCTTGACAGTCTTGACGTGCGCAGCTCAGGGGCATGATGTGACTGTCGCCCGTACATTTAGCCCATACATCCCCATGTATAATCATTTGCATCCATACATTTTGATGGCCGCACGGCGCGAAGCAAAAATTACGGCTCCTCGCTGCAGACCTGCGAGCAGGGAAACGCTCCCCTCACAGACGCGTTGAATTGTCCCCACGCCGCGCCCCTGTAGAGAAATATAAAAGGTTAGGATTTGCCACTGAGGTTCTTCTTTCATATACTTCCTTTTAAAATCTTGCTAGGATACAGTTCTCACATCACATCCGAACATAAACAACC**ATG**GGTAAGGAAAAGACTCACGTTTCGAGGCCGCGATTAAATTCCAACATGGATGCTGATTTATATGGGTAT**AAATGGGCTCGCGATAATGTCGG**GCAATCAGGTGCGACAATCTATCGATTGTATGGGAAGCCCGATGCGCCAGAGTTGTTTCTGAAACATGGCAAAGGTAGCGTTGCCAATGATGTTACAGATGAGATGGTCAGACTAAACTGGCTGACGGAATTTATGCCTCTTCCGACCATCAAGCATTTTATCCGTACTCCTGATGATGCATGGTTACTCACCACTGCGATCCCCGGCAAAACAGCATTCCAGGTATTAGAAGAATATCCTGATTCAGGTGAAAATATTGTTGATGCGCTGGCAGTGTTCCTGCGCCGGTTGCATTCGATTCCTGTTTGTAATTGTCCTTTTAACAGCGATCGCGTATTTCGTCTCGCTCAGGCGCAATCACGAATGAATAACGGTTTGGTTGATGCGAGTGATTTTGATGACGAGCGTAATGGCTGGCCTGTTGAACAAGTCTGGAAAGAAATGCATAAGCTTTTGCCATTCTCACCGGATTCAGTCGTCACTCATGGTGATTTCTCACTTGATAACCTTATTTTTGACGAGGGGAAATTAATAGGTTGTATTGATGTTGGACGAGTCGGAATCGCAGACCGATACCAGGATCTT**GCCATCCTATGGAACTGCCTCGG**TGAGTTTTCTCCTTCATTACAGAAACGGCTTTTTCAAAAATATGGTATTGATAATCCTGATATGAATAAATTGCAGTTTCATTTGATGCTCGATGAGTTTTTC**TAA**TCAGTACTGACAATAAAAAGATTCTTGTTTTCAAGAACTTGTCATTTGTATAGTTTTTTTATATTGTAGTTGTTCTATTTTAATCAAATGTTAGCGTGATTTATATTTTTTTTCGCCTCGACATCATCTGCCCAGATGCGAAGTTAAGTGCGCAGAAAGTAATATCATGCGTCAATCGTATGTGAATGCTGGTCGCTATACTGCTGTCGATTCGATACTAACGCCGCCATCCAGT

**sgRNA(Kan v1.0) = AAATGGGCTCGCGATAATGT**

**sgRNA(Kan v2.0) = GCCATCCTATGGAACTGCCT**

**sgRNA+PAM nucleotide BLAST versus *Saccharomyces cerevisiae* S288C (taxid:559292)**

[v1.0] 23 bp query = 13/14 closest match

[v1.0] 15 bp (seed) query = 12/12 closest match

[v2.0] 23 bp query = 16/18 closest match

[v2.0] 15 bp (seed) query = 12/12 and 12/13 closest matches

**c) GFP and mCherry sgRNA:**

**sgRNA(GFP):**

ggtcgacggatccccgggttaattaacagt**aaaggagaagaacttttcactgg**agttgtcccaattcttgttgaattagatggtgatgttaatgggcacaaattttctgtcagtggAgagggtgaaggtgatgcaacatacggaaaacttacccttaaatttatttgcactactggaaaactacctgttccatggccaacacttgtcactactttgacttatggtgttcaatgcttttcaagatacccagatcatatgaaacggcatgactttttcaagagtgccatgcccgaaggttatgtacaggaaagaactatatttttcaaagatgacgggaactacaagacacgtgctgaagtcaagtttgaaggtgatacccttgttaatagaatcgagttaaaaggtattgattttaaagaagatggaaacattcttggacacaaattggaatacaactataactcacacaatgtatacatcatggcagacaaacaaaagaatggaatcaaagttaacttcaaaattagacacaacattgaagatggaagcgttcaactagcagaccattatcaacaaaatactccaattggcgatggccctgtccttttaccagacaaccattacctgtccacacaatctgccctttcgaaagatcccaacgaaaagagagaccacatggtccttcttgagtttgtaacagctgctgggattacacatggcatggatgaactatacaaa**TAG**

**sgRNA+PAM nucleotide BLAST versus *Saccharomyces cerevisiae* S288C (taxid:559292)**

23 bp query = 16/17 closest match

15 bp (seed) query = 13/13 closest match

**sgRNA(mCherry):**

gtgagcaagggcgaggaggataacatggccatcat**caaggagttcatgcgcttcaagg**tgcacatggagggctccgtgaacggccacgagttcgagatcgagggcgagggcgagggccgcccctacgagggcacccagaccgccaagctgaaggtgaccaagggtggccccctgcccttcgcctgggacatcctgtcccctcagttcatgtacggctccaaggcctacgtgaagcaccccgccgacatccccgactacttgaagctgtccttccccgagggcttcaagtgggagcgcgtgatgaacttcgaggacggcggcgtggtgaccgtgacccaggactcctccctgcaggacggcgagttcatctacaaggtgaagctgcgcggcaccaacttcccctccgacggccccgtaatgcagaagaagaccatgggctgggaggcctcctccgagcggatgtaccccgaggacggcgccctgaagggcgagatcaagcagaggctgaagctgaaggacggcggccactacgacgctgaggtcaagaccacctacaaggccaagaagcccgtgcagctgcccggcgcctacaacgtcaacatcaagttggacatcacctcccacaacgaggactacaccatcgtggaacagtacgaacgcgccgagggccgccactccaccggcggcatggacgagctgtacaag**TAA**

**sgRNA+PAM nucleotide BLAST versus *Saccharomyces cerevisiae* S288C (taxid:559292)**

23 bp query = 16/16 closest match

15 bp (seed) query = 12/12 closest match

**Supplemental Figure 4:** Analysis of marker-less integrations for *KEL1, BUD3,* and *ELM1*.

For Cas9-based integrations of 18 C-terminal fusions (Fig. 3) at either the *KEL1*, *BUD3*, or *ELM1* loci (from the TAP-tag collection), colonies growing on SD-URA-LEU plates (containing both the Cas9 and sgRNA plasmids) were randomly selected, and between 50-100 were tested on SD-HIS medium. The percentage of isolates sensitive (dead) on SD-HIS plates was quantified for each integration event. Asterisk, proper in-frame fusion of the *S. pombe* His5 (equivalent to yeast His3) gene product may (or may not) allow for a cytosolic-exposed His5 protein. Therefore, proper integrants could not be screened on SD-HIS plates for this C-terminal fusion. Kel1 and Bud3 differed from Elm1 fusions to *S. pombe* His5 in this respect. For each integration event, an initial screening on SD-HIS plates ensured proper loss/replacement of the endogenous marker. Of those clonal isolates that had *lost* the chromosomal marker, only one (or rarely, a second) colony was assayed by diagnostic PCR and (when appropriate), DNA sequencing. Our analysis of the proportion of surviving colonies on SD-HIS medium demonstrates that across 17 (*sans S. pombe* His5) separate donor DNAs for 3 separate loci, between 75-90% of randomly selected isolates have lost the endogenous selection marker and (very likely) have properly integrated the intended C-terminal tag. Should this methodology be applied in a high-throughput fashion, we estimate that only a very small number of colonies need to be screened on SD-HIS, if at all, prior to additional low-throughput screening.

**Supplemental Figure 5:** Mating and Diploid selection for Cas9-based gene drives.


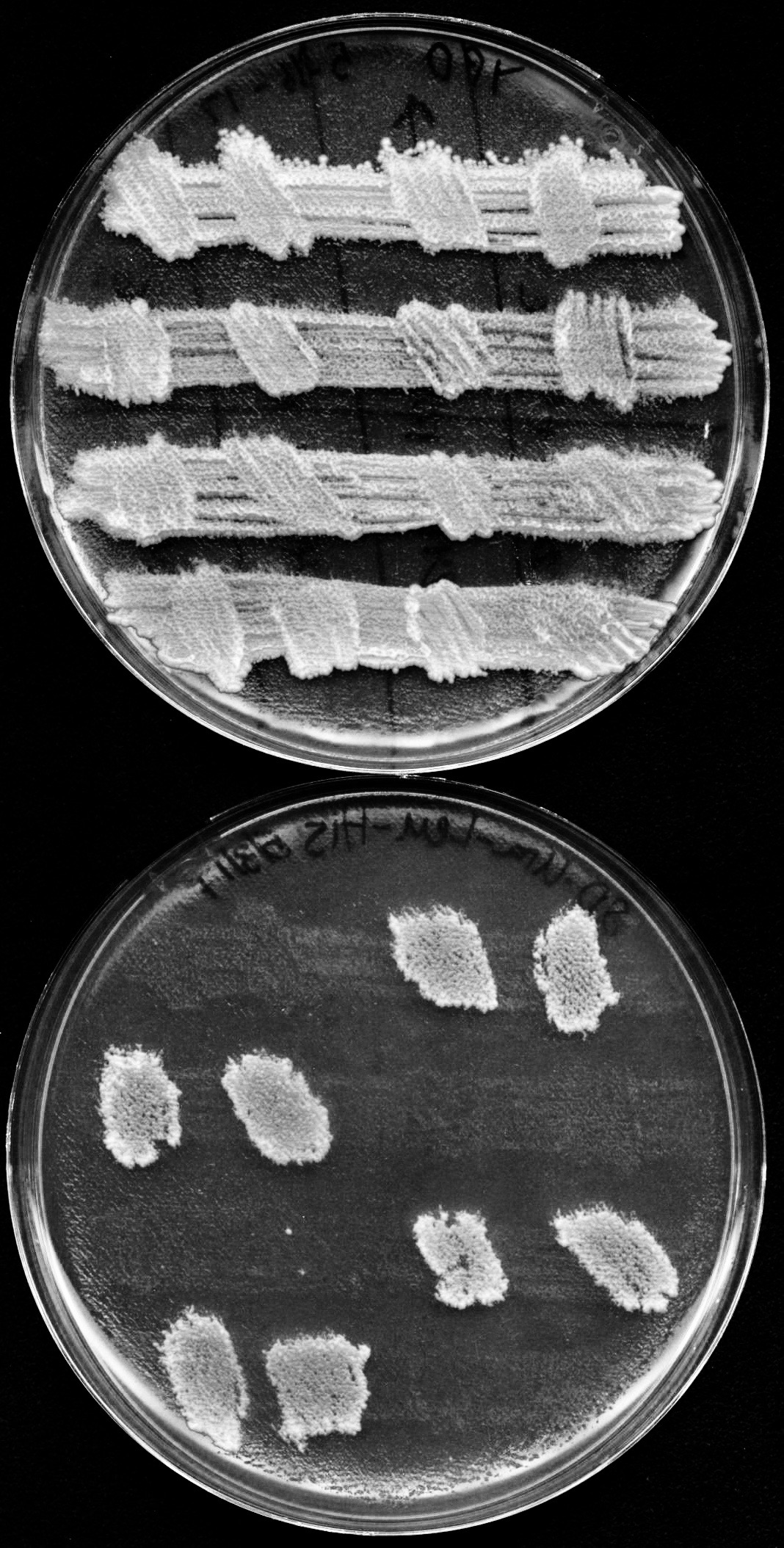

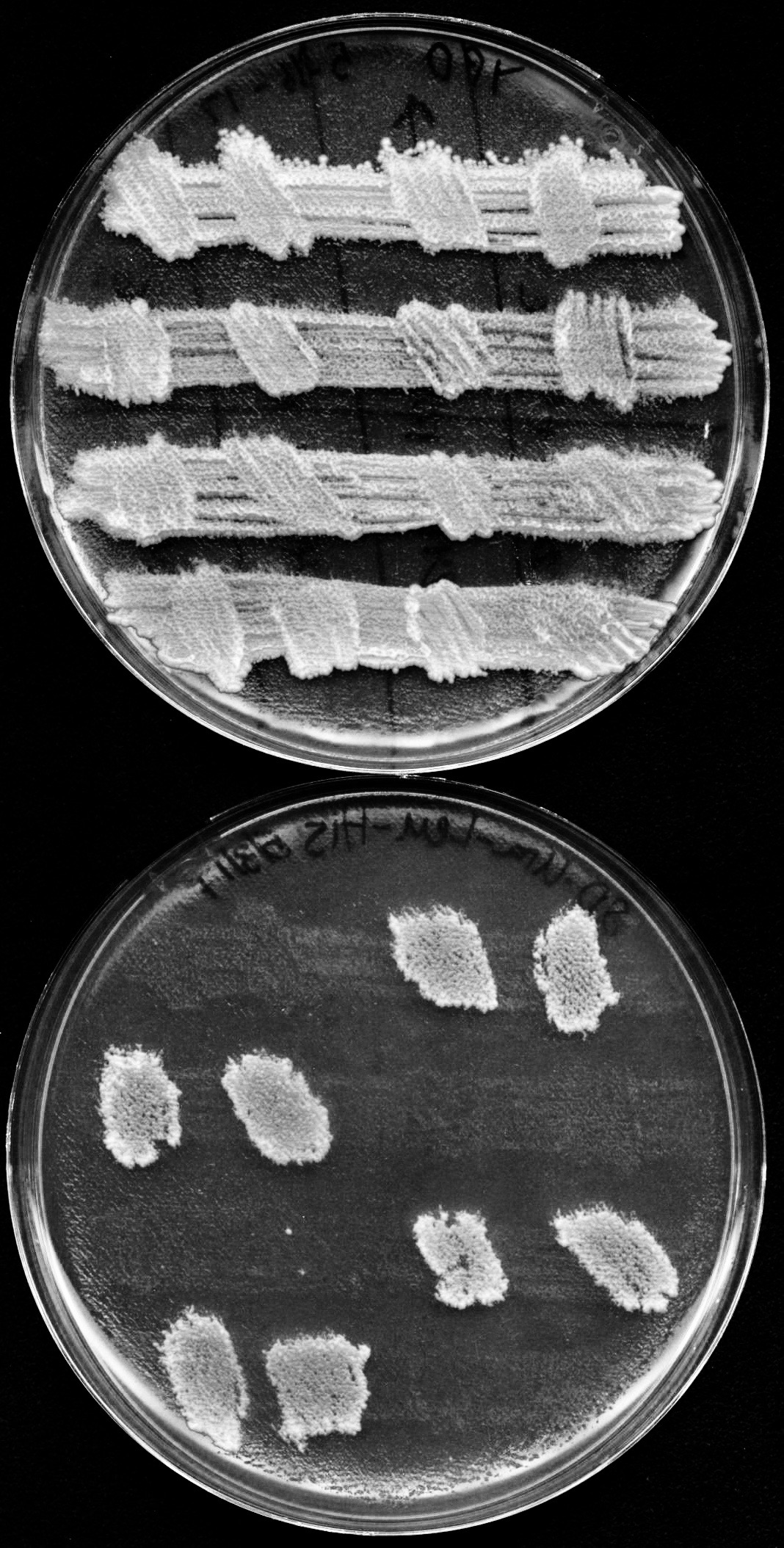


YP+Dex

SD-URA-LEU-HIS

1

2

1

2

1 = Target *MAT****⍺*** (GFY-2624)

2 = Target *MAT****a*** (GFY-2625)

A

B

C

D

A = Gene Drive *MAT****⍺*** (GFY-2442) + sgRNA

B = Gene Drive *MAT****⍺*** (GFY-2442) + empty vector

C = Gene Drive *MAT****a*** (GFY-2440) + sgRNA

D = Gene Drive *MAT****a*** (GFY-2440) + empty vector

**Mating**

**Diploids**

Yeast from either the “target” strain or the “gene drive” strain of both yeast mating types were mated together and diploids were selected. Only the proper combinations of *MAT****a*** x *MAT****⍺*** resulted in viable diploids. The sgRNA-expressing plasmid and the empty vector used (pRS425) both have LEU2 selection. Both the target and gene drive strains also harbor *URA3*-based covering vectors (expressing WT *CDC11*). The target yeast genome is marked with *SpHIS5.* A velvet cloth was used to “replica-plate” the yeast from the mating YPD plate to the diploid selection plate.

**Supplemental Figure 6:** Fluorescence microscopy of haploid yeast for “gene drive” and “target” strains.


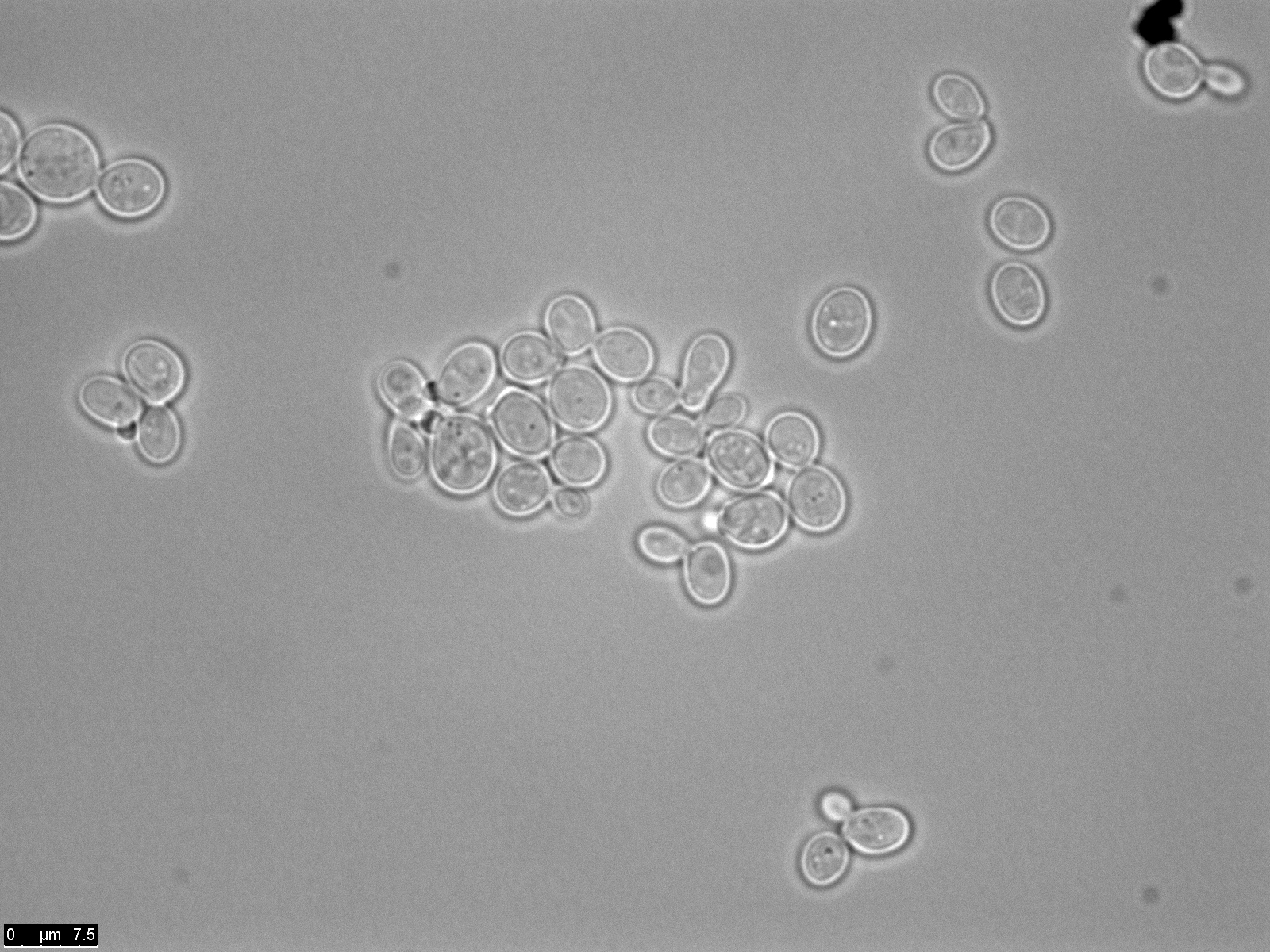

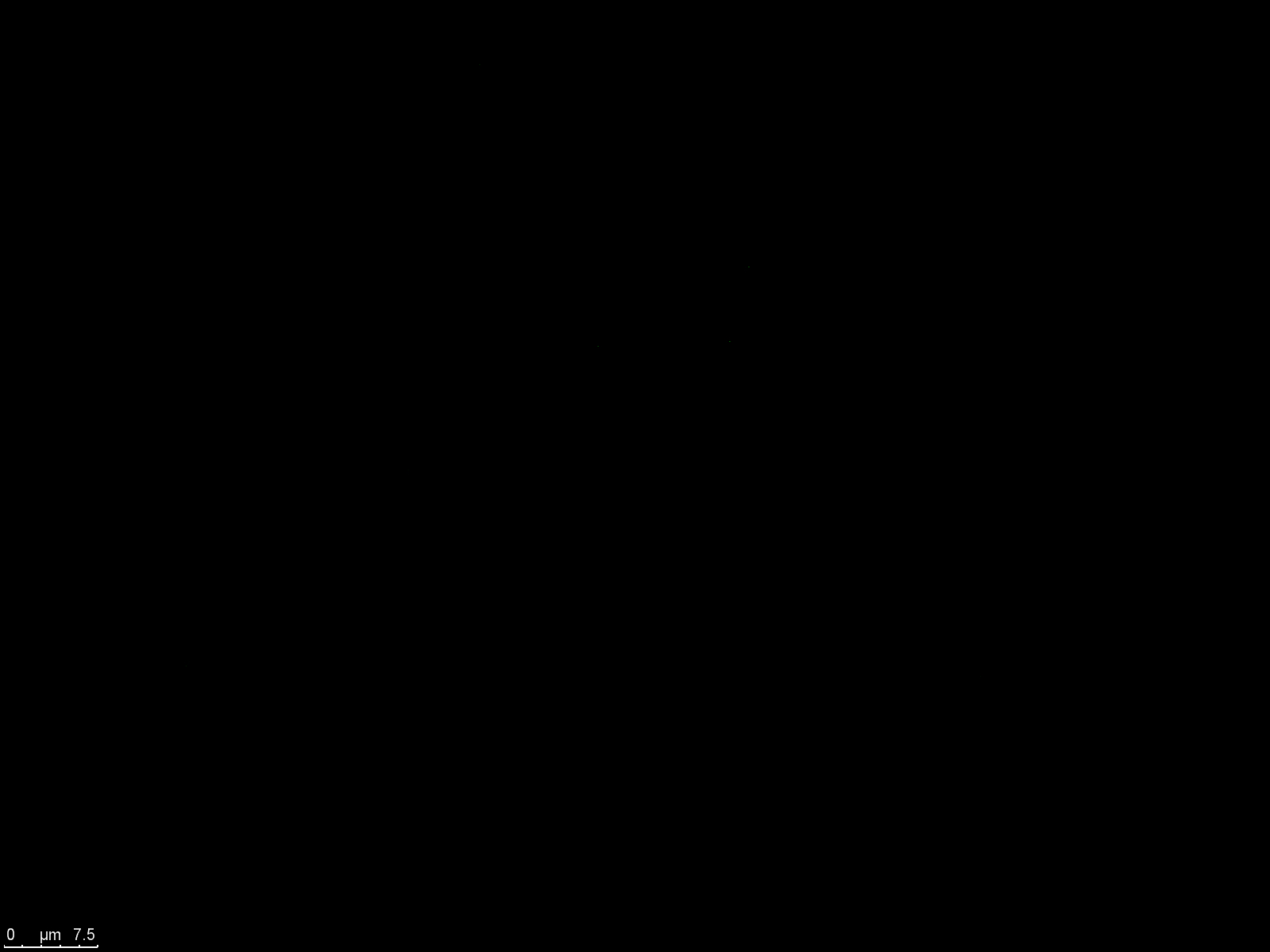

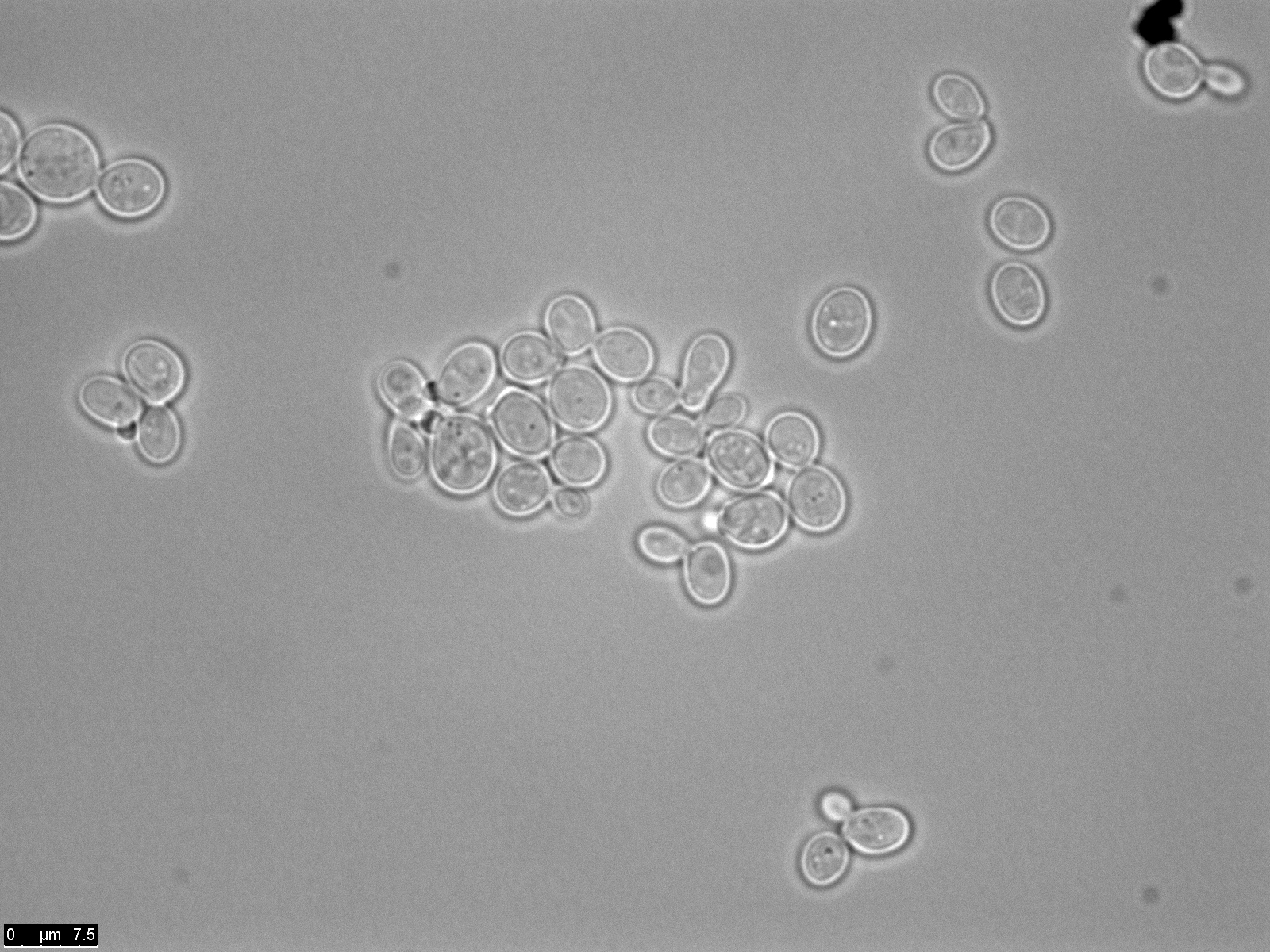

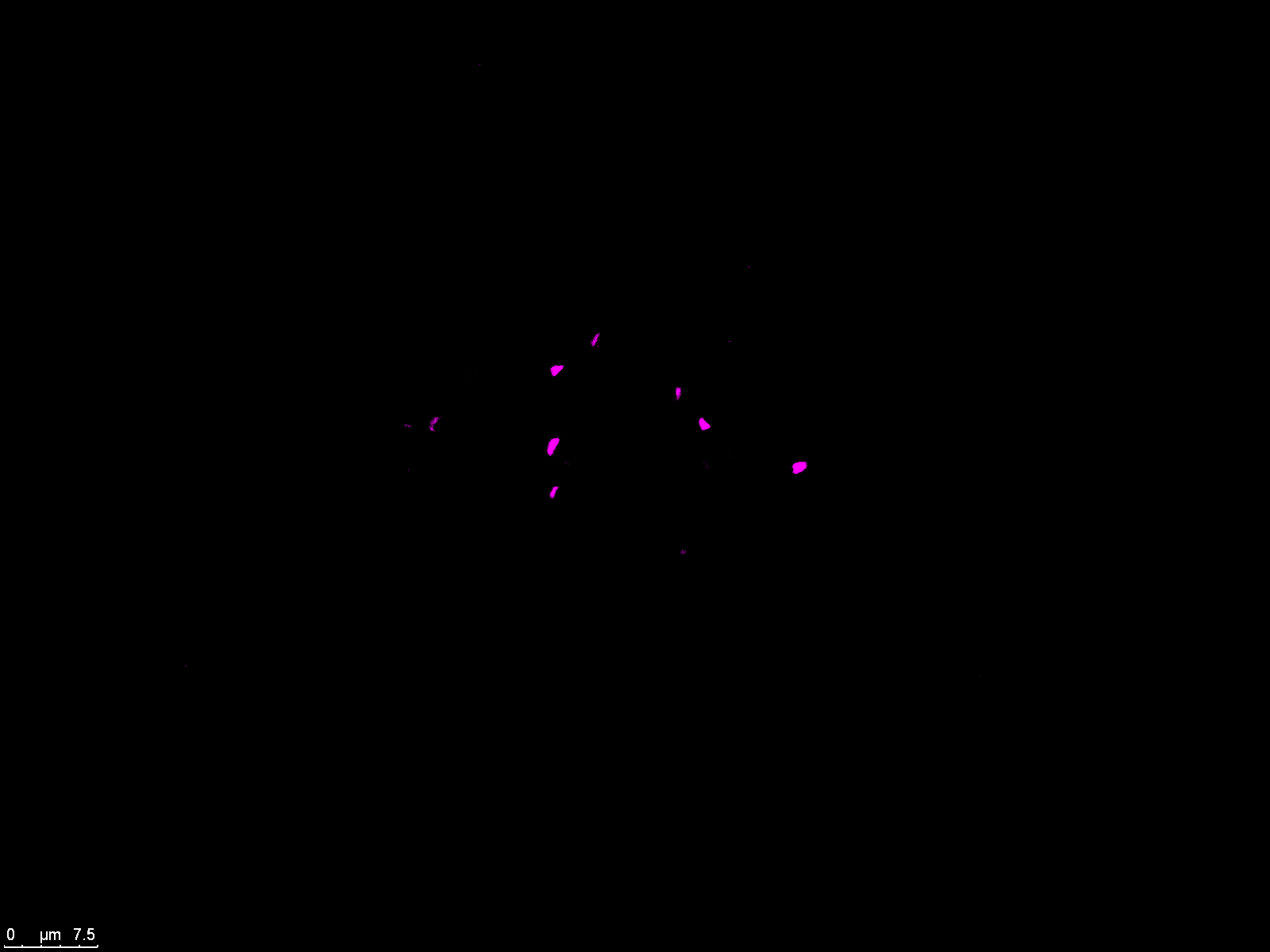

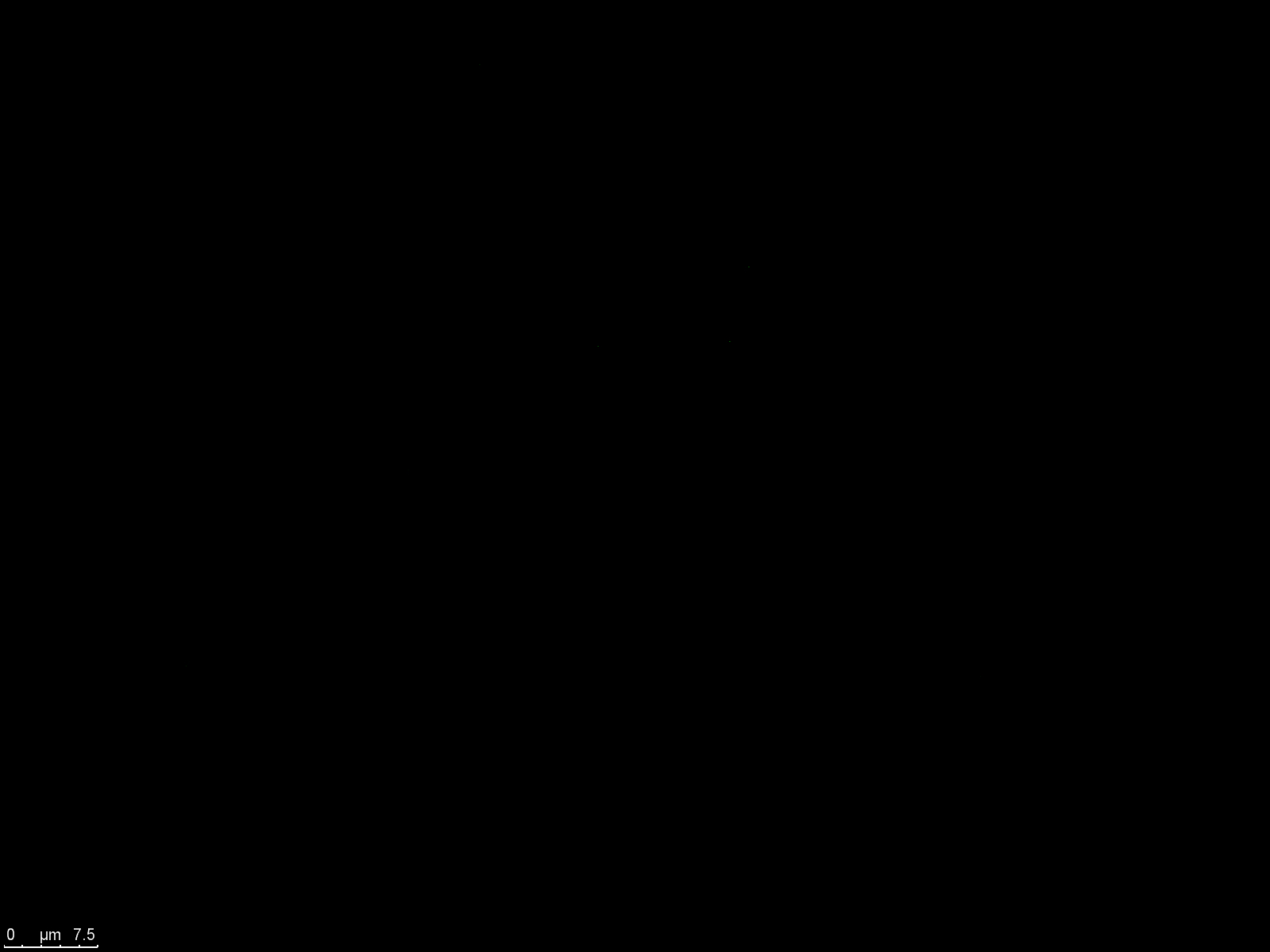

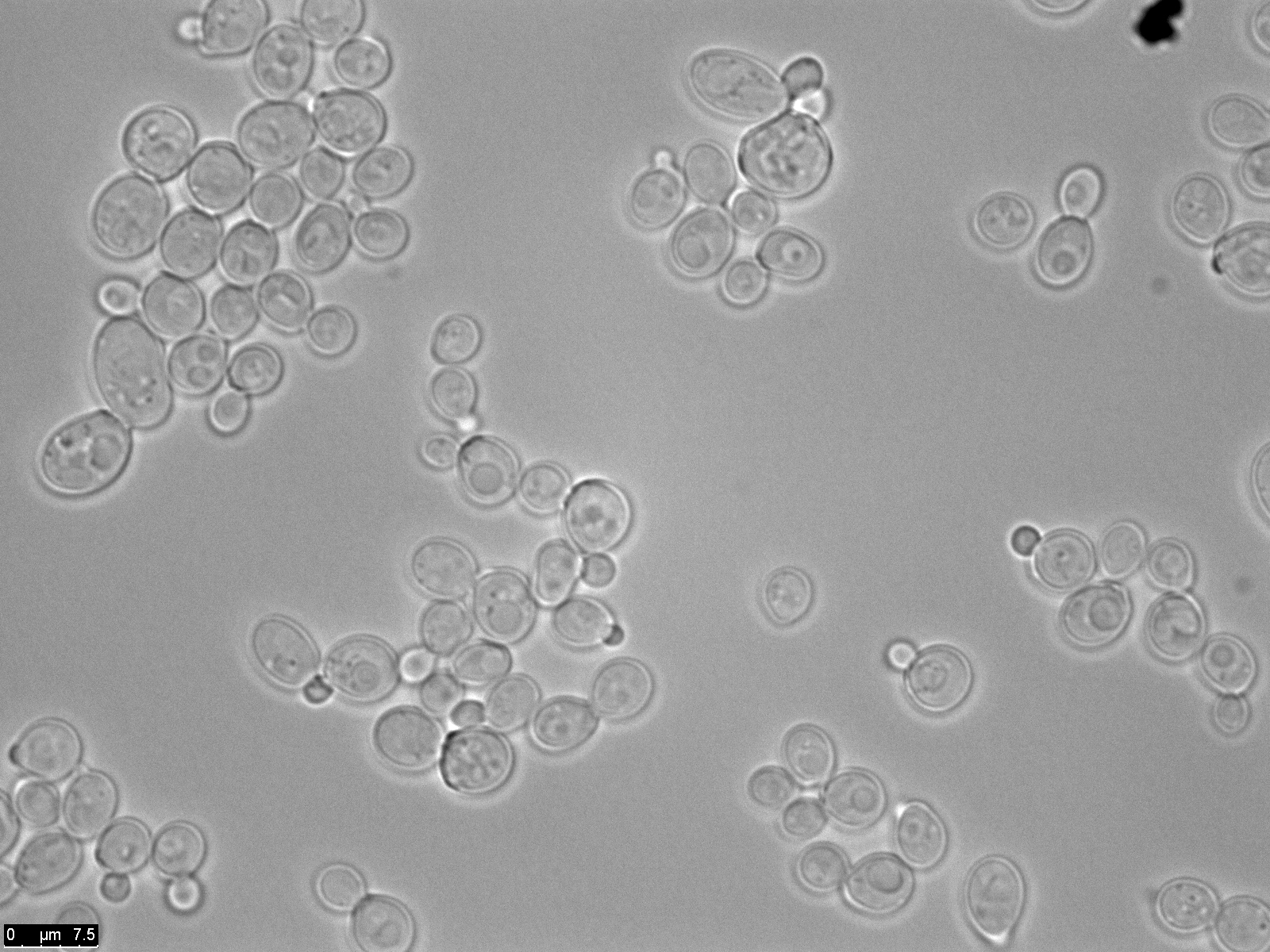

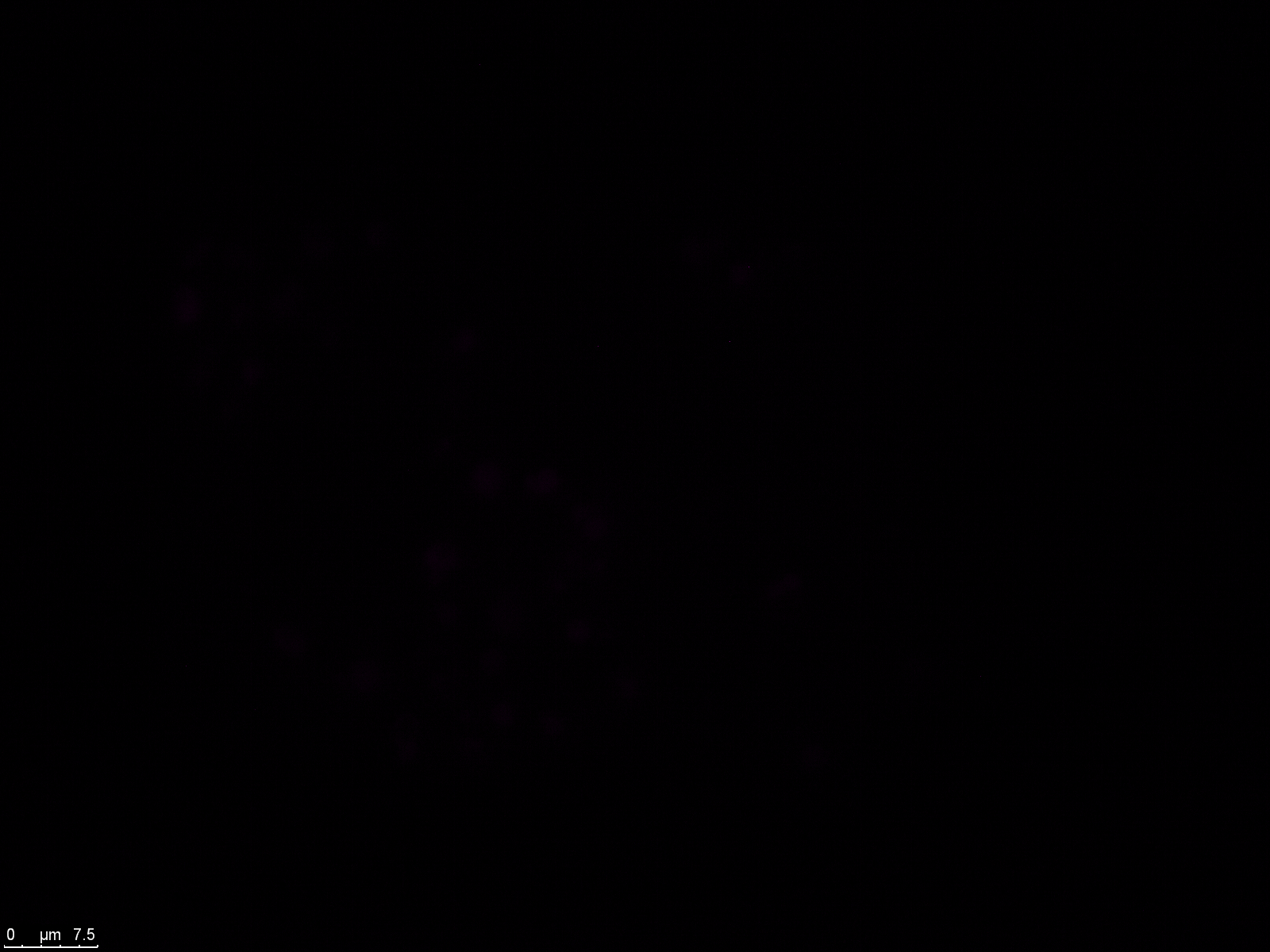

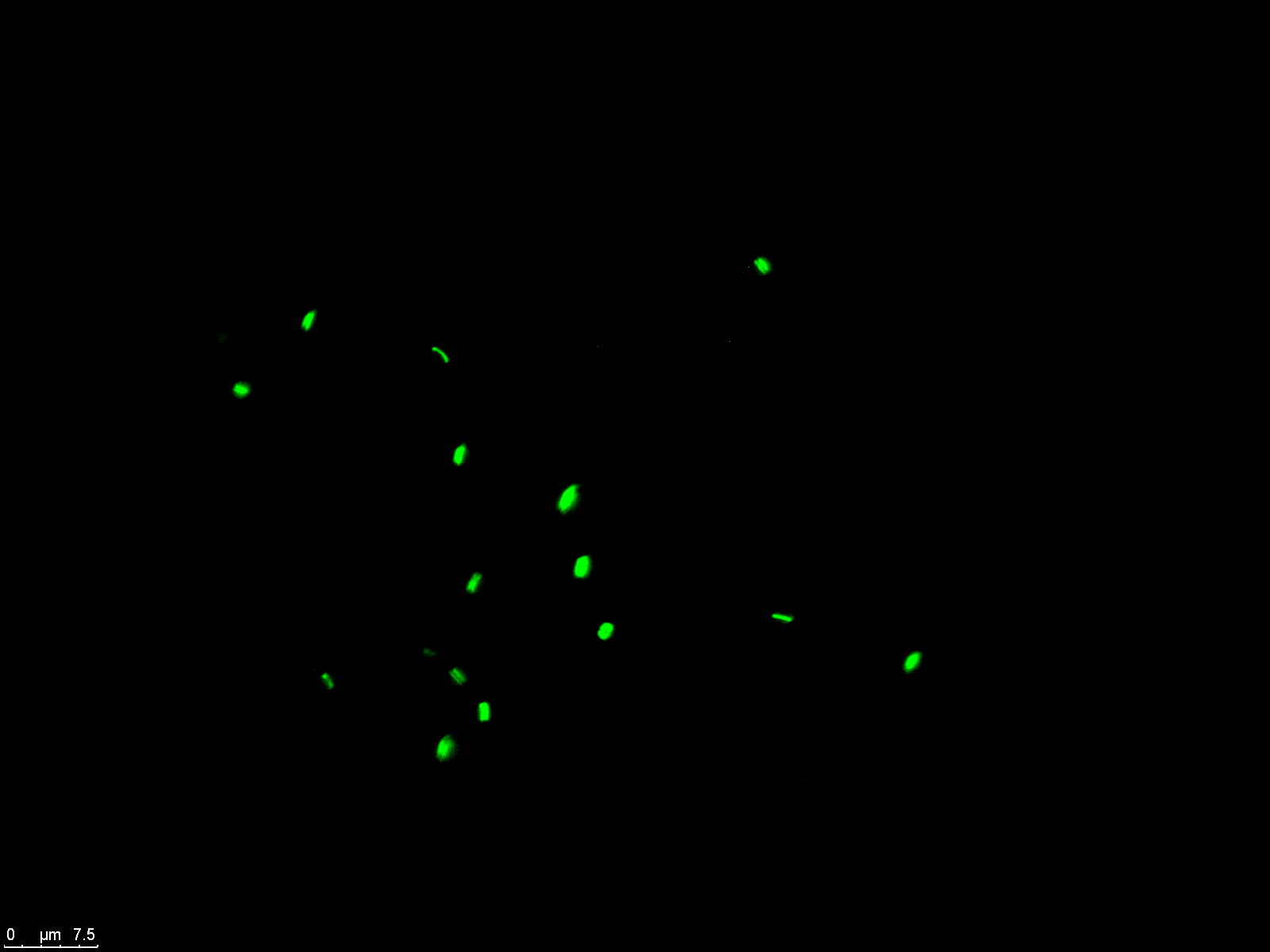


DIC

GFP

mCherry

GFY-2624

[CDC11-GFP]

GFY-2440

[cdc11-CTE∆-mCherry]

[Both strains may also express an untagged WT copy of Cdc11]

Haploid yeast strains (GFY-2624 and GFY-2440) were cultured to saturation overnight at 30°C in SD-URA (to maintain the presence of the WT *CDC11-*expressing covering plasmid) and back-diluted into YPDex liquid medium for approximately 4 hours. Cells were harvested, centrifuged, and washed with water before transfering to a glass coverslip for imaging. Images for each channel were taken at identical exposure times, adjusted using ImageJ, and resized accordingly. Dotted white lines, cell periphery. Scale bar, 3 μm. The haploid starting strains have a single fluorescently-tagged Cdc11 septin at the bud neck (GFP or mCherry). Since the presence of the cdc11-CTE∆-mCherry mutant (residues 357-415∆) can cause an elongated cell phenotype (Finnigan *et al.* 2015), both strains were grown in the presence of the WT covering plasmid (untagged).

**Table S1:** Oligonucleotides used in this study.

| **Name** | **DNA Sequence (5’ to 3’)** |
| --- | --- |
| TAP Tag clone out F^1^ | GGTCGACGGATCCCCGGGTTAATTAATC |
| MX clone out R2^1^ | ACTGGATGGCGGCGTTAGTATCGAATCG |
| *KEL1* Internal +2908 F^2^ | CGGATGAAAATGGCGAGAAAACTGTCGGT |
| *BUD3* Internal +4381 F | CAAGAGCACAGACGATAAGTTGAGTAGCG |
| *ELM1* Internal +1455 F | CCGGATAAAGAATGTTTTTCGACTACGGTC |
| Internal *ADH1*(t) R | CCTACAGGAAAGAGTTACTCAAGAATAAGAATTTTC |
| *CAF120* Internal F | CCAACCTTATGCGATGAATACGCACATGG |
| *NBA1* Internal F | CTACTTCGACAAACAAACAAGATGATGACATGTAC |
| *BEM2* Internal F | CCGATTTGGCGATTAAGTATAAAGCACATGC |
| *MYO4* Internal F | CGATTTCTGGTATGCCTTGAATCCAGCCC |
| *BUD2* Internal F | CTCTTCCGCAAGATTGCAGAAATTCTTCAC |
| *SHS1*(t) -192 R | GCCATATTTAAATTTATCCCTACAATTATTTGACACTGTTTG |
| eGFP clone out F^3^ | GGTCGACGGATCCCCGGGTTAATTAACA |
| mCherry clone out F | GTGAGCAAGGGCGAGGAGGATAACATG |
| Internal mCherry(opt) F | GGGAAAGAGTTATGAACTTCGAAGATGGTG |
| Internal mCherry(opt) R | GCCTTGTATGTAGTCTTAACTTCAGCATCG |
| prCDC12 +276 F | GATGGGACATGATGCAGTATCACGATTAGCAA |
| prSHS1 +261 F | CCGCGATAAAATTGCTCAATTGGCACCATTTAAAC |
| CDC10(term) -201 R | CAAACGAGAAGGTGATAGCTGTACGCCAG |
| HIS3 +196 F | GGCCTCCTCTAGTACACTCTATATTTTTTTATGC |
| HIS3 -151 R | CGCCTCGTTCAGAATGACACGTATAGAATG |
| pr*CDC11* +330 F | CACAACATGGAACATAACATTTAAACATCGTTCTCAATC |
| MXpr clone out F^1^ | GTTTAGCTTGCCTCGTCCCCGCCG |
| *MSO1* clone out F | ATGAGTCAAGTATCGCATTCCCAAGAAGG |
| *APL1* clone out F | TCCGATCAAAAAGTTTTTGCCAGATATAAAGCAAATG |
| *APL1* Internal +305 R | AAATCGTCCATTATAAATGGCAAAGCCTCTCTG |
| *APL1* Internal +1751 F | GATCCCACCGTTTTGGAGGAATTGGAGCT |
| *BNI4* clone out F | TCGGATAGTATTTCAGATTCAAAGTCCTCAGAAC |
| *BNI4* Internal +2312 F | CATAGAACAGCCCATAGAAGTTACTCCAAG |
| GFP(β10)-Link-*BNI4* R | AGGACTTTGAATCTGAAATACTATCCGACATCTTTTTGGAACCACCA  CCTGCGGAACCAC |
| GFP(β10)-Link-*APL1* R | TATATCTGGCAAAAACTTTTTGATCGGACATCTTTTTGGAACCACCA  CCTGCGGAACCAC |
| GFP(β10)-Link-*MSO1* R | CTTCTTGGGAATGCGATACTTGACTCATCATCTTTTTGGAACCACCA  CCTGCGGAACCAC |
| *CDC11* +406 F | CATCTACAAAAGCAGGTTATAGCTCCGTTAAAC |
| *CDC11* -327 R | GCTAAGTGATGTTCTGGTCTTTCCAAAATTCTC |
| pr*GAL* +192 F | GGGGTAATTAATCAGCGAAGCGATGATTTTTG |
| SpCas9 Int +373 R | CATCAACGATGTTACCGAAGATTGGATGTC |
| SpCas9 Int +3653 F | CGGTAGAAAAAGAATGTTAGCTTCAGCTGG |
| Int Kan F | GTCCTTTTAACAGCGATCGCGTATTTCGTC |
| Int *SpHIS5* F1 | GGGAGAACAAGTAATCCAAGTAGACACGGG |
| Int GFP R new | CCCGTCATCTTTGAAAAATATAGTTCTTTCCTG |

^1^These are universal primers that allow for amplification of constructed cassettes.

^2^These are unique primers to a single gene target.

^3^The eGFP clone out F primer is also used to amplify the ymUkG1-containing cassette.

**Table S2:** Expected DNA fragment sizes (bp) for diagnostic PCRs corresponding to Fig. 3C.

| **PCR** | **Marker Type** | ***KEL1*** | ***BUD3*** | ***ELM1*** |
| --- | --- | --- | --- | --- |
| A | 1xFLAG-Link(25)-GFP(**β**11) | 1001 | 943 | 882 |
| B | 1xFLAG-Link(25)-SpeI-6xHIS | 962 | 904 | 843 |
| C | mCherry(opt) | 1544 | 1486 | 1425 |
| D | anti-GFP Nanobody | 1190 | 1132 | 1071 |
| E | GST | 1514 | 1456 | 1395 |
| F | 1xFLAG-Link(25)-3xHA | 1034 | 976 | 915 |
| G | NLS | 872 | 814 | 753 |
| H | NES | 872 | 814 | 753 |
| I | 1xMYC | 872 | 814 | 753 |
| J | MBP | 1964 | 1906 | 1845 |
| K | BirA(R118G) | 1802 | 1744 | 1683 |
| L | CAAX Box Motif | 866 | 808 | 747 |
| M | SNAP tag | 1385 | 1327 | 1266 |
| N | *S. pombe* His5 | 1487 | 1429 | 1368 |
| O | mScarlet(opt) | 1532 | 1474 | 1413 |
| P | eGFP(opt) | 1550 | 1492 | 1431 |
| Q | ymUkG1 (opt) | 1520 | 1462 | 1401 |
| R | eGFP(opt)-LactC2 | 2024 | 1966 | 1905 |

Diagnostic PCRs were formed on isolated chromosomal DNA from putative clonal isolates following Cas9-dependent integration into the yeast genome in place of the TAP tag (Fig. 3). A constant internal oligonucleotide (“forward”) to either the *KEL1*, *BUD3*, or *ELM1* open reading frame (see Table S1) was paired with an internal DNA primer (“reverse”) to the common *SHS1* terminator sequence. While the relative sizes (bp) of each expected PCR fragment is reported (Fig. 3), here we provide the exact expected values (bp) for all three sets (54) of separate PCR reactions using these C-terminal marker donor DNAs.

**Table S3:** Ploidy confirmation tests of Cas9-gene drive isolates

| **Yeast strain** | **Tester Strain** | **Diploid Selection** | **Growth Results** |
| --- | --- | --- | --- |
| *MAT****a*** [GFY-2440]^1^ | *MAT****α;*** *cdc11∆Kan^R^n+ pCDC11::URA3* + pRS313 (empty) [GFY-150] | SD-LEU-HIS | Alive |
| *MAT****a*** [GFY-2440]^1^ | *MAT****a;*** *cdc11∆Kan^R^n+ pCDC11::URA3* + pRS313 (empty) [GFY-153] | SD-LEU-HIS | Dead |
| *MAT****α*** [GFY-2442]^1^ | *MAT****α;*** *cdc11∆Kan^R^n+ pCDC11::URA3* + pRS313 (empty) [GFY-150] | SD-LEU-HIS | Dead |
| *MAT****α*** [GFY-2442]^1^ | *MAT****a;*** *cdc11∆Kan^R^n+ pCDC11::URA3* + pRS313 (empty) [GFY-153] | SD-LEU-HIS | Alive |
| [GFY-2624] x [GFY-2440]^2,3^ +sgRNA, isolates 1-11 (HIS-) | *MAT****α;*** *cdc11∆Kan^R^n+ pCDC11::URA3* + pRS313 (empty) [GFY-150] | SD-LEU-HIS | Dead |
| [GFY-2624] x [GFY-2440]^2,3^ +sgRNA, isolates 1-11 (HIS-) | *MAT****a;*** *cdc11∆Kan^R^n+ pCDC11::URA3* + pRS313 (empty) [GFY-153] | SD-LEU-HIS | Dead |
| [GFY-2624] x [GFY-2440]^2,4^ +empty pRS425, isolates 1-8 | *MAT****α;*** *cdc11∆Kan^R^n+ pCDC11::URA3* + pRS313 (empty) [GFY-150] | SD-URA-LEU | Dead |
| [GFY-2624] x [GFY-2440]^2,4^ +empty pRS425, isolates 1-8 | *MAT****a;*** *cdc11∆Kan^R^n+ pCDC11::URA3* + pRS313 (empty) [GFY-153] | SD-URA-LEU | Dead |
| [GFY-2624] x [GFY-2440]^2,4^ +sgRNA, isolates 1-4 (HIS+)^5^ | *MAT****α;*** *cdc11∆Kan^R^n+ pCDC11::URA3* + pRS313 (empty) [GFY-150] | SD-URA-LEU | Dead |
| [GFY-2624] x [GFY-2440]^2,4^ +sgRNA, isolates 1-4 (HIS+)^5^ | *MAT****a;*** *cdc11∆Kan^R^n+ pCDC11::URA3* + pRS313 (empty) [GFY-153] | SD-URA-LEU | Dead |

^1^Haploid parental control strain

^2^Diploid strains following activation of the gene drive (Fig. 6).

^3^Selection test for dipoids used the *HIS3*-marked pRS313 from each of the tester strains and the *LEU2*-marked sgRNA plasmid present in the strains following activation of the gene drive. 11 separate clonal isolates that were dead on SD-HIS plates (the great majority of all colonies) were tested.

^4^Prior to the ploidy tests, strains were selected on SD-LEU+5-FOA media two consecutive times at 30°C to remove all *URA3*-based plasmids. The selection test for diploids used the *URA3*-marked covering vector from the tester strains (*pCDC11::URA3*) and the *LEU2*-marked sgRNA plasmid in the strains following activation of the gene drive. 8 separate clonal isolates were tested.

^5^Isolates from the active gene drive that were viable on SD-HIS plates (<1% of total population) were also selected and clonal isolates were tested in the ploidy mating assay.

**Table S4:** Summary of CRISPR-UnLOCK yeast verifications by diagnostic PCR & DNA Sequencing

| **Figure (Experiment)** | Total Isolates Assayed by Diagnostic PCR | Correct Isolates by PCR | Isolate Assayed by DNA Sequencing |
| --- | --- | --- | --- |
| Fig. 1 (V1)^1^ | 30 | 2/30 | 2 |
| Fig. 1 (V2)^1^ | 24 | 10/24 | 2 |
| Fig. 1 (V3)^1^ | 24 | 18/24 | 2 |
| Fig. 2 (*KEL1*) | 1 | 1/1 | ND^2^ |
| Fig. 2 (*BUD3*) | 1 | 1/1 | ND |
| Fig. 2 (*ELM1*) | 1 | 1/1 | ND |
| Fig. 2 (*CAF120*) | 1 | 1/1 | ND |
| Fig. 2 (*NBA1*) | 1 | 1/1 | ND |
| Fig. 2 (*BEM2*) | 1 | 1/1 | ND |
| Fig. 2 (*MYO4*) | 1 | 1/1 | ND |
| Fig. 2 (*BUD2*) | 1 | 1/1 | ND |
| Fig. 2 (*EPO1*) | 1 | 0/1 | ND |
| Fig. 3 (*KEL1*) | 18 (1x18) | 18/18 | 18 (1x18) |
| Fig. 3 (*BUD3*) | 18 (1x18) | 18/18 | ND |
| Fig. 3 (*ELM1*) | 18 (1x18) | 18/18 | ND |
| Fig. 4 (pr*SHS1*-*MSO1*)^3^ | 1 | 1/1 | 1 |
| Fig. 4 (pr*CDC11*-*MSO1*)^3^ | 1 | 1/1 | 1 |
| Fig. 4 (pr*SHS1*-*APL1*) | 1 | 1/1 | 1 |
| Fig. 4 (pr*CDC11*-*APL1*) | 1 | 1/1 | 1 |
| Fig. 4 (pr*SHS1*-*BNI4*) | 1 | 1/1 | 1 |
| Fig. 4 (pr*CDC11*-*BNI4*) | 1 | 1/1 | 1 |
| Fig. 4 (37 other loci)^4^ | ND | ND | 37 |
| Fig. 5 (Strain 1+eGFP donor) | 2 | 2/2 (1 shown) | 2 |
| Fig. 5 (Strain 1+ymUkG1 donor) | 2 | 2/2 (1 shown) | 2 |
| Fig. 5 (Strain 2+eGFP donor) | 2 | 2/2 (1 shown) | 2 |
| Fig. 5 (Strain 2+ymUkG1 donor) | 2 | 2/2 (1 shown) | 2 |
| Fig. 5 (Strain 3+eGFP donor) | 2 | 2/2 (1 shown) | 2 |
| Fig. 5 (Strain 3+ymUkG1 donor) | 2 | 2/2 (1 shown) | 2 |
| Fig. 5 (Strain 4+mCherry donor) | 2 | 2/2 (1 shown) | 2 |
| Fig. 5 (Strain 5+mCherry donor) | 2 | 2/2 (1 shown) | 2 |
| Fig. 6 (Diploid control) | 2 | 2/2 | ND |
| Fig. 6 (Diploid active gene drive) | 2 | 2/2 | ND |

^1^Data from Figure 1 uses traditional HR-based integration methods in yeast and not Cas9.

^2^ND, not determined.

^3^DNA sequencing revealed two additional (unintended) modifications to the MSO open reading frame.

^4^In addition to the *MSO1*, *BNI4*, and *APL1* loci, we examined N-terminal tagging using our method at 37 other loci including: *BUD6, KEL1, BNI1, BNR1, BOI1, AXL2, ELM1, GIC2, GIC1, BUD4, BIL1***,** *MLC2, BUD3***,** *SYP1***,** *CAF120***,** *SLG1***,** *APL3***,** *NBA1***,** *DSE3***,** *YAP1802***,** *FIR1***,** *MSB1***,** *SHO1***,** *MYO4***,** *SPH1***,** *FLC2***,** *YAP1801***,** *BEM2***,** *AIM44***,** *DSE1***,** *APM4***,** *DBF2***,** *BUD2***,** *FLC1***,** *EPO1***,** *MSB4***,** *YEL1.* The same methodology described in Fig. 4 was used—only a single isolate was tested by PCR and DNA sequencing following testing on G418 medium. Genes colored in red text did not contain a proper N-terminal tag (due to lack of PCR product, lack of inserted tag, or added mutation or frameshift in the final product). *CAF120* (purple text) contained the proper N-terminal tag, but had a shortened (14 residue) flexible linker.

**Works Cited**

Finnigan, G. C., J. Takagi, C. Cho and J. Thorner, 2015 Comprehensive Genetic Analysis of Paralogous Terminal Septin Subunits Shs1 and Cdc11 in *Saccharomyces cerevisiae*. Genetics 200**:** 821-841.

Finnigan, G. C., and J. Thorner, 2015 Complex in vivo Ligation Using Homologous Recombination and High-efficiency Plasmid Rescue from *Saccharomyces cerevisiae*. Bio-protocol 5**:** e1521. <http://www.bio-protocol.org/e1521>

Goldstein, A. L., and J. H. McCusker, 1999 Three new dominant drug resistance cassettes for gene disruption in *Saccharomyces cerevisiae*. Yeast 15**:** 1541-1553.
